# Synthetic Tabular Data Based on Generative Adversarial Networks in Health Care: Generation and Validation Using the Divide-and-Conquer Strategy

**DOI:** 10.2196/47859

**Published:** 2023-11-24

**Authors:** Ha Ye Jin Kang, Erdenebileg Batbaatar, Dong-Woo Choi, Kui Son Choi, Minsam Ko, Kwang Sun Ryu

**Affiliations:** 1 Department of Applied Artificial Intelligence Hanyang University Ansan Republic of Korea; 2 Department of Cancer AI & Digital Health Graduate School of Cancer Science and Policy National Cancer Center Gyeonggi-do Republic of Korea; 3 National Cancer Data Center National Cancer Control Institute National Cancer Center Gyeonggi-do Republic of Korea; 4 Department of Cancer Control and Policy Graduate School of Cancer Science and Policy National Cancer Center Gyeonggi-do Republic of Korea; 5 Department of Human-Computer Interaction Hanyang University Ansan Republic of Korea

**Keywords:** generative adversarial networks, GAN, synthetic data generation, synthetic tabular data, lung cancer, machine learning, mortality prediction

## Abstract

**Background:**

Synthetic data generation (SDG) based on generative adversarial networks (GANs) is used in health care, but research on preserving data with logical relationships with synthetic tabular data (STD) remains challenging. Filtering methods for SDG can lead to the loss of important information.

**Objective:**

This study proposed a divide-and-conquer (DC) method to generate STD based on the GAN algorithm, while preserving data with logical relationships.

**Methods:**

The proposed method was evaluated on data from the Korea Association for Lung Cancer Registry (KALC-R) and 2 benchmark data sets (breast cancer and diabetes). The DC-based SDG strategy comprises 3 steps: (1) We used 2 different partitioning methods (the class-specific criterion distinguished between survival and death groups, while the Cramer V criterion identified the highest correlation between columns in the original data); (2) the entire data set was divided into a number of subsets, which were then used as input for the conditional tabular generative adversarial network and the copula generative adversarial network to generate synthetic data; and (3) the generated synthetic data were consolidated into a single entity. For validation, we compared DC-based SDG and conditional sampling (CS)–based SDG through the performances of machine learning models. In addition, we generated imbalanced and balanced synthetic data for each of the 3 data sets and compared their performance using 4 classifiers: decision tree (DT), random forest (RF), Extreme Gradient Boosting (XGBoost), and light gradient-boosting machine (LGBM) models.

**Results:**

The synthetic data of the 3 diseases (non–small cell lung cancer [NSCLC], breast cancer, and diabetes) generated by our proposed model outperformed the 4 classifiers (DT, RF, XGBoost, and LGBM). The CS- versus DC-based model performances were compared using the mean area under the curve (SD) values: 74.87 (SD 0.77) versus 63.87 (SD 2.02) for NSCLC, 73.31 (SD 1.11) versus 67.96 (SD 2.15) for breast cancer, and 61.57 (SD 0.09) versus 60.08 (SD 0.17) for diabetes (DT); 85.61 (SD 0.29) versus 79.01 (SD 1.20) for NSCLC, 78.05 (SD 1.59) versus 73.48 (SD 4.73) for breast cancer, and 59.98 (SD 0.24) versus 58.55 (SD 0.17) for diabetes (RF); 85.20 (SD 0.82) versus 76.42 (SD 0.93) for NSCLC, 77.86 (SD 2.27) versus 68.32 (SD 2.37) for breast cancer, and 60.18 (SD 0.20) versus 58.98 (SD 0.29) for diabetes (XGBoost); and 85.14 (SD 0.77) versus 77.62 (SD 1.85) for NSCLC, 78.16 (SD 1.52) versus 70.02 (SD 2.17) for breast cancer, and 61.75 (SD 0.13) versus 61.12 (SD 0.23) for diabetes (LGBM). In addition, we found that balanced synthetic data performed better.

**Conclusions:**

This study is the first attempt to generate and validate STD based on a DC approach and shows improved performance using STD. The necessity for balanced SDG was also demonstrated.

## Introduction

Machine learning (ML) techniques have been applied in health care with remarkable success over the past decade. ML has the potential to improve tasks in various fields in the medical industry [1]. Analysis of clinical data to predict risk factors and degrees of association between diseases [2] is one of the major advancements achieved using ML. However, the application of ML in real-world clinical environments remains difficult owing to clinical limitations, such as data scarcity, data privacy, and data imbalance [3]. In this context, generative adversarial networks (GANs) [4] have emerged as one of the most important types of ML-based generative models in health care [5].

GAN algorithms generate large amounts of synthetic patient data, which can serve as an appropriate alternative to real data [6-8]. A GAN comprises 2 models trained using an adversarial process, in which one model—the “generator”—generates synthetic data, while the other—the “discriminator”—distinguishes between real and synthetic data. Conventional GAN algorithms have been enhanced and repurposed for clinical tabular data [9-11]. In addition, GANs alleviate clinical limitations and facilitate the application of ML in health care [3,12]. Beaulieu-Jones et al [13] used the auxiliary classifier generative adversarial network (ACGAN) to generate synthetic SPRINT (Systolic Blood Pressure Intervention Trial) data for privacy-preserving data sharing. Baowaly et al [14] generated synthetic electronic health record data using the medical generative adversarial network (MedBGAN) to resolve the data-sharing problem. Izonin et al [15] created an enlarged data set based on a GAN to improve the accuracy of diagnostics tasks. Wang et al [16] developed a framework to generate and evaluate synthetic data, while simultaneously preserving the complexities of real data and ensuring privacy.

Nevertheless, the application of existing models and algorithms, which are not tailor-made for tabular health care data, to synthetic data generation (SDG) in this field remains unsuitable. Some do not consider the characteristics of health care tabular data [17]. To generate synthetic tabular data (STD), while preserving data with logical relationships, the relationships between columns in the original data (OD) must be considered. The OD have a logical relationship between each column: For example, measurement of the drinking attribute is performed using the binary classification “yes” or “no.” If the value of this attribute is “no” in some records, the corresponding value of the subattribute “How much do you drink per week?” must be 0. However, poorly designed GANs may generate synthetic data containing impossible values, for example, a record indicating “drinking: no” and “How much do you drink per week?: 10.” This can potentially affect the quality of the generated synthetic data and make them unreliable for certain analyses. To prevent this, filtering methods in GANs have been developed. Both the conditional tabular generative adversarial network (CTGAN) [18] and the copula generative adversarial network (CopulaGAN) [19] use conditional sampling (CS) as a filtering method to forcibly express logical relationships. CS is a method used in the CTGAN and CopulaGAN. CS works through a process of rejection sampling, in which multiple iterations of row sampling occur until a satisfactory row that meets the established conditions is obtained. The performance is also compared on balanced and imbalanced synthetic data sets. However, filtering methods exclude record data based on predefined condition columns after STD generation, ignoring meaningful information contained in the excluded records. To mitigate this risk, it is important to carefully consider the specified conditions and to ensure that they are representative of the broader population.

In addition, although it is generally accepted that balanced data perform better in classification, there has been little research based on experiments that clearly demonstrate how much class-balanced tabular synthetic data are required to improve model performance. Therefore, our experiments suggest that when creating a reference table, we should consider how much data to create so that the classes are balanced when generating synthetic data.

In this study, we proposed an SDG framework to overcome the aforementioned challenges in clinical data generation. The remainder of the paper is organized as follows. The *Methods* section describes the basic characteristics of the study population and the divide-and-conquer (DC)–based SDG strategy, defines the division criteria, presents the problem statement for a filtering method, and presents the SDG process and verification methods. The *Results* section compares the prediction performances of the proposed approach and CS. Moreover, the quality of the generated STD is estimated. Finally, the *Discussion* section elaborates further on the experimental design, results, limitations, and conclusions.

## Methods

### Ethical Considerations

The study design was approved by the Ethics Review Board of the National Cancer Center Institutional Review Board (IRB no: NCC2022-0281).

### Research Framework

**Figure 1:**
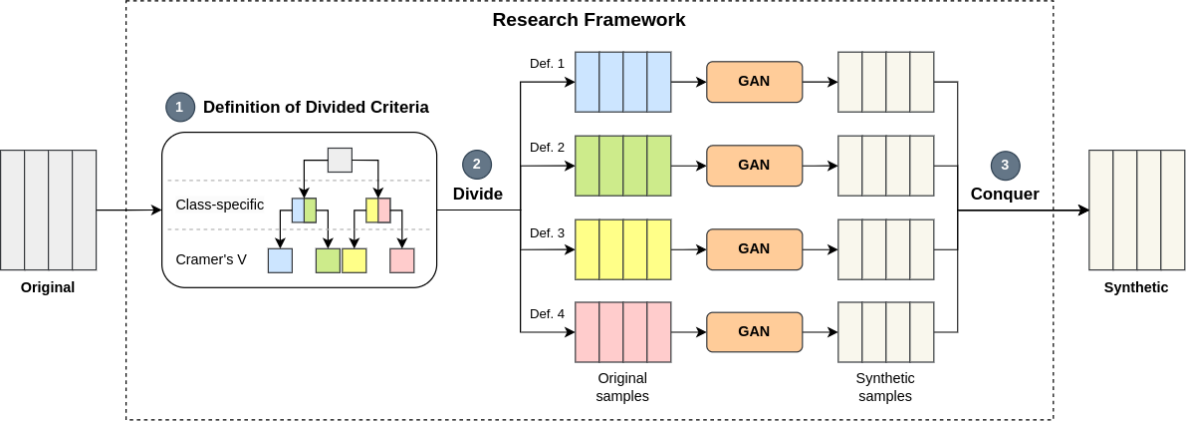
DC-based research framework using GANs. Def.: definition; GAN: generative adversarial network.

The DC-based research framework, as depicted in Figure 1, generates STD, while preserving data with logical relationships to enable comparison in terms of data reliability and investigation of the factors affecting ML model performance. In the division step, the entire data set was divided into several subsets based on the division criteria. In the conquer step, different subsets generated via the GAN were combined into 1. Following STD generation, model performances achieved using classification algorithms, such as the decision tree (DT), the random forest (RF), Extreme Gradient Boosting (XGBoost), and the light gradient-boosting machine (LGBM), in both DC- and CS-based approaches of the CTGAN and CopulaGAN were compared. Moreover, ML model performance on balanced synthetic data and imbalanced synthetic data was also compared.

### Definition of the Cramer V and Class-Specific Division Criteria

In this study, the division criteria involved 2 main components, class-specific and the Cramer V criteria. The class-specific criterion enabled the selection of different feature subsets for all classes, allowing for subsamples that were tailored to the unique characteristics and behavior of each class. Meanwhile, the high correlation-based criterion identified variables with high correlation scores by computing the Cramer V correlation matrix. The reason for using the Cramer V criterion as the second division criterion was to preserve the logical relationships in the OD in the synthetic data. These variables were then used as the basis for defining logical relationships of data that would guide the division of the data set into subsamples. These 2 criteria provided a robust and effective approach to analyzing the data and identifying meaningful patterns and relationships within them.

#### Class-Specific Criterion

We used a class-specific division criterion [20], which enabled the selection of different feature subsets for all classes. This yielded a comprehensive list of data set allocation attributes and values by deconstructing the OD set into smaller, more refined subsets. These subsets were subsequently classified based on the dependent classes between different classes, which in turn represented unique sets of class-based criteria. This enabled the selection of a different feature subset for each class. This approach is particularly useful when dealing with data sets comprising classes with unique characteristics and behaviors. The class-specific criterion enables the creation of subsamples tailored to each class, in turn leading to more accurate predictions and better insights.

#### Cramer V Criterion

We used the Cramer V correlation to identify high correlation patterns in the data set. The Cramer V criterion is a measure of substantive importance used to measure how strongly 2 categorical fields are related to each other. Its value ranges from 0 to 1, where 0 represents no association between the categorical variables and 1 represents complete association in the contingency table. The Cramer V correlation coefficient can be calculated using the formula provided in Equation 1:



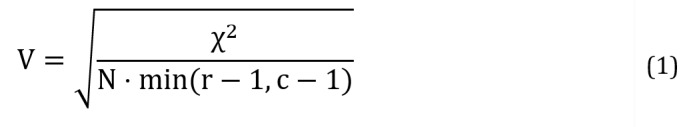



where V denotes the Cramer V correlation coefficient, *χ*^2^ denotes the chi-square statistic of the contingency table, N denotes the total number of observations in the contingency table, r denotes the number of rows in the contingency table, and c denotes the number of columns in the contingency table.

The steps in calculating the Cramer V correlation coefficient are as follows:

- Step 1: Calculate χ^2^, which is a measure of the association or independence between 2 categorical variables represented in a contingency table and quantifies how much the observed frequencies would deviate from the expected frequencies if the variables were independent. A higher χ^2^ value suggests a stronger association between the variables.
- Step 2: Determine the scaling factor, which is necessary to normalize the Cramer V correlation coefficient. The scaling factor is calculated as min(r – 1, c – 1), where (r – 1) and (c – 1) represent the degrees of freedom associated with the rows and columns in the contingency table, respectively. By taking the minimum of (r – 1, c – 1), the formula scales χ^2^ appropriately, avoiding overestimation of the association in situations in which one variable has more categories than the other. The purpose of this scaling factor is 2-fold: (1) It ensures that the Cramer V correlation coefficient, which is the final result, falls within the range of 0-1. This range makes the coefficient interpretable and suitable for comparison across different data sets. (2) It normalizes χ^2^ by considering the dimensions of the contingency table (number of rows and columns) and the degrees of freedom. This normalization avoids overestimating the strength of the association in situations in which one variable has more categories than the other.
- Step 3: Calculate the Cramer V correlation coefficient. The final result is a value that ranges from 0 to 1, where 0 indicates no association (variables are independent) and 1 represents a perfect association in the contingency table. This coefficient helps interpret and compare the degree of association across different data sets and contingency tables.

We computed the Cramer V correlation matrix for all pairs of categorical variables in the data set. If the variables had a score of 1, it meant that these variables were representative of the characteristic of the OD. These highly related variables should certainly be represented in the synthetic data for fidelity, which is a statistical measure of similarity. In other words, a Crammer V score of 1 was the threshold and variables scoring 1 were used as the division criteria.

### Logical STD in Health Care

National clinical databases differ based on the organization, but clinical data sets are valuable resources [17] that provide insights into improving patient care and organizational efficiency. However, the quality and quantity of clinical data can be limited, especially in cases where data privacy concerns restrict access to real-world data sets. SDG has emerged as a promising solution to this problem, enabling organizations to create new data sets that capture the characteristics of real-world data accurately. However, illogical STDs are frequently generated when simply designed GAN models are used, which induces learning of irregular relationships between the main attribute and its subattributes.

#### Divide-and-Conquer Approach for Logical STD

As mentioned in the previous section, CS can be a useful approach for generating synthetic data. However, it suffers from the risk of information loss owing to the dependence on condition columns. This is particularly pertinent in cases involving a tabular health care data set, because each of its columns contains significant information [21]. To address these issues, we proposed a DC-based alternative approach.

DC is an easily implementable computing approach [22]. It divides an original problem into several subproblems, analyzes them separately, and then combines the results to obtain the overall solution [23]. DC can be used to generate high-quality synthetic data by dividing the OD set into smaller subsets based on a set of predefined division criteria. This facilitates the specification of complex or multidimensional conditions, while simultaneously reducing the risk of information loss. We followed these steps in the DC approach to generate high-quality synthetic data:

- Step 1: To define the division criteria, we used a methodology involving a class-specific criterion and the Cramer V correlation coefficient. This approach enabled the selection of a different feature subset for each class and consideration of the relationships between different variables to determine the degrees of association.
- Step 2: Based on the defined division criteria, we divided the OD into subsets containing each criterion separately as a specific pattern or relationship. Subsequently, these subsets were used to train GANs on specific patterns and relationships. As a result, the generated STD preserved the patterns and relationships of each subset.
- Step 3 (conquer): The synthetic data corresponding to the different subsets were combined. The generated STD preserved the underlying patterns and relationships within each subset of the OD.

This DC-based approach enabled STD that reflected the underlying patterns and relationships within each subset of the OD accurately.

### Generation and Verification of STD

#### Generative Adversarial Networks

In this study, we used 2 generative models, the CTGAN and CopulaGAN, to generate synthetic data:

- The CTGAN is specifically designed for generating synthetic data from tabular data. The CTGAN exhibits several unique features, including the ability to handle discrete and continuous features, the use of conditional generators, and sampling-specific training to avoid mode collapse and data imbalance problems.
- CopulaGAN uses copulas to model the joint distribution of input features. Copulas are statistical models that describe the dependence structures between random variables, and they have been demonstrated to be effective in modeling complex dependencies between features in real-world data sets.

#### Prediction Methods

In this study, we validated mortality prediction performance using 4 different classifiers: DT, RF, XGBoost, and LGBM. We used these classifiers to train the ML models and evaluated their performances in predicting mortality in our data set. A sufficiently large training data set was generated in the experiment, and the 4 ML algorithms were used to generate mortality prediction models for patients with non–small cell lung cancer (NSCLC).

- The DT [24] is a commonly used tool. Essentially, a DT is a supervised model that classifies or performs predictions on data sets based on rules in the data. To reach a decision, a DT learns by posing binary questions, which can be represented using a tree structure. The data set is divided hierarchically to contain the greatest amount of information, while branching from the root node. The data are split repeatedly until each segmented region contains a single target value.
- The RF [25] was developed by Leo Breiman and Adele Cutler. It is an extension of the bagging method, which combines the output of multiple DTs to yield a single result. In other words, DTs consider all possible feature splits, while RFs only select a subset of these features. Each tree in an RF ensemble consists of a training set with bootstrap samples. One-third of it is set aside as testing data, known as the out-of-bag (OOB) sample. For a regression task, individual DTs are averaged, and for a classification task, a majority vote is used to obtain the predicted class. Finally, the OOB sample is used for cross-validation.
- XGBoost [26], a scalable tree-boosting system, is used to solve both classification and regression problems and is a popular algorithm because of its good performance and resource efficiency. XGBoost was developed to handle sparse data. It is an innovative tree learning algorithm that handles instance weights in inexact tree learning, which is a justified weighted quantile sketch procedure. XGBoost enables parallel and distributed computing, which accelerates both learning and model exploration. It exploits out-of-core computation, which enables the construction of an end-to-end system.
- The LGBM [27] is a tree-based learning algorithm with a gradient-boosting framework. In an LGBM, the tree expands vertically compared to other algorithms, in which it expands horizontally. In other words, an LGBM uses a leaf-wise structure, while other algorithms use level-wise structures. An LGBM chooses a leaf with the maximum delta loss to expand, enabling the leaf-wise algorithm to reduce greater loss than its level-wise counterparts.

### Experimental Setting

#### Data Set

##### Study Population

The Korea Association for Lung Cancer Registry (KALC-R) was developed in cooperation with the Korean Central Cancer Registry and the Lung Cancer Registration Committee. Approximately 10% of NSCLC cases listed in this registry were surveyed in this study. The survey population comprised 13 regional cancer centers and 39 hospitals with numerous registrations [28,29]. Our study used a nonduplicate sample comprising data of 5281 subjects obtained from the KALC-R 2014 and 2015 data sets. Entries with missing and unknown values for weight, height, forced vital capacity (FVC), diffusing capacity of the lungs for carbon monoxide (DLCO), the chemotherapy tumor, extent of spread to lymph nodes, and presence of metastasis (TNM) stage (n=1773, 33.6%), and NSCLC (n=1204, 22.8%) were excluded. This study population (N=2304) was then divided into a development group (n=1616, 70.1%) and a validation group (n=688, 29.9%) via stratified random sampling. The development group used GAN learning for STD and model training for short-term prediction models. The validation group evaluated model performance in terms of ML models in accordance with the quality of prediction. The primary endpoint was defined to be 1 year after the diagnosis of NSCLC for all causes of death.

Moreover, we selected 2 well-known publicly available data sets: the breast cancer data set from the University of California, Irvine (UCI) *Machine Learning Repository* [30] and the diabetes data set [31]. The breast cancer data set comprises real patient data obtained from the Institute of Oncology, Ljubljana, in 1988, aimed at predicting the recurrence of breast cancer. The diabetes data set describes the clinical care at 130 US hospitals and integrated delivery networks from 1999 to 2008. The classification task predicts whether a patient will be readmitted within 30 days.

##### Comparison of Basic Characteristics

We analyzed the fundamental characteristics of the data sets of patients of NSCLC, breast cancer, and diabetes and further compared the following basic characteristics of different groups for NSCLC survival, breast cancer recurrence, and diabetes readmission in the development data:

- NSCLC: The NSCLC data set exhibited similar distributions across various variables, including age, height, weight, FVC, forced expiratory volume in 1 second (FEV1), DLCO, smoking history (pack-years), gender, Eastern Cooperative Oncology Group (ECOG) performance status, pathological type, epidermal growth factor receptor (EGFR) mutation status, anaplastic lymphoma kinase immunohistochemistry (ALK IHC), anaplastic lymphoma kinase fluorescence in situ hybridization (ALK FISH), cancer stage, curative operations, radiotherapy (RT), chemotherapy, and cause of death. The survival group exhibited lower values for age, height, smoking history (past and current), ECOG performance status, specific cancer types (squamous cell carcinoma, large-cell carcinoma), cancer stage, and palliative chemotherapy compared to the death group. Conversely, the survival group had higher values for weight, FVC, FEV1, DLCO, DLCO percentage, nonsmoking status, adenocarcinoma, positive EGFR mutation, positive ALK IHC, positive ALK FISH, curative operations, RT, and curative chemotherapy.
- Breast cancer: The breast cancer data set also showed comparable distributions for variables, such as age, menopausal status, tumor size, invasive/involved (inv) nodes, node caps, malignancy degree, breast location, breast quadrant, irradiation, and recurrence events. The recurrence group had lower values for age, early menopause (at or before age 40 years), tumor size, inv nodes, node caps, lower malignancy degrees (1 and 2), right breast, breast quadrant, and irradiation compared to the nonrecurrence group. In contrast, the recurrence group had higher values for premenopausal status, malignancy degree (3), and left breast.
- Diabetes: In the diabetes data set, basic characteristics revealed similar distributions for variables, such as hospital stay duration, laboratory procedures, medications, outpatient visits, emergency visits, inpatient stays, diagnoses, race, gender, age, medical specialty, glycated hemoglobin (A1C) results, diabetes medications, and readmission events. The readmitted group displayed lower values for the number of procedures, certain demographics (African American and other races, males), age, medical specialty (except others), A1C result (except none), insulin usage, changes in treatment, and certain diagnoses compared to the nonreadmitted group. Conversely, the readmitted group showed higher values for time spent in the hospital, number of lab procedures, number of medications, number of outpatient visits, number of emergency visits, number of inpatient stays, number of diagnoses, Caucasian race, females, other medical specialties, no A1C result, and diabetes medication (metformin, glipizide, glyburide) usage.

A detailed comparison of the characteristics of different data sets is presented in Multimedia Appendix 1.

#### Data Split

##### Division Criteria Analysis

**Figure 2:**
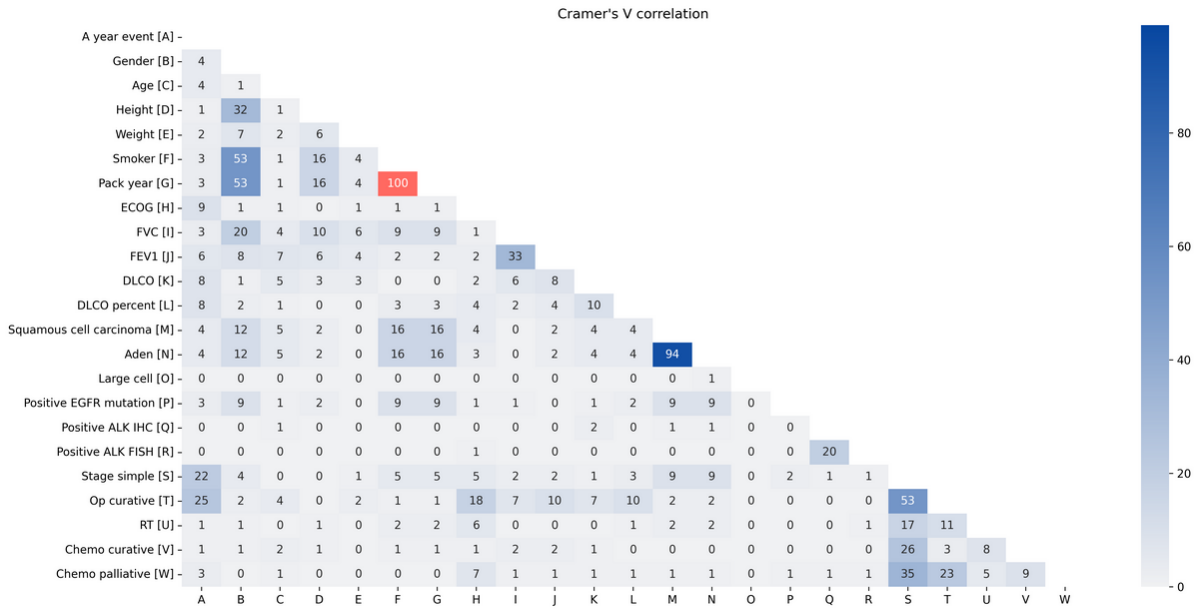
Cramer V correlation coefficient of lung cancer data. Aden: adenocarcinoma; ALK FISH: anaplastic lymphoma kinase fluorescence in situ hybridization; ALK IHC: anaplastic lymphoma kinase immunohistochemistry; chemo curative: curative chemotherapy; chemo palliative; palliative chemotherapy; DLCO: diffusing capacity of the lungs for carbon monoxide; ECOG: Eastern Cooperative Oncology Group; EGFR: epidermal growth factor receptor; FEV1: forced expiratory volume in 1 second; FVC: forced vital capacity; OP curative: curative operations; RT: radiotherapy.

First, we used class-specific division on the “adverse event” feature, which represents dependent classes, and divided the data into death and survival groups. Next, the Cramer V correlation coefficient was applied after converting all variables into the categorical format. The highest correlation score (V=1, highlighted in red in Figure 2) in NSCLC data was observed between smoking status and pack-years of smoking. This indicates a strong association between these 2 variables, suggesting that individuals who smoke more frequently are more likely to be current or former smokers. Therefore, the “smoker” feature was identified as a key division criterion in our data set. Following the definition, we created a subsample consisting of only those patients in the data set who were smokers and had a pack-year of more than 0. In conclusion, the data were divided into distinct smoker and nonsmoker groups. In other data sets, we did not find a high correlation score, as seen in Multimedia Appendix 2.

By applying the aforementioned division criteria, we obtained 4 small samples from the data set: death-smoker, death-nonsmoker, survival-smoker, and survival-nonsmoker. These samples were used for further performance validation and fidelity tests. Finally, our data set was successfully partitioned for the purposes of our study.

#### Metrics

##### Performance Evaluation Metrics

The ability of the synthetic data to achieve good predictive performance in downstream modeling tasks was evaluated using metrics, such as the area under the curve (AUC) and the *F*_1_-score. This is important as the generated synthetic data must be useful for predictive modeling for it to lead to actionable insights.

The AUC is a performance metric that measures the ability of a binary classifier to distinguish between positive and negative classes. It is calculated as the area under the receiver operating characteristic (ROC) [32] curve. ROC curves are graphical representations of the relationship between the false-positive rate (FPR) and the true-positive rate (TPR), plotted along the x and y axes, respectively. The AUC ranges from 0 to 1, where 1 represents perfect classification performance and 0.5 indicates perfectly random performance. The formula for the AUC is given by Equation 2:



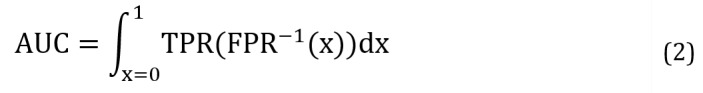



The *F*_1_-score is a measure of the balance between precision and recall, where precision is defined as the fraction of true positives among all predicted positives and recall is defined as the fraction of true positives among all actual positives. The *F*_1_-score ranges from 0 to 1, where 1 represents perfect precision and recall and 0 represents the worst-possible scores. The formula for the F1-score is given by Equation 3:



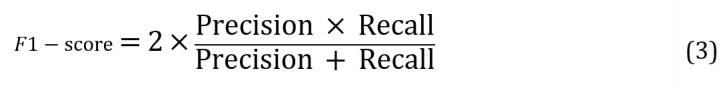



##### Quality Evaluation Metrics

Shape and pair trend metrics [33] are commonly used to evaluate the fidelity of STD, that is, their similarity to the distribution of real-world data. Shape refers to the overall distributional shape of a data set, including factors such as the degree of skewness or kurtosis. Pair trend, in contrast, refers to the relationship between pairs of features in the data set. Although shape analysis focuses on individual features of a data set, pair trend analysis provides information about the overall structure and relationships between features. To evaluate the distribution shapes of numerical columns, we used the Kolmogorov-Smirnov statistic (KSS), which is defined as the maximum difference between the cumulative distribution functions (CDFs). CDFs determine the probability that a random observation taken from the population will be less than or equal to a certain value. Conversely, for categorical columns, we used the total variation distance (TVD). The formulas for KSS and TVD scores are given by Equations 4 and 5, respectively, where x represents a single column. Similarly, pair trend metrics were considered to consist of 2 measures, correlation similarity and contingency similarity, for numerical and categorical columns, respectively. Equations 6 and 7 present the formulas for correlation and contingency similarity scores, respectively, where x and y together denote a pair of columns.



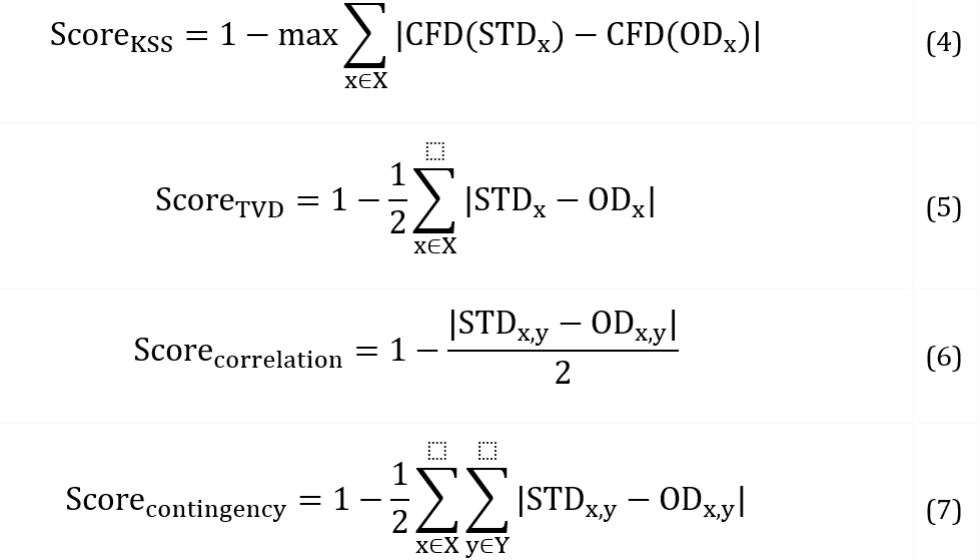



By computing separate scores for each column and pair of columns, an individual score was obtained for every column. The final score (value between 0 and 1, with a higher score representing higher quality) was obtained by averaging individual scores. These statistical metrics assessed the similarity or dissimilarity between the distributions of samples in the OD and STD. They provided quantitative measures for evaluating how closely the data sets matched in terms of distribution shapes, relationships between variables, and contingency structures. The final aggregated score represented the overall quality or fidelity of the OD compared to the STD. All 4 measures used for evaluating the fidelity of the OD compared to the STD are summarized in Table 1.

**Table 1:** Comparison of measures for evaluating fidelity between the OD^a^ and the STD^b^.

Measure	Purpose	Application	Computation	Interpretation
KSS^c^	Measures the similarity and dissimilarity of distribution shapes between OD and STD	Numerical	Calculates the maximum difference between the CDFs^d^ of OD and STD	A higher score indicates greater dissimilarity in distribution shapes, with 0 representing identical distributions.
TVD^e^	Quantifies the difference between probability distributions of categorical data in OD and STD	Categorical	Measures the “closeness” between probability mass functions of OD and STD distributions	A score of 0 implies identical distributions, while higher scores indicate increasing dissimilarity.
Correlation	Evaluates the similarity or dissimilarity of relationships between pairs of numerical variables between OD and STD	Numerical	Measures the absolute squared difference between correlation coefficients of OD and STD pairs	A score of 0 indicates perfect similarity in relationships, while higher scores suggest weaker similarity or even dissimilarity.
Contingency	Assesses the similarity of relationships between pairs of categorical variables in OD and STD	Categorical	Calculates the sum of absolute differences between corresponding cells in contingency tables (cross-tabulations) of OD and STD	A score of 0 signifies perfect similarity in contingency structures, while higher scores indicate less similarity.

^a^OD: original data.

^b^STD: synthetic tabular data.

^c^KSS: Kolmogorov-Smirnov statistic.

^d^CDF: cumulative distribution function.

^e^TVD: total variation distance.

## Results

### Generation and Validation of STD

To generate logical STD, we trained the CTGAN and CopulaGAN using existing CS filtering. Next, we used the proposed DC-based method before training the CTGAN and CopulaGAN without CS filtering. The volume of the generated data set was set to 5000. Moreover, we generated 2 types of STDs, a balanced data set with equal class distributions between samples in a 50:50 ratio and an imbalanced data set with a 1:100 class distribution ratio between samples (ie, each dependent variable occurred 100 times less frequently than its counterpart).

To verify the superiority of the proposed DC-based method in the generation of logical STD, we evaluated each STD item using 4 different ML models (DT, RF, XGBoost, and LGBM). Table 2 presents the validation results of the DT classifier. The AUC and *F*_1_-score values of the NSCLC, breast cancer, and diabetes OD were 66.06% and 66.11%, 61.14% and 49.64%, and 65.58% and 47.82%, respectively. The highest AUC of 74.87% was achieved by generating synthetic data using the DC strategy with the CopulaGAN, while the highest *F*_1_-score of 71.99% was achieved using the DC strategy with the CTGAN for NSCLC data. The highest AUC of 73.31% was achieved by generating synthetic data using the DC strategy with the CTGAN, while the highest *F*_1_-score of 68.92% was achieved using the DC strategy with the CopulaGAN for breast cancer data. The highest AUC of 61.57% was achieved by generating synthetic data using the DC strategy with the CTGAN, while the highest *F*_1_-score of 53.8% was achieved using the DC strategy with the CopulaGAN for diabetes data.

The validation results obtained using the RF classifier are presented in Table 3. The AUC and *F*_1_-score values of the NSCLC, breast cancer, and diabetes OD were 84.81% and 72.74%, 69.37% and 60.01%, and 62.13% and 47.73%, respectively. The highest AUC and *F*_1_-score of 85.61% and 75.09%, respectively, were achieved by generating synthetic data using the DC strategy with the CTGAN for NSCLC data. The highest AUC and *F*_1_-score of 78.05% and 71.03%, respectively, were achieved by generating synthetic data using the DC strategy with the CTGAN for breast cancer data. The highest AUC and *F*_1_-score of 59.98% and 53.47%, respectively, were achieved by generating synthetic data using the DC strategy with the CTGAN for diabetes data.

Table 4 presents the validation results obtained using the XGBoost classifier. The AUC and *F*_1_-score values of the NSCLC, breast cancer, and diabetes OD were 83.07% and 71.14%, 71.21% and 62.89%, and 67.02% and 48.91%, respectively. The highest AUC and *F*_1_-score of 85.20% and 74.78%, respectively, were achieved by generating synthetic data using the DC strategy with the CTGAN for NSCLC data. The highest AUC and *F*_1_-score of 77.86% and 70.58%, respectively, were achieved by generating synthetic data using the DC strategy with the CTGAN for breast cancer data. The highest AUC and *F*_1_-score of 60.18% and 53.93%, respectively, were achieved by generating synthetic data using the DC strategy with the CTGAN for diabetes data.

Finally, Table 5 presents the validation results obtained using the LGBM classifier. The AUC and *F*_1_-score values of the NSCLC, breast cancer, and diabetes OD were 84.09% and 71.30%, 75.84% and 62.07%, and 67.88% and 47.89%, respectively. The highest AUC and *F*_1_-score of 85.14% and 74.40%, respectively, were achieved by generating synthetic data using the DC strategy with the CTGAN for NSCLC data. The highest AUC and *F*_1_-score of 77.86% and 70.58%, respectively, were achieved by generating synthetic data using the DC strategy with the CTGAN for breast cancer data. The highest AUC and *F*_1_-score of 60.18% and 53.93%, respectively, were achieved by generating synthetic data using the DC strategy with the CTGAN for diabetes data.

In general, the results demonstrate that STD generated using the DC approach had the best quality in terms of the AUC and *F*_1_-score. Moreover, higher performance was observed when STD were generated solely using the DC approach compared to STD obtained using the original training data. Moreover, balanced data sets consistently exhibited better performance than imbalanced ones.

In addition, we assessed the quality of the generated STD by evaluating their fidelity with respect to shape and pair trend metrics. The results are presented in Tables 6-8. The DC strategy with the CTGAN achieved the highest mean shape score of 90.49 (SD 0.07), 91.71 (SD 0.12), and 98.60 (SD 0.13), the highest mean pair trend score of 83.92 (SD 0.10), 82.72 (SD 0.13), and 96.70 (SD 0.26), and the highest mean overall score of 87.20 (SD 0.08), 87.21 (SD 0.09), and 97.65 (SD 0.27) on the NSCLC, breast cancer, and diabetes data sets, respectively. These findings suggest that the DC strategy with the CTGAN could be a promising approach for generating synthetic data with high fidelity. Moreover, we carried out a number of visualization experiments comparing the OD and the STD, as shown in Multimedia Appendix 3.

**Table 2:** Validation results obtained using the DT^a^ classifier: mean (SD) values of 5 experiments.

Data type, GAN^b^, and approach		NSCLC^c^		Breast cancer		Diabetes	
		AUC^d^	*F*_1_-score	AUC	*F*_1_-score	AUC	*F*_1_-score
OD^e^		66.06 (1.31)	66.11 (1.30)	61.14 (1.66)	49.64 (1.06)	65.58 (0.13)	47.82 (0.04)
**Balanced STD^f^**							
	CTGAN^g^, no condition	63.46 (1.91)	61.87 (1.83)	59.10 (2.17)	62.20 (1.93)	57.19 (0.25)	49.16 (0.10)
	CTGAN, CS^h^	59.95 (1.26)	58.65 (2.44)	64.65 (1.66)	56.67 (1.31)	58.93 (0.18)	47.89 (0.09)
	CTGAN, DC^i^	74.50 (1.23)	71.99 (0.77)^j^	73.31 (1.11)^j^	67.10 (1.83)	61.57 (0.09)^j^	50.45 (0.05)
	CopulaGAN^k^, no condition	66.11 (1.32)	65.16 (1.27)	59.82 (1.69)	52.32 (2.11)	58.93 (0.24	47.13 (0.04)
	CopulaGAN, CS	63.87 (2.02)	63.07 (1.87)	67.96 (2.15)	62.20 (1.93)	60.08 (0.17)	49.77 (0.04)
	CopulaGAN, DC	74.87 (0.77)^j^	70.54 (0.76)	70.66 (0.85)	68.92 (1.78)^j^	60.61 (0.06)	53.80 (0.08)^j^
**Imbalanced STD**							
	CTGAN, no condition	50.93 (0.89)	44.99 (1.97)	52.48 (0.09	38.75 (0.51	57.60 (0.14	47.09 (0.01
	CTGAN, CS	50.64 (0.80)	44.80 (1.48)	52.48 (0.09)	38.75 (0.51)	58.34 (0.24)	47.09 (0.01)
	CTGAN, DC	57.99 (2.06)	57.78 (2.81)	56.00 (0.31)	38.75 (0.51)	60.52 (0.05)	50.36 (0.10)
	CopulaGAN, no condition	52.32 (1.05)	48.38 (1.92)	53.81 (0.63)	38.75 (0.51)	57.10 (0.21)	47.09 (0.01)
	CopulaGAN, CS	52.06 (0.91)	47.62 (1.70)	55.50 (1.01)	38.75 (0.51)	56.21 (0.24)	47.09 (0.01)
	CopulaGAN, DC	55.86 (3.10)	54.12 (4.60)	57.95 (0.76)	38.75 (0.51)	59.38 (0.15)	50.22 (0.08)

^a^DT: decision tree.

^b^GAN: generative adversarial network.

^c^NSCLC: non–small cell lung cancer.

^d^AUC: area under the curve.

^e^OD: original data.

^f^STD: synthetic tabular data.

^g^CTGAN: conditional tabular generative adversarial network.

^h^CS: conditional sampling.

^i^DC: divide and conquer.

^j^The best results.

^k^CopulaGAN: copula generative adversarial network.

**Table 3:** Validation results obtained using the RF^a^ classifier: mean (SD) values of 5 experiments.

Data type, GAN^b^, and approach		NSCLC^c^		Breast cancer		Diabetes	
		AUC^d^	*F*_1_-score	AUC	*F*_1_-score	AUC	*F*_1_-score
OD^e^		84.81 (0.23)	72.74 (0.30)	69.37 (1.22)	60.01 (1.09)	62.13 (0.48)	47.73 (0.16)
**Balanced STD^f^**							
	CTGAN^g^, no condition	79.07 (1.12)	67.70 (1.54)	67.73 (3.24)	65.96 (4.37)	56.93 (0.31)	46.59 (0.16)
	CTGAN, CS^h^	79.01 (1.20)	68.47 (1.39)	54.88 (3.00)	52.59 (2.74)	57.23 (0.33)	46.38 (0.19)
	CTGAN, DC^i^	85.61 (0.29)^j^	75.09 (0.58)^j^	78.05 (1.59)^j^	71.03 (2.11)^j^	59.98 (0.24)^j^	53.47 (0.13)^j^
	CopulaGAN^k^, no condition	78.29 (0.74)	67.28 (1.67)	59.14 (2.32)	57.16 (3.46)	56.92 (0.26)	44.17 (0.12)
	CopulaGAN, CS	78.16 (1.72)	68.82 (1.82)	73.48 (4.73)	64.48 (8.02)	58.55 (0.24)	44.80 (0.13)
	CopulaGAN, DC	83.91 (0.35)	72.97 (0.67)	77.82 (1.83)	66.61 (4.66)	58.27 (0.31)	52.86 (0.26)
**Imbalanced STD**							
	CTGAN, no condition	67.20 (2.74)	41.94 (0.00)	53.29 (5.42)	38.75 (0.51)	53.18 (0.26)	47.09 (0.01)
	CTGAN, CS	68.03 (1.26)	41.94 (0.00)	54.48 (2.75)	38.75 (0.51)	54.41 (0.24)	47.09 (0.01)
	CTGAN, DC	77.98 (1.12)	47.05 (1.68)	59.81 (1.47)	39.84 (2.60)	56.44 (0.55)	49.42 (0.07)
	CopulaGAN, no condition	67.70 (2.00)	41.96 (0.11)	54.59 (1.20)	38.75 (0.51)	52.77 (0.35)	47.09 (0.01)
	CopulaGAN, CS	67.70 (1.84)	41.96 (0.11)	55.83 (2.32)	38.75 (0.51)	53.19 (0.76)	47.09 (0.01)
	CopulaGAN, DC	78.73 (1.57)	44.63 (0.88)	58.61 (1.96)	38.75 (0.51)	55.20 (0.23)	48.74 (0.68)

^a^RF: random forest.

^b^GAN: generative adversarial network.

^c^NSCLC: non–small cell lung cancer.

^d^AUC: area under the curve.

^e^OD: original data.

^f^STD: synthetic tabular data.

^g^CTGAN: conditional tabular generative adversarial network.

^h^CS: conditional sampling.

^i^DC: divide and conquer.

^j^The best results.

^k^CopulaGAN: copula generative adversarial network.

**Table 4:** Validation results obtained using the XGBoost^a^ classifier: mean (SD) values of 5 experiments.

Data type, GAN^b^, and approach		NSCLC^c^		Breast cancer		Diabetes	
		AUC^c^	*F*_1_-score	AUC	*F*_1_-score	AUC	*F*_1_-score
OD^d^		83.07 (0.37)	71.14 (1.09)	71.21 (0.46)	62.89 (2.59)	67.02 (0.13)	48.91 (0.18)
**Balanced STD^e^**							
	CTGAN^g^, no condition	76.50 (1.15)	66.48 (1.75)	68.77 (2.49)	68.38 (4.49)	57.95 (0.30)	47.85 (0.22)
	CTGAN, CS^h^	74.71 (1.59)	64.86 (1.75)	54.15 (2.49)	48.44 (3.25)	58.31 (0.20)	47.54 (0.15)
	CTGAN, DC^i^	85.20 (0.82)^j^	74.78 (0.77)^j^	77.86 (2.27)^j^	70.58 (3.36)^j^	60.18 (0.20)^j^	53.93 (0.29)^j^
	CopulaGAN^k^, no condition	77.18 (0.98)	67.56 (1.63)	56.47 (2.12)	54.75 (2.39)	59.25 (0.21)	46.51 (0.12)
	CopulaGAN, CS	76.42 (0.93)	67.82 (1.22)	68.32 (2.37)	60.65 (5.21)	58.98 (0.29)	46.55 (0.29)
	CopulaGAN, DC	83.58 (0.65)	72.92 (0.66)	77.69 (1.91)	64.96 (2.87)	58.84 (0.35)	53.10 (0.34)
**Imbalanced STD**							
	CTGAN, no condition	72.18 (4.12)	42.12 (0.42)	59.76 (1.72)	38.75 (0.51)	54.31 (0.30)	47.09 (0.01)
	CTGAN, CS	70.94 (2.93)	42.07 (0.32)	61.65 (1.49)	38.75 (0.51)	56.93 (0.37)	47.09 (0.01)
	CTGAN, DC	83.20 (0.42)	62.13 (2.43)	70.06 (1.07)	38.75 (0.51)	59.35 (0.39)	49.15 (0.29)
	CopulaGAN, no condition	72.40 (3.23)	42.59 (0.45)	65.77 (2.03)	38.75 (0.51)	55.86 (0.39)	47.10 (0.02)
	CopulaGAN, CS	73.21 (1.81)	42.75 (0.46)	57.42 (1.04)	38.75 (0.51)	54.63 (0.23)	47.09 (0.01)
	CopulaGAN, DC	82.60 (1.37)	59.22 (1.78)	68.19 (2.85)	38.75 (0.51)	57.95 (0.49)	48.26 (0.17)

^a^XGBoost: Extreme Gradient Boosting.

^b^GAN: generative adversarial network.

^c^NSCLC: non–small cell lung cancer.

^d^AUC: area under the curve.

^e^OD: original data.

^f^STD: synthetic tabular data.

^g^CTGAN: conditional tabular generative adversarial network.

^h^CS: conditional sampling.

^i^DC: divide and conquer.

^j^The best results.

^k^CopulaGAN: copula generative adversarial network.

**Table 5:** Validation results obtained using the LGBM^a^ classifier: mean (SD) values of 5 experiments.

Data type, GAN^b^, and approach		NSCLC^c^		Breast cancer		Diabetes	
		AUC^d^	*F*_1_-score	AUC	*F*_1_-score	AUC	*F*_1_-score
OD^e^		84.09 (0.03)	71.30 (0.72)	75.84 (1.80)	62.07 (3.05)	67.88 (0.12)	47.89 (0.16)
**Balanced STD^f^**							
	CTGAN^g^, no condition	77.31 (1.21)	66.90 (1.65)	66.52 (1.60)	65.32 (2.87)	58.42 (0.26)	48.38 (0.16)
	CTGAN, CS^h^	77.43 (1.78)	67.25 (2.09)	57.94 (3.23)	60.42 (3.45)	58.75 (0.17)	48.31 (0.07)
	CTGAN, DC^i^	85.14 (0.70)^j^	74.40 (0.78)^j^	78.16 (1.52)^j^	71.75 (1.79)^j^	61.75 (0.13)^j^	54.09 (0.19)^j^
	CopulaGAN^k^, no condition	77.61 (0.86)	68.50 (1.21)	59.38 (1.15)	56.21 (3.63)	60.36 (0.27)	46.68 (0.12)
	CopulaGAN, CS	77.62 (1.85)	68.58 (1.14)	70.02 (2.17)	65.51 (2.97)	61.12 (0.23)	46.75 (0.12)
	CopulaGAN, DC	83.57 (0.55)	72.84 (0.59)	75.31 (2.45)	68.13 (1.72)	60.03 (0.23)	53.63 (0.16)
**Imbalanced STD**							
	CTGAN, no condition	71.84 (3.03)	42.03 (0.20)	59.81 (0.66)	38.75 (0.51)	55.79 (0.25)	47.09 (0.01)
	CTGAN, CS	71.51 (2.65)	41.96 (0.11)	64.99 (1.48)	38.75 (0.51)	58.47 (0.24)	47.10 (0.01)
	CTGAN, DC	83.60 (1.00)	61.55 (2.68)	70.54 (0.56)	43.00 (0.68)	60.80 (0.21)	49.71 (0.06)
	CopulaGAN, no condition	73.83 (3.15)	42.27 (0.40)	63.92 (1.67)	38.75 (0.51)	56.45 (0.34)	47.10 (0.03)
	CopulaGAN, CS	74.21 (2.37)	42.42 (0.53)	60.28 (0.73)	38.75 (0.51)	56.31 (0.29)	47.11 (0.03)
	CopulaGAN, DC	81.92 (1.10)	57.91 (2.42)	72.84 (1.40)	40.83 (2.71)	58.34 (0.35)	48.53 (0.11)

^a^LGBM: light gradient-boosting machine.

^b^GAN: generative adversarial network.

^c^NSCLC: non–small cell lung cancer.

^d^AUC: area under the curve.

^e^OD: original data.

^f^STD: synthetic tabular data.

^g^CTGAN: conditional tabular generative adversarial network.

^h^CS: conditional sampling.

^i^DC: divide and conquer.

^j^The best results.

^k^CopulaGAN: copula generative adversarial network.

**Table 6:** Summary of quality tests for the NSCLC^a^ data set: mean (SD) values of 5 experiments.

Approach and GAN^b^			Shape		Pair trend		Overall
**CS^c^**							
	CTGAN^d^	88.42 (0.12)		83.47 (0.10)		85.95 (0.11)	
	CopulaGAN^e^	89.98 (0.06)		79.77 (3.84)		84.87 (1.93)	
**DC^f^**							
	CTGAN	90.49 (0.07)^g^		83.92 (0.10)^g^		87.20 (0.08)^g^	
	CopulaGAN	89.72 (0.07)		82.48 (0.12)		86.10 (0.09)	

^a^NSCLC: non–small cell lung cancer.

^b^GAN: generative adversarial network.

^c^CS: conditional sampling.

^d^CTGAN: conditional tabular generative adversarial network.

^e^CopulaGAN: copula generative adversarial network.

^f^DC: divide and conquer.

^g^The best results.

**Table 7:** Summary of quality tests for the breast cancer data set: mean (SD) values of 5 experiments.

Approach and GAN^a^			Shape		Pair trend		Overall
**CS^b^**							
	CTGAN^c^	90.75 (0.14)		80.97 (0.17)		85.86 (0.15)	
	CopulaGAN^d^	89.25 (0.18)		80.68 (0.16)		84.97 (0.21)	
**DC^e^**							
	CTGAN	91.71 (0.12)^f^		82.72 (0.13)^f^		87.21 (0.09)^f^	
	CopulaGAN	91.18 (0.11)		81.24 (0.14)		86.21 (0.14)	

^a^GAN: generative adversarial network.

^b^CS: conditional sampling.

^c^CTGAN: conditional tabular generative adversarial network.

^d^CopulaGAN: copula generative adversarial network.

^e^DC: divide and conquer.

^f^The best results.

**Table 8:** Summary of quality tests for the diabetes data set: mean (SD) values of 5 experiments.

Approach and GAN^a^			Shape		Pair trend		Overall
**CS^b^**							
	CTGAN^c^	97.55 (0.23)		95.27 (0.32)		96.41 (0.26)	
	CopulaGAN^d^	97.22 (0.24)		94.27 (0.32)		95.74 (0.36)	
**DC^e^**							
	CTGAN	98.60 (0.31)^f^		96.70 (0.26)^f^		97.65 (0.27)^f^	
	CopulaGAN	97.90 (0.27)		95.74 (0.23)		96.82 (0.31)	

^a^GAN: generative adversarial network.

^b^CS: conditional sampling.

^c^CTGAN: conditional tabular generative adversarial network.

^d^CopulaGAN: copula generative adversarial network.

^e^DC: divide and conquer.

^f^The best results.

## Discussion

### Principal Findings

Preserving data with logical relationships while generating STD using GANs has not been sufficiently researched. Some GANs, such as the CTGAN and CopulaGAN, use CS filtering to determine the exclusion of record data based on predefined condition columns after generating STD. However, this is highly dependent on condition columns, which may lead to meaningful information in the excluded records being ignored. To resolve this problem, we proposed a DC-based approach in this paper, as shown in Multimedia Appendix 4.

The proposed DC-based approach was verified to produce STD involving logical relationships between columns. As the division strategy, we used class-specific and the Cramer V criteria sequentially. First, we used a class-specific criterion to classify dependent classes between survival and death groups. Subsequently, we measured the relative degrees of association among pairs of variables based on the Cramer V correlation coefficient in order to identify strong evidence for meaningful correlations between columns. In terms of a high Cramer V correlation coefficient (=1), smoker and nonsmoker groups were selected as division criteria. Using this, the OD was divided into smaller data sets comprising hierarchical group data that considered class-specific aspects of learning. Further, the division criteria of the DC strategy avoided the problem of ignoring some records owing to overreliance on condition columns.

To compare the logical STD generation approaches, we trained the CTGAN and CopulaGAN with CS filtering and compared their performances with those of ML models trained using a DC approach without CS filtering. The results demonstrated that the epochs hyperparameter was sensitive, with a significant impact on the quality of synthetic data generated using the CTGAN and CopulaGAN. Specifically, the results depended considerably on the value of the epochs hyperparameter, ranging from 100 to 500. We used a grid search algorithm to identify an optimal value for the epochs hyperparameter, as shown in Multimedia Appendix 5. Regularization hyperparameters, such as grid search, are essential to the generalization of ML models [34]. They work well with low-dimensional hyperparameter spaces and ample computational resources [35]. A grid search involves testing a range of hyperparameter values and evaluating the performance of the model corresponding to each value. In our case, we tested epoch values of 100, 200, 300, 400, and 500 and evaluated the resulting synthetic data using a variety of metrics, including distributional similarity, feature correlation, and downstream performance in predictive models. Our findings highlight the importance of carefully selecting hyperparameters during GAN training to generate synthetic data from clinical data sets. The sensitivity of the epochs hyperparameter underscores the necessity of systematic approaches, such as grid search, to identify optimal values.

Generally, ML training on imbalanced data sets leads to failure to properly learn the distributive characteristics of the data and, consequently, unfavorable accuracies across the data classes [36]. We generated balanced and imbalanced STD by regulating the volumes of the dependence variables for comparison. These data were used to develop ML models (DT, RF, XGBoost, and LGBM), and their AUC and *F*_1_-score were measured on the verification data set. The hyperparameter of each model was tuned via a grid search for the number of epochs. All balanced synthetic data exhibited higher performance on the prediction models (DT, RF, XGBoost, and LGBM) compared to imbalanced synthetic data. Therefore, we recommend that the volume of balanced dependence variables be considered during SDG using GANs.

Finally, the DC-based approach was observed to exhibit several potential advantages over CS. First, deconstruction of the division criteria into simpler subrules enables the specification of complex or multidimensional conditions. Second, training the GAN on each subrule independently reduces the risk of information loss by CS, as the GAN can focus on generating synthetic data that accurately reflect the distribution of the data for each subrule. Finally, combining the results of the subrules enables the generation of synthetic data that satisfy all the original logical rules, without requiring complex and potentially overspecified conditions.

Thus, the main contribution of this paper is to demonstrate the viability of the proposed STD generation method to serve as a revolutionary new alternative to existing counterparts in the development of ML-based prediction models.

### Limitations

Our study is limited in terms of the low dimensionality and count of data collected from a single country. In practical health care, low-dimensional and sparse data are often derived from strict data preprocessing, a detailed design for the target population, or exact primary endpoints. In this paper, data containing essential variables were collected from 13 regional cancer centers and 39 hospitals via sampling. However, patients with NSCLC from only a single country were considered, potentially introducing racial bias. We intend to overcome this limitation in future works by applying the proposed framework to data collected from other countries.

The DC-based STD learning strategy may be difficult to apply in the case of sparse data and multiple division criteria. Indiscriminate use of the strategy, even in the presence of a large amount of data, can be problematic because the use of multiple division strategies induces a lack of learning data, which motivates the generation of inappropriate synthetic data. Therefore, it is important to establish appropriate criteria for the division strategy (eg, the class-specific and Cramer V criteria proposed in our study). We recommend that the class-specific criterion be used as an essential strategy in the first division criteria. The Cramer V criterion should be used to calculate correlations between variables, enabling sufficient discussion about the group of candidates for division and helping decide the need for division.

One potential challenge with the DC approach is that the subrules and the combinations of results require careful consideration. If the subrules are not well defined or the combinations of results are not appropriate, the resulting synthetic data may not accurately reflect the characteristics of real-world data. Additionally, if data with logical relationships are highly interdependent, it may be challenging to break them down into independent subrules. Despite these potential challenges, the DC approach exhibited great promise in generating synthetic data from data with logical relationships on clinical data sets.

### Conclusion

Our study demonstrated problems of CS-based STD generation techniques and the feasibility of DC-based STD generation to address those problems. Further, the effectiveness of the generated STD to enable the application of ML models was verified, revealing that they improve prediction performance.

## Appendix

Multimedia Appendix 1 Data set characteristics.

Multimedia Appendix 2 Cramer V correlation.

Multimedia Appendix 3 Distribution plots.

Multimedia Appendix 4 As-is and to-be.

Multimedia Appendix 5 Effect of epoch on validation results.

## Abbreviations

A1C: glycated hemoglobin
ALK FISH: anaplastic lymphoma kinase fluorescence in situ hybridization
ALK IHC: anaplastic lymphoma kinase immunohistochemistry
AUC: area under curve
CDF: cumulative distribution function
CS: conditional sampling
CTGAN: conditional tabular generative adversarial network
CopulaGAN: copula generative adversarial network
DC: divide and conquer
DLCO: diffusing capacity of the lungs for carbon monoxide
DT: decision tree
ECOG: Eastern Cooperative Oncology Group
EGFR: epidermal growth factor receptor
FEV1: forced expiratory volume in 1 second
FPR: false-positive rate
FVC: forced vital capacity
GAN: generative adversarial network
KALC-R: Korea Association for Lung Cancer Registry
KSS: Kolmogorov-Smirnov statistic
LGBM: light gradient-boosting machine
ML: machine learning
NSCLC: non–small cell lung cancer
OD: original data
OOB: out-of-bag
RF: random forest
ROC: receiver operating characteristic
RT: radiotherapy
SDG: synthetic data generation
STD: synthetic tabular data
TPR: true-positive rate
TVD: total variation distance
XGBoost: Extreme Gradient Boosting

## Data Availability

Anyone can use the original data after registering as a member on the Korea Central Cancer Registry (KCCR) portal [37] and passing through the data application and review. Users need to fill out an application form, including a research proposal describing how they will use the data and that the data access request will be accessed by the KCCR and the National Statistics Office. All synthetic data can be shared for research purposes by contacting the authors.

All code for data generation and validation associated with the current submission is available in a GitHub repository [38].
