# Supplementary material for: Synthetic Tabular Data Based on Generative Adversarial Networks in Health Care: Generation and Validation Using the Divide-and-Conquer Strategy
Source: JMIR Med Inform. 2023 Nov 24;11:e47859. doi: 10.2196/47859 (PMC10709788; doi:10.2196/47859)
Supplement: Multimedia Appendix 1 [file medinform_v11i1e47859_app1.docx]

**Multimedia Appendix 1**

Tables A1-1, A1-2, and A1-3 present the basic characteristics of data related to lung cancer, breast cancer, and diabetes patients, respectively. Data is presented as percentages or mean ± SD.

Table A1-1. Comparison of basic characteristics of development data and validation data for lung cancer.

| Variable | Development data  N = 1,616 | Validation data  N = 688 |
| --- | --- | --- |
| Age | 66.08±10.247 | 65.69±10.613 |
| Height | 162.219±8.492 | 162.122±8.279 |
| Weight | 61.188±10.445 | 61.471±10.712 |
| FVC | 3.163±0.870 | 3.148±0.844 |
| FEV1 | 2.242±0.699 | 2.251±0.668 |
| DLCO | 15.436±5.720 | 15.688±5.535 |
| DLCO percent | 82.914±23.365 | 23.558±22.845 |
| Smoker |  |  |
| Non-smoker | 37.69 % (609) | 41.72% (287) |
| Past-smoker | 31.99 % (517) | 33.43% (230) |
| Current-smoker | 30.32 % (490) | 24.86% (171) |
| Pack-Year | 23.350±25.672 | 23.060±27.640 |
| Male | 67.45 % (1090) | 64.97% (447) |
| ECOG |  |  |
| 0 | 42.33% (684) | 42.15% (290) |
| 1 | 30.45% (492) | 32.12% (221) |
| 2 | 4.89% (79) | 3.63% (25) |
| 3 | 1.30% (21) | 1.74% (12) |
| 4 | 0.19% (3) | 0.44% (3) |
| 5 | 0.19% (3) | 0.29% (2) |
| 9 (unknown) | 20.67% (334) | 19.62% (135) |
| NSCLC |  |  |
| Adenocarcinoma | 68.07% (1100) | 69.48% (478) |
| Squamous cell carcinoma | 31.13% (503) | 30.09% (207) |
| Large cell carcinoma | 1.18% (19) | 0.73% (5) |
| Positive EGFR mutation | 23.39% (378) | 23.26% (160) |
| Positive ALK IHC | 3.22 % (52) | 3.05% (21) |
| Positive ALK FISH | 1.55% (25) | 2.03% (14) |
| STAGE |  |  |
| 1 | 39.05% (631) | 38.66% (266) |
| 2 | 10.21% (165) | 10.90% (75) |
| 3 | 20.67% (334) | 19.19% (132) |
| 4 | 30.07% (486) | 31.25% (215) |
| OP Curative | 51.91% (839) | 50.73% (349) |
| Radiotherapy | 31.62% (511) | 28.20% (194) |
| Curative chemotherapy | 19.56% (316) | 17.30% (119) |
| Palliative chemotherapy | 27.04% (437) | 29.22% (201) |
| All cause of death | 27.79% (449) | 27.76% (191) |

Data are expressed as the number of patients (percentage) or mean ± SD. FVC: forced vital capacity; FEV1: forced expiratory volume in one second; DLCO: diffusing capacity for carbon monoxide; ECOG: eastern cooperative oncology group; NSCLC: non-small cell lung cancer; EGFR: epidermal growth factor receptor; ALK IHC: anaplastic lymphoma kinase immunohistochemistry; ALK FISH: anaplastic lymphoma kinase fluorescence in situ hybridization; OP: operation.

Table A1-2. Comparison of basic characteristics of development data and validation data for breast cancer.

| Variable | Development data  N = 228 | Validation data  N = 58 |
| --- | --- | --- |
| Age |  |  |
| 20-29 | - | 1.72% (1) |
| 30-39 | 11.84% (27) | 15.52% (9) |
| 40-49 | 32.89% (75) | 25.86% (15) |
| 50-59 | 34.65% (79) | 29.31% (17) |
| 60-69 | 18.42% (42) | 25.86% (15) |
| 70-79 | 2.19% (5) | 1.72% (1) |
| Menopause |  |  |
| Premeno | 51.32% (117) | 56.9% (33) |
| GE40 | 45.61% (104) | 43.1% (25) |
| LT40 | 3.07% (7) | - |
| Tumor size |  |  |
| 0-4 | 3.07% (7) | 1.72% (1) |
| 5-9 | 1.32% (3) | 1.72% (1) |
| 10-14 | 9.65% (22) | 10.34% (6) |
| 15-19 | 10.53% (24) | 10.34% (6) |
| 20-24 | 17.11% (39) | 18.97% (11) |
| 25-29 | 17.98% (41) | 22.41% (13) |
| 30-34 | 22.37% (51) | 15.52% (9) |
| 35-39 | 5.7% (13) | 10.34% (6) |
| 40-44 | 8.77% (20) | 3.45% (2) |
| 45-49 | 0.88% (2) | 1.72% (1) |
| 50-54 | 2.63% (6) | 3.45% (2) |
| Inv codes |  |  |
| 0-2 | 73.68% (168) | 77.59% (45) |
| 3-5 | 12.28% (28) | 13.79% (8) |
| 6-8 | 7.02% (16) | 1.72% (1) |
| 9-11 | 3.07% (7) | 5.17% (3) |
| 12-14 | 1.32% (3) | - |
| 15-17 | 2.63% (6) | - |
| 24-26 | - | 1.72% (1) |
| Node caps | 21.05% (48) | 13.79% (8) |
| Degree of malignancy |  |  |
| 1 | 24.12% (55) | 27.59% (16) |
| 2 | 43.42% (99) | 53.45% (31) |
| 3 | 32.46% (74) | 18.97% (11) |
| Breast |  |  |
| Left | 55.26% (126) | 44.83% (26) |
| Right | 44.74% (102) | 55.17% (32) |
| Breast quadrant |  |  |
| Left low | 38.16% (87) | 39.66% (23) |
| Left up | 34.65% (79) | 31.03% (18) |
| Right low | 9.21% (21) | 5.17% (3) |
| Right up | 11.84% (27) | 10.34% (6) |
| Central | 5.7% (13) | 13.79% (8) |
| Irradiation | 22.37% (51) | 29.31% (17) |
| Recurrence event | 28.07% (64) | 36.21% (21) |

Table A1-3. Comparison of basic characteristics of development data and validation data for diabetes cancer.

| Variable | Development data  N = 81,410 | Validation data  N = 20,353 |
| --- | --- | --- |
| Time in hospital | 4.395±2.984 | 4.4±2.99 |
| Number of lab procedures | 43.078±19.657 | 43.166±19.743 |
| Number of procedures | 1.341±1.706 | 1.334±1.706 |
| Number of medications | 16.021±8.114 | 16.024±8.183 |
| Number of outpatient | 0.369±1.272 | 0.37±1.247 |
| Number of emergency | 0.196±0.92 | 0.206±0.972 |
| Number of inpatient | 0.636±1.262 | 0.635±1.266 |
| Number of diagnoses | 7.424±1.935 | 7.419±1.928 |
| Race |  |  |
| African American | 18.88% (15,370) | 18.87% (3,840) |
| Caucasian | 74.8% (60,894) | 74.71% (15,205) |
| Other | 6.32% (5,146) | 6.43% (1,308) |
| Gender |  |  |
| Male | 46.33% (37,718) | 45.88% (9,337) |
| Female | 53.67% (43,692) | 54.12% (11,016) |
| Age |  |  |
| 40-49 | 9.51% (7,744) | 9.54% (1,941) |
| 50-59 | 17.02% (13,860) | 16.69% (3,396) |
| 60-69 | 22.05% (17,951) | 22.26% (4,531) |
| 70-79 | 25.67% (20,896) | 25.4% (5,170) |
| 80-89 | 16.82% (13,695) | 17.21% (3,502) |
| Medical specialty |  |  |
| Cardiology | 5.24% (4,268) | 5.32% (1,083) |
| Emergency/Trauma | 7.4% (6,023) | 7.58% (1,542) |
| Family/General Practice | 7.28% (5,930) | 7.42% (1,510) |
| Internal Medicine | 14.52% (11,821) | 13.83% (2,814) |
| Other | 65.55% (53,366) | 65.85% (13,403) |
| A1C result |  |  |
| Greater than 8 | 8.12% (6,611) | 7.89% (1,605) |
| None | 83.26% (67,781) | 83.35% (16,964) |
| Other | 8.62% (7,018) | 8.77% (1,784) |
| Metformin |  |  |
| No | 80.23% (65,314) | 80.88% (16,462) |
| Other | 19.77% (16,096) | 19.12% (3,891) |
| Glipizide | 87.65% (71,353) | 87.09% (17,725) |
| Glyburide | 89.56% (72,909) | 89.44% (18,204) |
| Insulin | 46.49% (37,851) | 46.82% (9,529) |
| Change | 46.28% (37,676) | 45.86% (9,333) |
| Diabetes Medication | 77.02% (62,703) | 76.93% (15,658) |
| Diagnosis 1 |  |  |
| Circulatory | 29.93% (24,369) | 29.91% (6,088) |
| Diabetes | 8.6% (6,999) | 8.64% (1,758) |
| Genitourinary | 4.96% (4,036) | 5.31% (1,081) |
| Respiratory | 14.18% (11,542) | 14.16% (2,881) |
| Other | 42.33% (34,464) | 41.98% (8,545) |
| Diagnosis 2 |  |  |
| Circulatory | 31.68% (25,791) | 31.68% (6,447) |
| Diabetes | 12.59% (10,246) | 12.52% (2,548) |
| Genitourinary | 8.21% (6,685) | 8.31% (1,691) |
| Respiratory | 10.66% (8,680) | 10.88% (2,215) |
| Other | 36.86% (30,008) | 36.61% (7,452) |
| Diagnosis 3 |  |  |
| Circulatory | 31.21% (25,406) | 31.06% (6,322) |
| Diabetes | 16.75% (13,638) | 17.29% (3,519) |
| Genitourinary | 6.59% (5,365) | 6.46% (1,315) |
| Respiratory | 7.24% (5,894) | 7.19% (1,464) |
| Other | 38.21% (31,107) | 37.99% (7,733) |
| Readmitted | 11.19% (9,112) | 11.03% (2,245) |

In the development data, we compared the basic characteristics of different groups for lung cancer survival, breast cancer recurrence, and diabetes readmission, as shown in Tables A1-4, A1-5, and A1-6, respectively.

Table A1-4. Comparison of basic characteristics of survival group and non-survival group in development data for lung cancer data.

| Variable | Development data | |
| --- | --- | --- |
|  | Survival Group  N = 1,167 | Non-survival Group  N = 449 |
| Age | 64.68±10.25 | 69.73±9.30 |
| Height | 161.87±8.68 | 163.12±7.91 |
| Weight | 61.90±10.38 | 59.35±10.41 |
| FVC | 3.27±0.69 | 2.88±0.80 |
| FEV1 | 2.36±0.69 | 1.95±0.63 |
| DLCO | 16.53±5.56 | 12.61±5.12 |
| DLCO percent | 87.30±22.18 | 71.52±22.52 |
| Smoker |  |  |
| Non-smoker | 43.10% (503) | 23.61% (106) |
| Past-smoker | 28.57% (334) | 40.76% (183) |
| Current-smoker | 28.28% (330) | 35.64% (160) |
| Pack-Year | 20.60±25.19 | 30.50±25.57 |
| Male | 61.44% (717) | 83.07% (373) |
| ECOG |  |  |
| 0 | 50.39% (588) | 21.38% (96) |
| 1 | 26.22% (306) | 41.43% (186) |
| 2 | 3.09% (36) | 9.58% (43) |
| 3 | 0.69% (8) | 2.90% (13) |
| 4 | 0.17% (2) | 0.22% (1) |
| 5 | 0% (0) | 0.67% (3) |
| 9 (unknown) | 19.45% (227) | 23.83% (107) |
| NSCLC |  |  |
| Adenocarcinoma | 73.95% (863) | 52.78% (237) |
| Squamous cell carcinoma | 25.36% (296) | 46.10% (207) |
| Large cell carcinoma | 1.11% (13) | 1.34% (6) |
| Positive EGFR mutation | 28.02% (327) | 11.36% (51) |
| Positive ALK IHC | 3.51% (41) | 2.45% (11) |
| Positive ALK FISH | 1.71% (20) | 1.11% (5) |
| STAGE |  |  |
| 1 | 50.90% (594) | 8.24% (37) |
| 2 | 11.91% (139) | 5.79% (26) |
| 3 | 18.34% (214) | 26.73% (120) |
| 4 | 18.85% (220) | 59.24% (266) |
| OP Curative | 64.78% (756) | 11.80% (53) |
| Radiotherapy | 28.28% (330) | 4.31% (181) |
| Curative chemotherapy | 22.54% (263) | 11.80% (53) |
| Palliative chemotherapy | 22.37% (261) | 39.20% (176) |

Data are expressed as the number of patients (percentage) or mean ± SD. FVC: forced vital capacity; FEV1: forced expiratory volume in one second; DLCO: diffusing capacity for carbon monoxide; ECOG: eastern cooperative oncology group; NSCLC: non-small cell lung cancer; EGFR: epidermal growth factor receptor; ALK IHC: anaplastic lymphoma kinase immunohistochemistry; ALK FISH: anaplastic lymphoma kinase fluorescence in situ hybridization; OP: operation.

Table A1-5. Comparison of basic characteristics of recurrence group and non-recurrence group in development data for breast cancer data.

| Variable | Development data | |
| --- | --- | --- |
|  | Recurrence Group  N = 64 | Non-recurrence Group  N = 164 |
| Age |  |  |
| 30-39 | 17.19% (11) | 9.76% (16) |
| 40-49 | 32.81% (21) | 32.93% (54) |
| 50-59 | 31.25% (20) | 35.98% (59) |
| 60-69 | 18.75% (12) | 18.29% (30) |
| 70-79 | - | 3.05% (5) |
| Menopause |  |  |
| Premeno | 54.69% (35) | 50.0% (82) |
| GE40 | 42.19% (27) | 46.95% (77) |
| LT40 | 3.12% (2) | 3.05% (5) |
| Tumor size |  |  |
| 0-4 | 1.56% (1) | 3.66% (6) |
| 5-9 | - | 1.83% (3) |
| 10-14 | 1.56% (1) | 12.8% (21) |
| 15-19 | 7.81% (5) | 11.59% (19) |
| 20-24 | 17.19% (11) | 17.07% (28) |
| 25-29 | 20.31% (13) | 17.07% (28) |
| 30-34 | 31.25% (20) | 18.9% (31) |
| 35-39 | 6.25% (4) | 5.49% (9) |
| 40-44 | 9.38% (6) | 8.54% (14) |
| 45-49 | 1.56% (1) | 0.61% (1) |
| 50-54 | 3.12% (2) | 2.44% (4) |
| Inv codes |  |  |
| 0-2 | 50.0% (32) | 82.93% (136) |
| 3-5 | 21.88% (14) | 8.54% (14) |
| 6-8 | 15.62% (10) | 3.66% (6) |
| 9-11 | 4.69% (3) | 2.44% (4) |
| 12-14 | 3.12% (2) | 0.61% (1) |
| 15-17 | 4.69% (3) | 1.83% (3) |
| Node caps | 40.62% (26) | 13.41% (22) |
| Degree of malignancy |  |  |
| 1 | 14.06% (9) | 28.05% (46) |
| 2 | 28.12% (18) | 49.39% (81) |
| 3 | 57.81% (37) | 22.56% (37) |
| Breast |  |  |
| Left | 56.25% (36) | 54.88% (90) |
| Right | 43.75% (28) | 45.12% (74) |
| Breast quadrant |  |  |
| Left low | 40.62% (26) | 37.2% (61) |
| Left up | 31.25% (20) | 35.98% (59) |
| Right low | 9.38% (6) | 9.15% (15) |
| Right up | 14.06% (9) | 10.98% (18) |
| Central | 3.12% (2) | 6.71% (11) |
| Irradiation | 35.94% (23) | 17.07% (28) |

Table A1-6. Comparison of basic characteristics of readmitted and non-readmitted group in development data for diabetes data.

| Variable | Development data | |
| --- | --- | --- |
|  | Readmitted Group  N = 9,112 | Non-readmitted Group  N = 72,298 |
| Time in hospital | 4.754±3.03 | 4.35±2.975 |
| Number of lab procedures | 44.188±19.267 | 42.938±19.701 |
| Number of procedures | 1.28±1.636 | 1.349±1.714 |
| Number of medications | 16.852±8.008 | 15.917±8.121 |
| Number of outpatient | 0.443±1.334 | 0.36±1.264 |
| Number of emergency | 0.35±1.379 | 0.176±0.842 |
| Number of inpatient | 1.226±1.941 | 0.561±1.127 |
| Number of diagnoses | 7.691±1.777 | 7.39±1.951 |
| Race |  |  |
| African American | 19.13% (1,743) | 18.85% (13,627) |
| Caucasian | 75.47% (6,877) | 74.71% (54,017) |
| Other | 5.4% (492) | 6.44% (4,654) |
| Gender |  |  |
| Male | 45.79% (4,172) | 46.4% (33,546) |
| Female | 54.21% (4,940) | 53.6% (38,752) |
| Age |  |  |
| 40-49 | 9.1% (829) | 9.56% (6,915) |
| 50-59 | 14.83% (1,351) | 17.3% (12,509) |
| 60-69 | 21.94% (1,999) | 22.06% (15,952) |
| 70-79 | 27.07% (2,467) | 25.49% (18,429) |
| 80-89 | 18.29% (1,667) | 16.64% (12,028) |
| Medical specialty |  |  |
| Cardiology | 3.63% (331) | 5.45% (3,937) |
| Emergency/Trauma | 7.16% (652) | 7.43% (5,371) |
| Family/General Practice | 7.96% (725) | 7.2% (5,205) |
| Internal Medicine | 14.41% (1,313) | 14.53% (10,508) |
| Other | 66.85% (6,091) | 65.39% (47,275) |
| A1C result |  |  |
| Greater than 8 | 7.2% (656) | 8.24% (5,955) |
| None | 85.13% (7,757) | 83.02% (60,024) |
| Other | 7.67% (699) | 8.74% (6,319) |
| Metformin | 82.88% (7,552) | 79.89% (57,762) |
| Glipizide | 87.16% (7,942) | 87.71% (63,411) |
| Glyburide | 89.91% (8,193) | 89.51% (64,716) |
| Insulin | 41.73% (3,802) | 47.1% (34,049) |
| Change | 49.22% (4,485) | 45.91% (33,191) |
| Diabetes Medication | 80.36% (7,322) | 76.6% (55,381) |
| Diagnosis 1 |  |  |
| Circulatory | 30.66% (2,794) | 29.84% (21,575) |
| Diabetes | 9.88% (900) | 8.44% (6,099) |
| Genitourinary | 4.95% (451) | 4.96% (3,585) |
| Respiratory | 41.91% (3,819) | 42.39% (30,645) |
| Other | 12.6% (1,148) | 14.38% (10,394) |
| Diagnosis 2 |  |  |
| Circulatory | 31.18% (2,841) | 31.74% (22,950) |
| Diabetes | 11.98% (1,092) | 12.66% (9,154) |
| Genitourinary | 8.75% (797) | 8.14% (5,888) |
| Respiratory | 37.92% (3,455) | 36.73% (26,553) |
| Other | 10.17% (927) | 10.72% (7,753) |
| Diagnosis 3 |  |  |
| Circulatory | 29.62% (2,699) | 31.41% (22,707) |
| Diabetes | 15.73% (1,433) | 16.88% (12,205) |
| Genitourinary | 8.18% (745) | 6.39% (4,620) |
| Respiratory | 38.51% (3,509) | 38.17% (27,598) |
| Other | 7.97% (726) | 7.15% (5,168) |
