# Supplementary material for: Synthetic Tabular Data Based on Generative Adversarial Networks in Health Care: Generation and Validation Using the Divide-and-Conquer Strategy
Source: JMIR Med Inform. 2023 Nov 24;11:e47859. doi: 10.2196/47859 (PMC10709788; doi:10.2196/47859)
Supplement: Multimedia Appendix 2 [file medinform_v11i1e47859_app2.docx]

**Multimedia Appendix 2**

Figure A2-2 and Figure A2-2 present the Cramer’s V correlation of breast cancer and diabetes data, respectively.


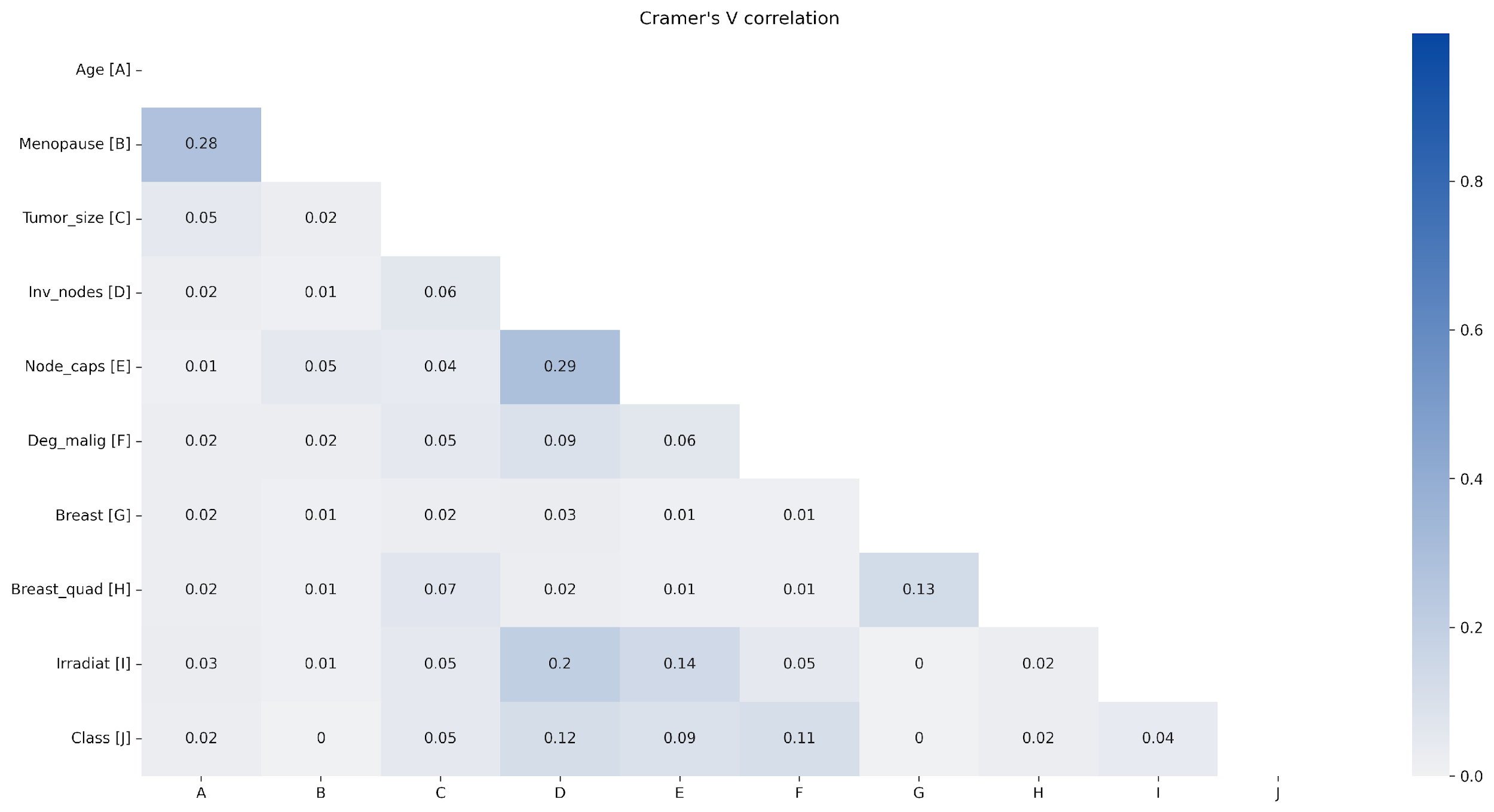
Figure A2-1. Cramer’s V correlation of breast cancer data.


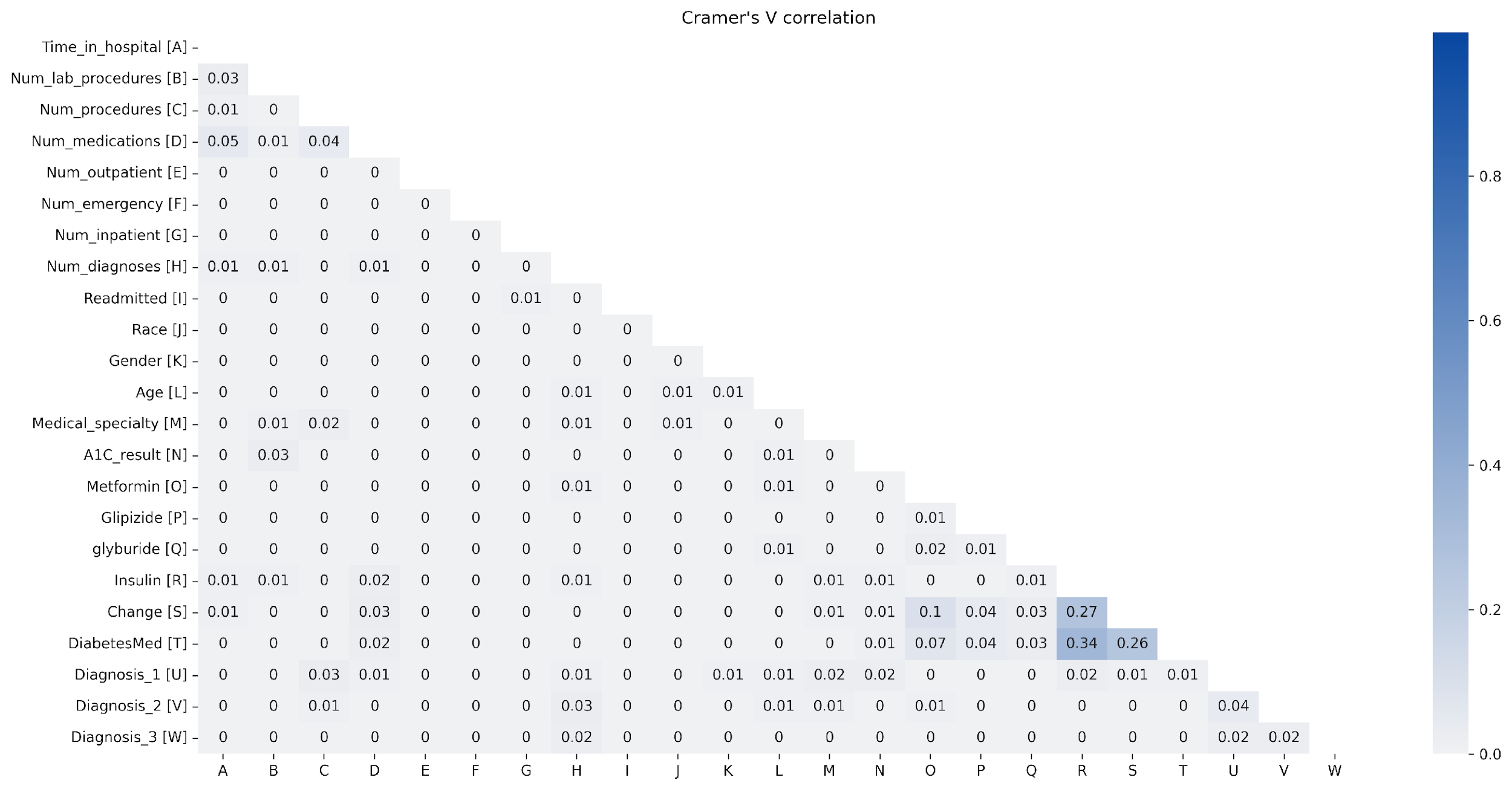
Figure A2-2. Cramer’s V correlation of diabetes data.
