# Supplementary material for: Synthetic Tabular Data Based on Generative Adversarial Networks in Health Care: Generation and Validation Using the Divide-and-Conquer Strategy
Source: JMIR Med Inform. 2023 Nov 24;11:e47859. doi: 10.2196/47859 (PMC10709788; doi:10.2196/47859)
Supplement: Multimedia Appendix 3 [file medinform_v11i1e47859_app3.docx]

**Multimedia Appendix 3**

Figures A3-[1-87] illustrate a feature-wise comparison between synthetic and real data, providing insights into the quality of the synthetic data.


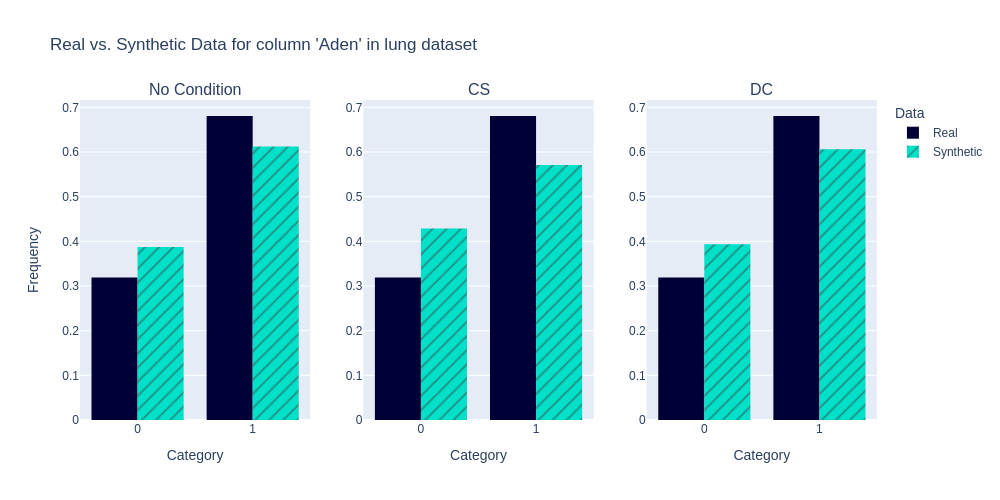


Figure A3-1. Data quality of “aden” column in the lung cancer data.


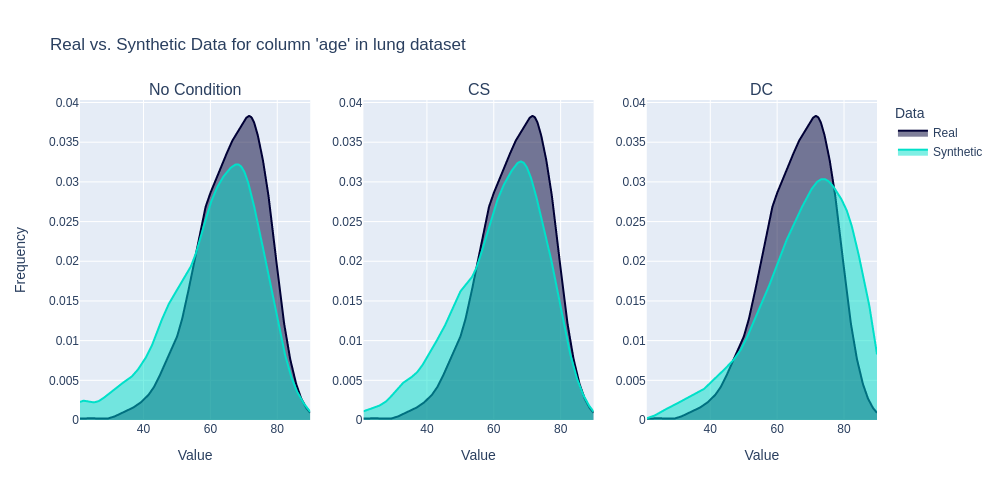


Figure A3-2. Data quality of “age” column in the lung cancer data.


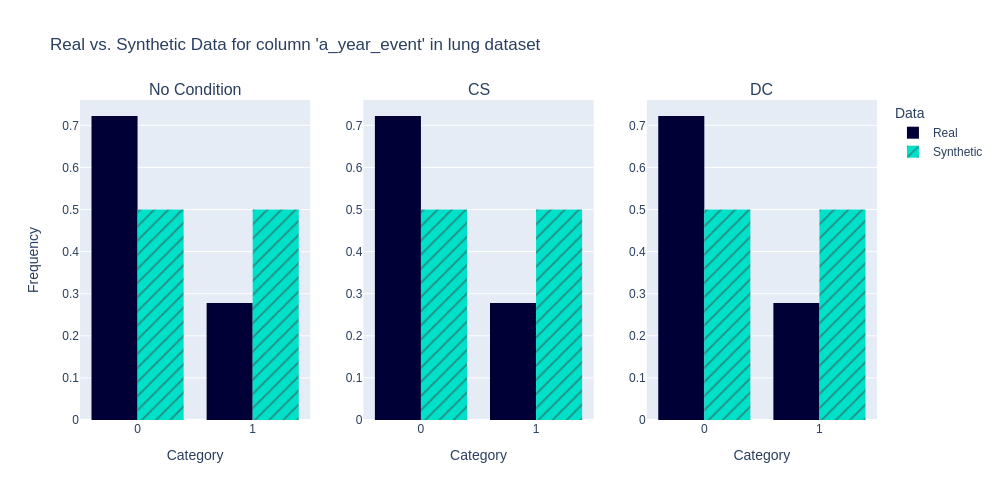


Figure A3-3. Data quality of “a year event” column in the lung cancer data.


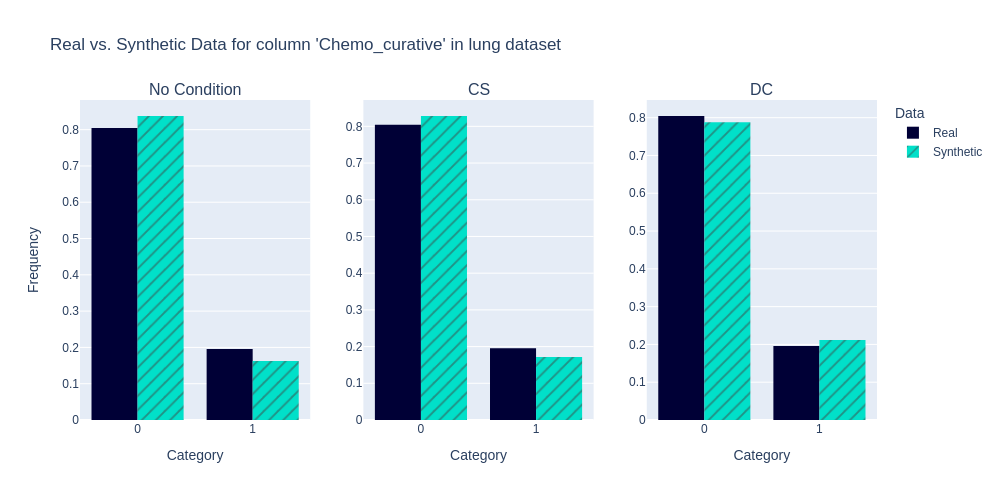


Figure A3-4. Data quality of “chemo curative” column in the lung cancer data.


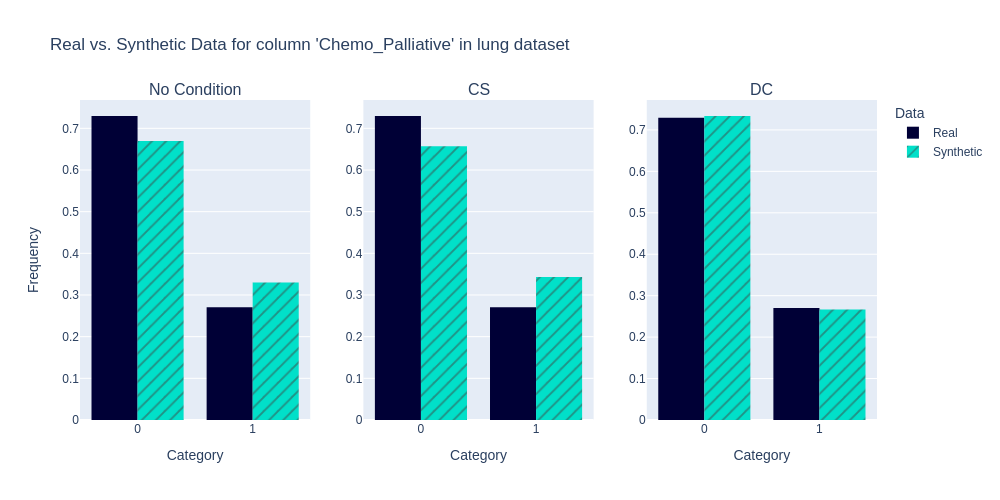


Figure A3-5. Data quality of “chemo palliative” column in the lung cancer data.


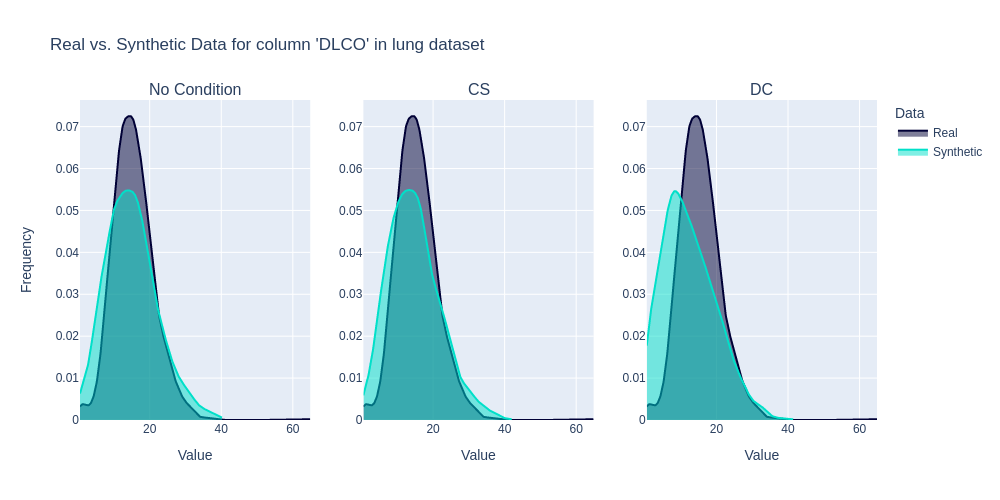


Figure A3-6. Data quality of “DLCO” column in the lung cancer data.


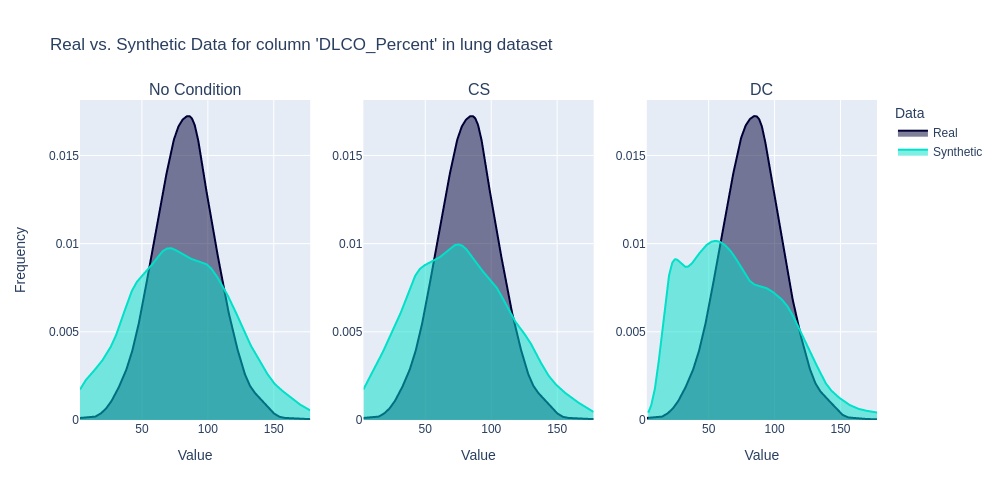


Figure A3-7. Data quality of “DLCO percent” column in the lung cancer data.


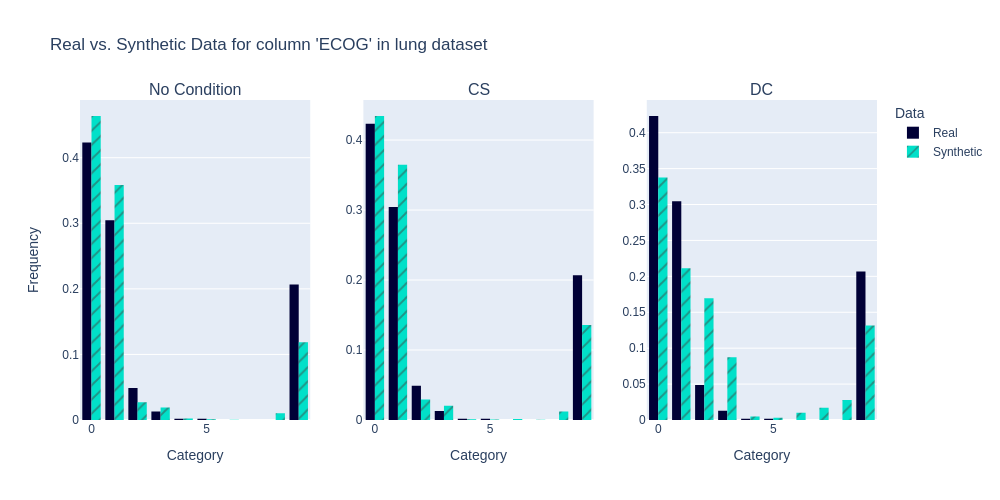


Figure A3-8. Data quality of “ECOG” column in the lung cancer data.


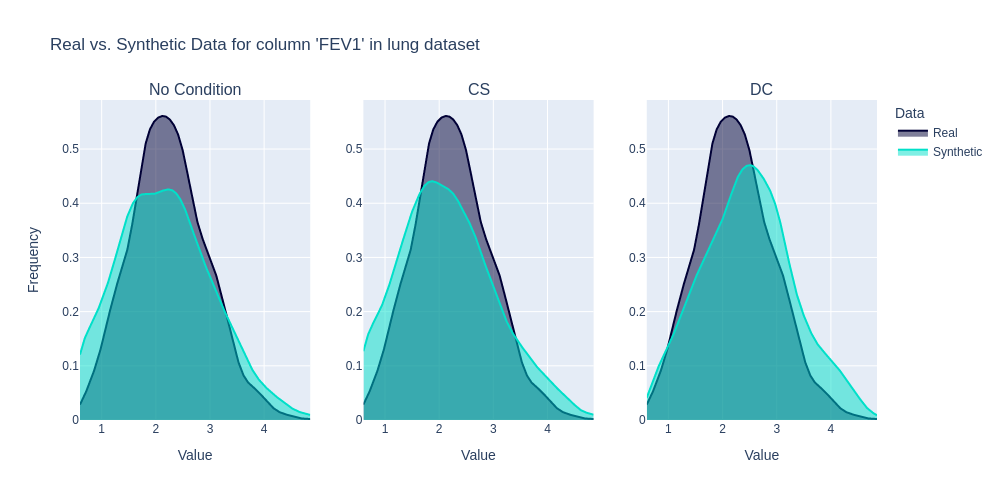


Figure A3-9. Data quality of “FEV1” column in the lung cancer data.


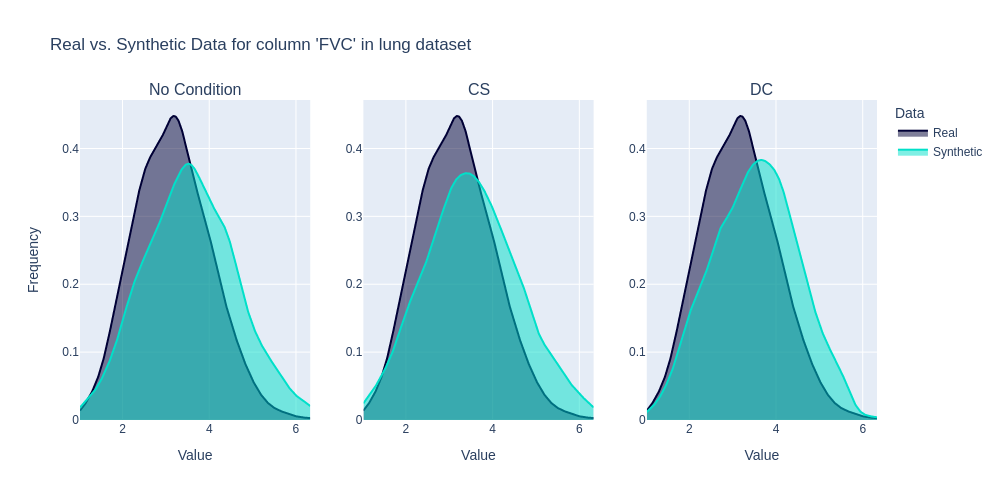


Figure A3-10. Data quality of “FVC” column in the lung cancer data.


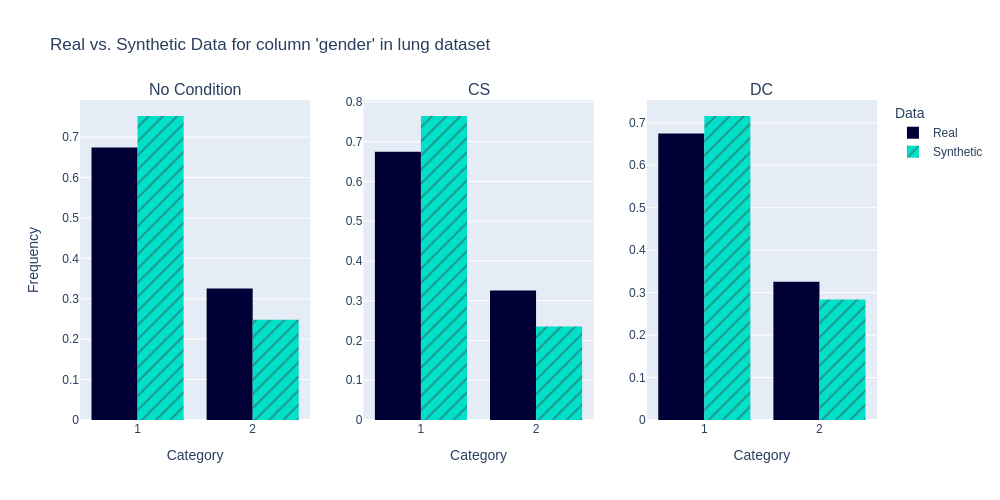


Figure A3-11. Data quality of “gender” column in the lung cancer data.


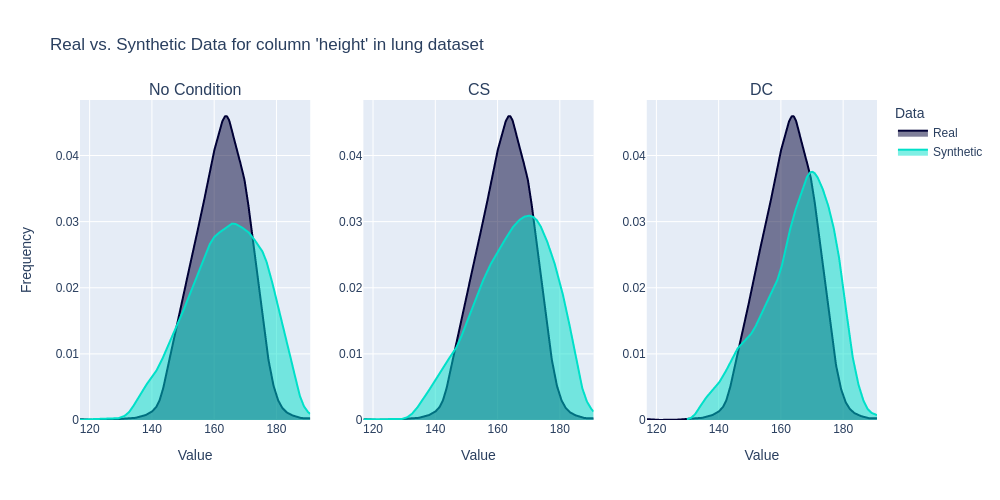


Figure A3-12. Data quality of “height” column in the lung cancer data.


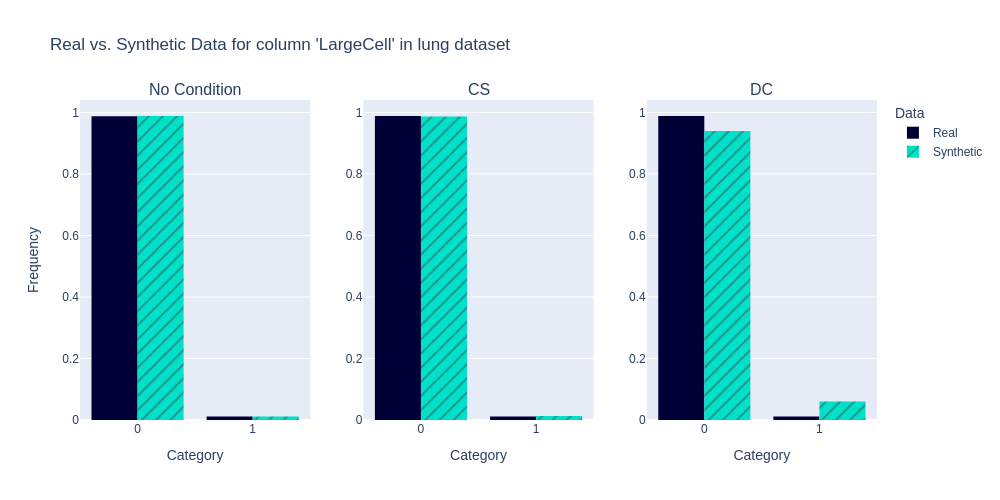


Figure A3-13. Data quality of “large cell” column in the lung cancer data.


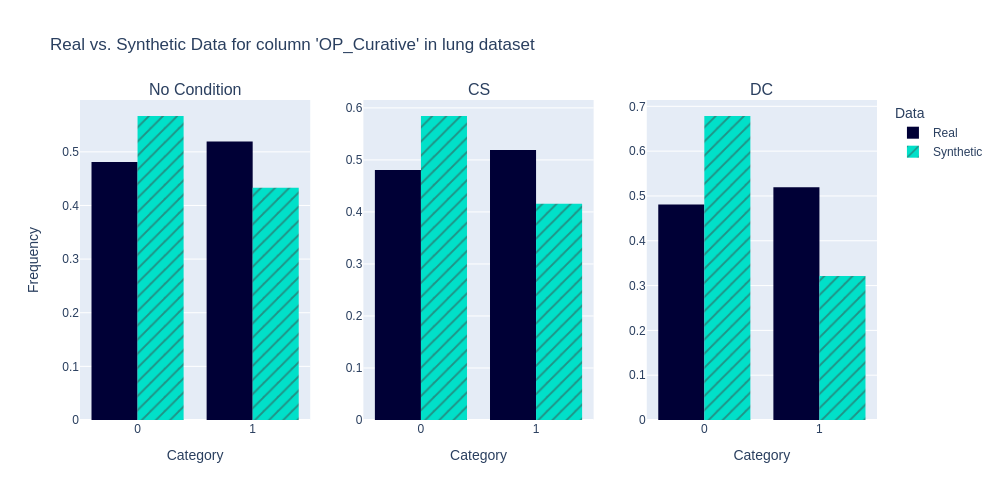


Figure A3-14. Data quality of “OP curative” column in the lung cancer data.


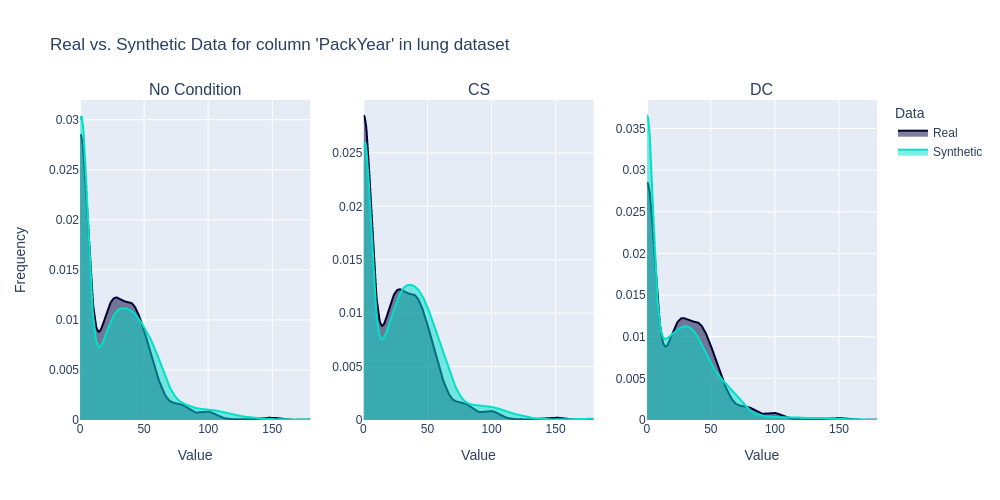


Figure A3-15. Data quality of “pack year” column in the lung cancer data.


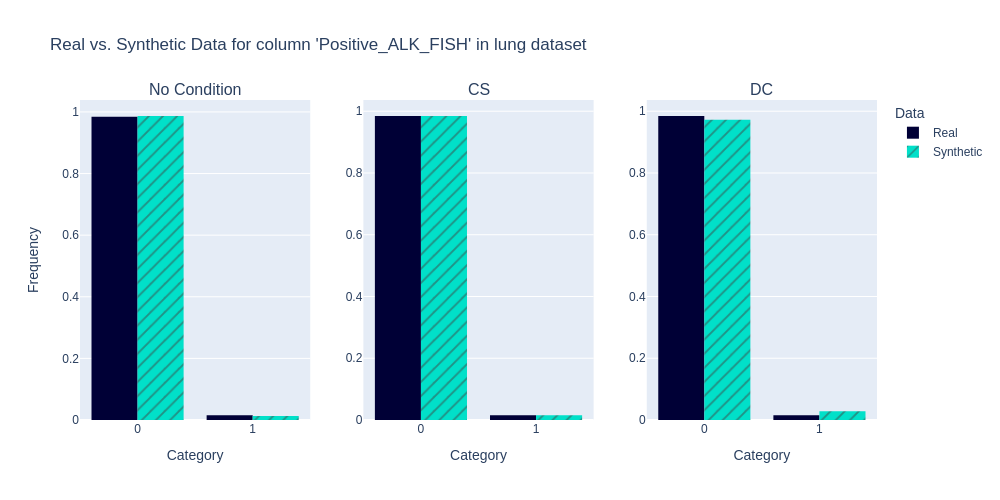


Figure A3-16. Data quality of “positive ALK FISH” column in the lung cancer data.


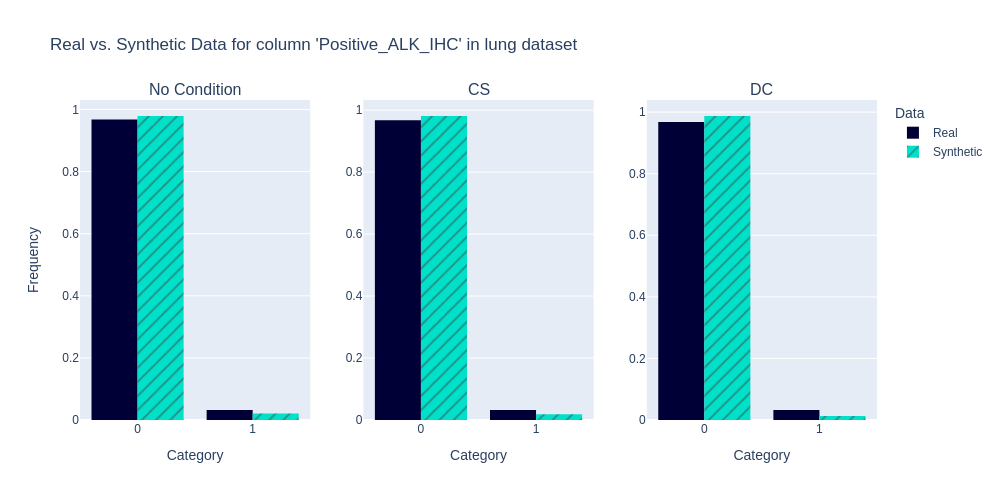


Figure A3-17. Data quality of “positive ALK IHC” column in the lung cancer data.


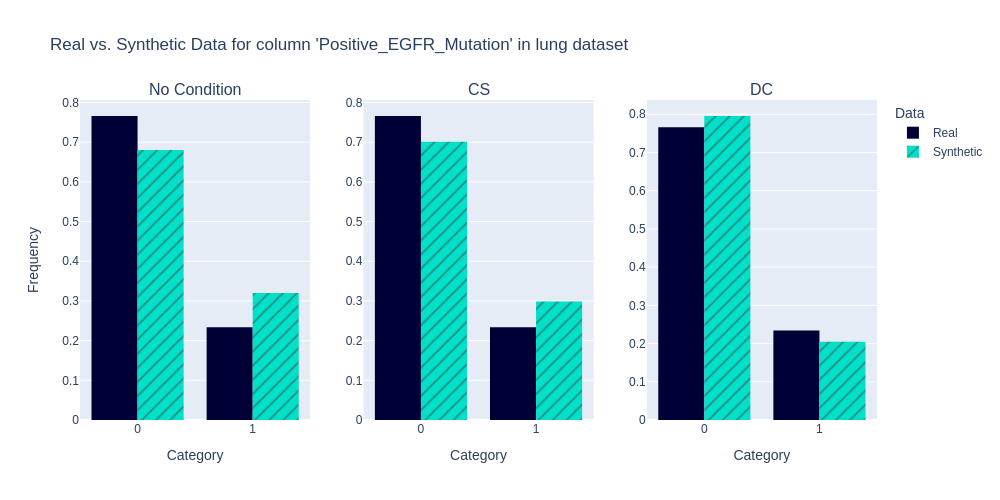


Figure A3-18. Data quality of “positive EGFR mutation” column in the lung cancer data.


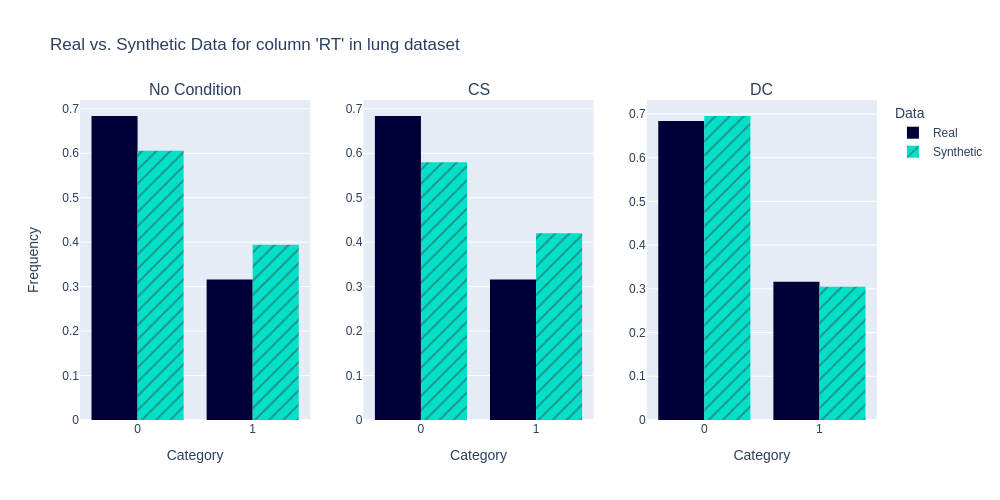


Figure A3-19. Data quality of “RT” column in the lung cancer data.


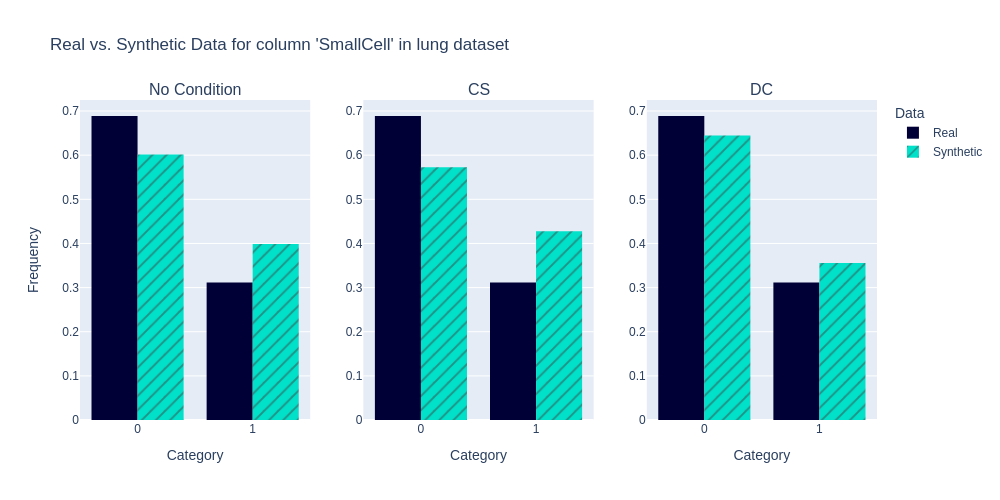


Figure A3-20. Data quality of “small cell” column in the lung cancer data.


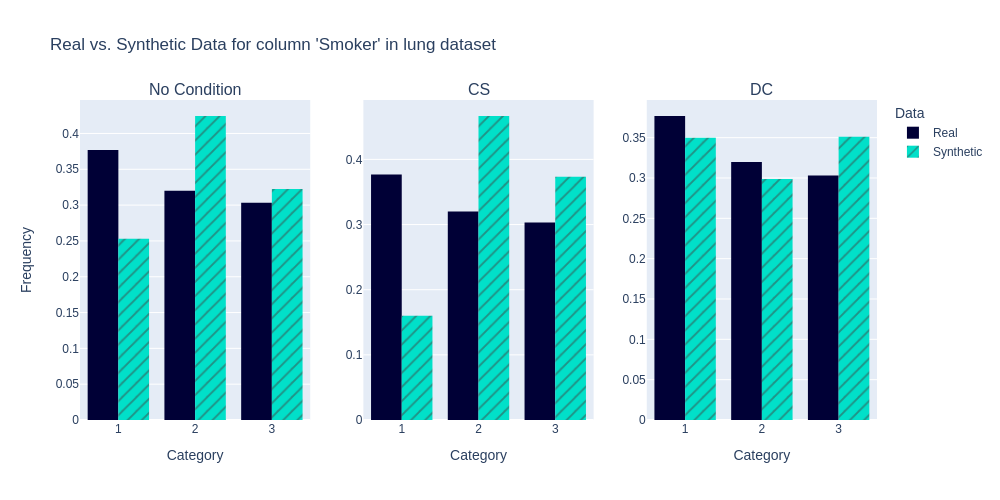


Figure A3-21. Data quality of “smoker” column in the lung cancer data.


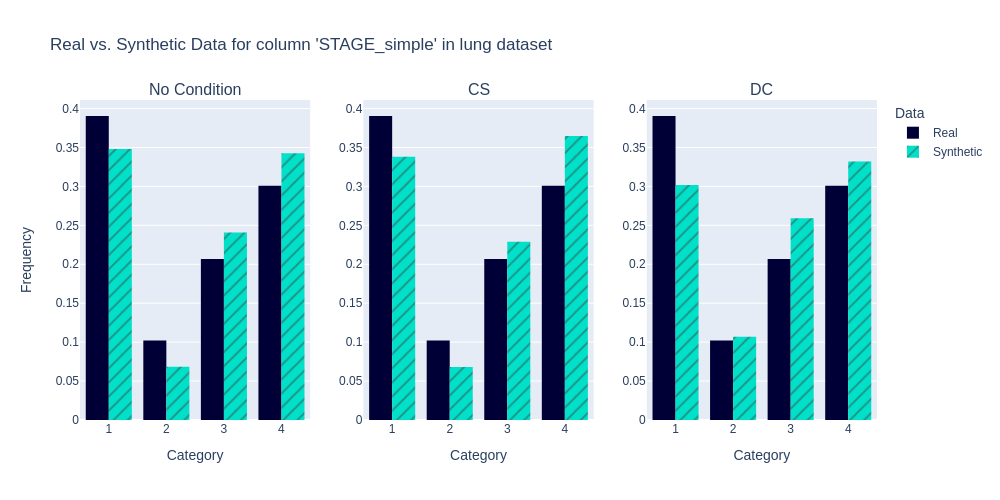


Figure A3-22. Data quality of “stage simple” column in the lung cancer data.


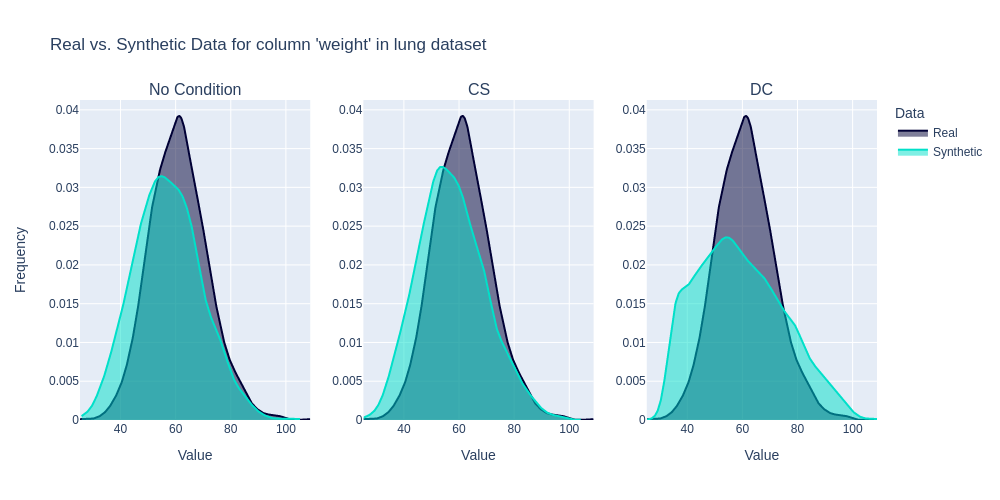


Figure A3-23. Data quality of “weight” column in the lung cancer data.


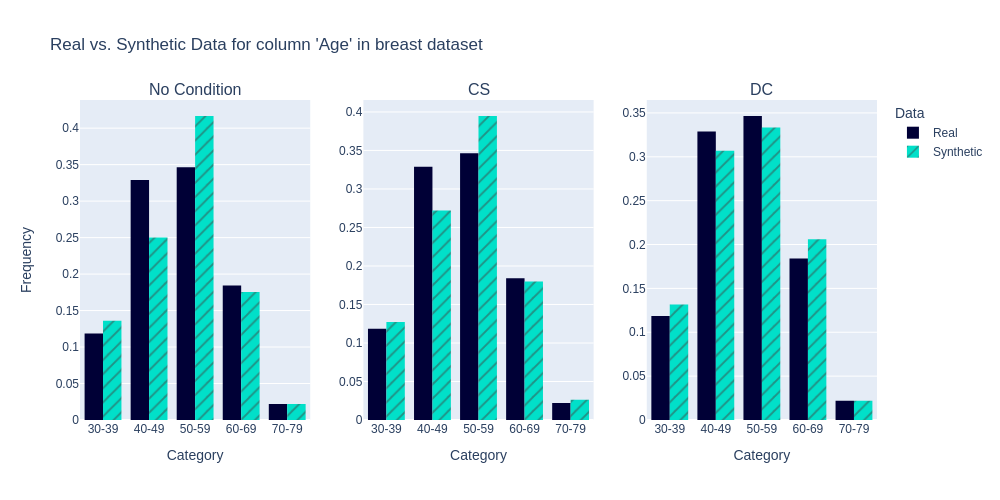


Figure A3-24. Data quality of “age” column in the breast cancer data.


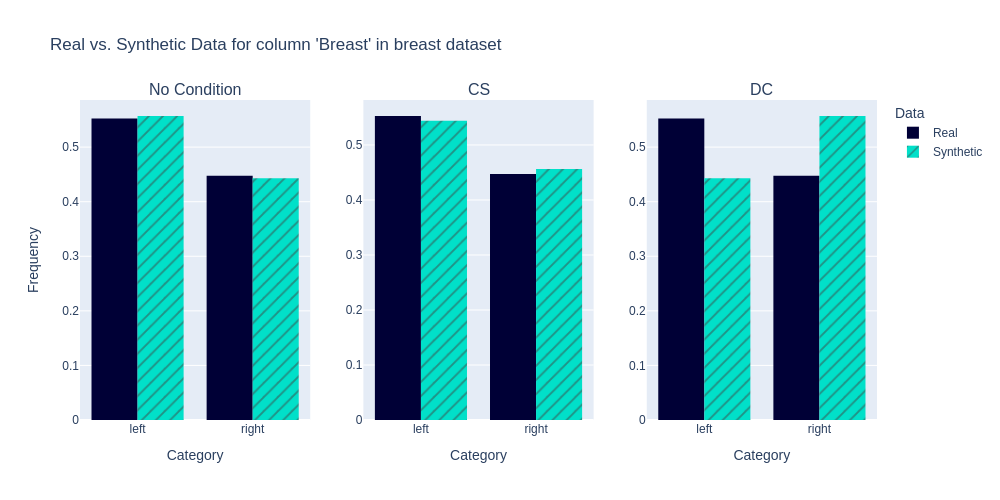


Figure A3-25. Data quality of “breast” column in the breast cancer data.


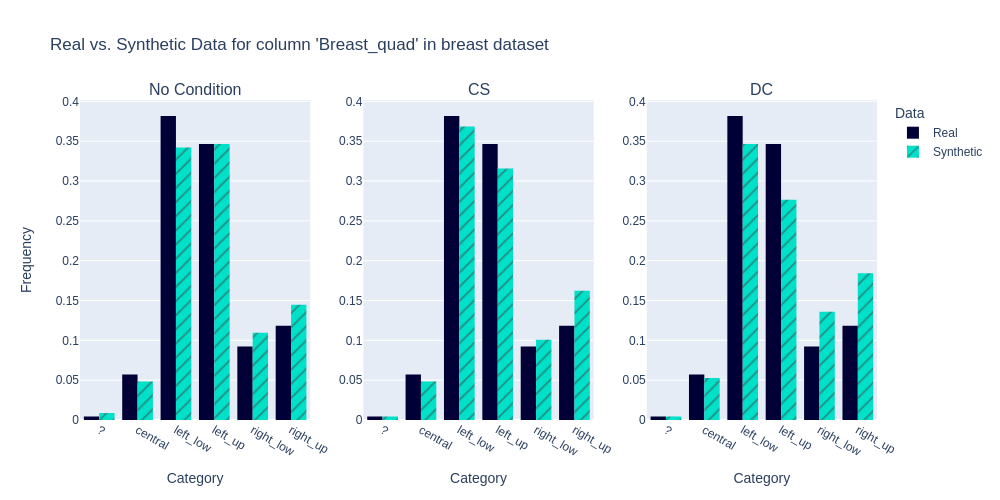


Figure A3-26. Data quality of “breast quad” column in the breast cancer data.


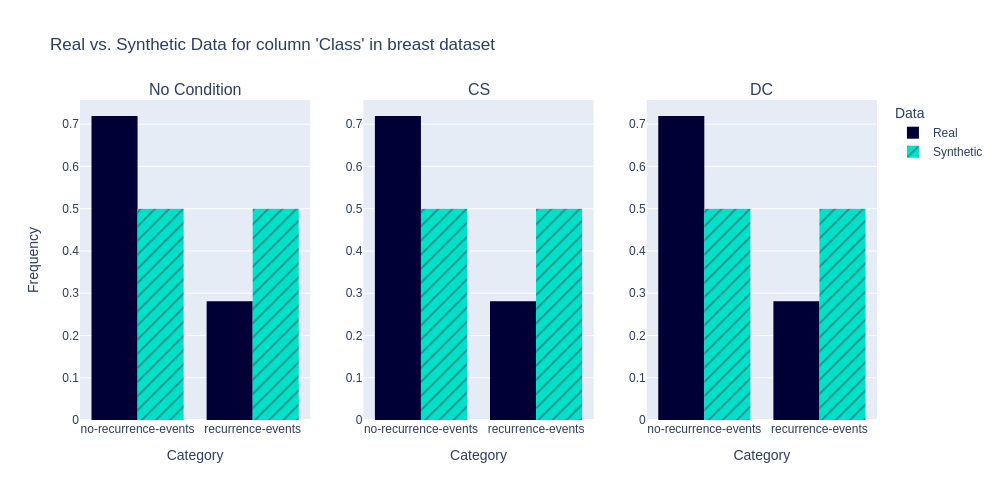


Figure A3-27. Data quality of “class” column in the breast cancer data.


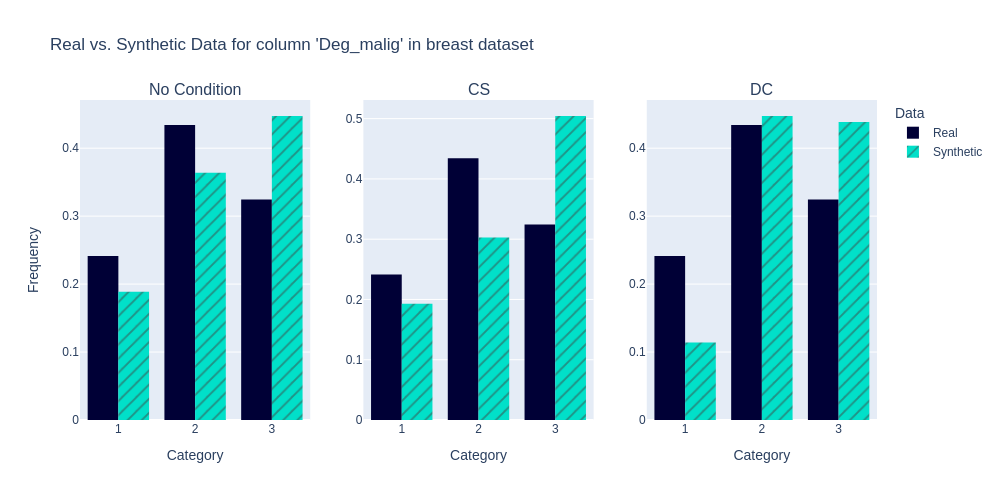


Figure A3-28. Data quality of “deg malig” column in the breast cancer data.


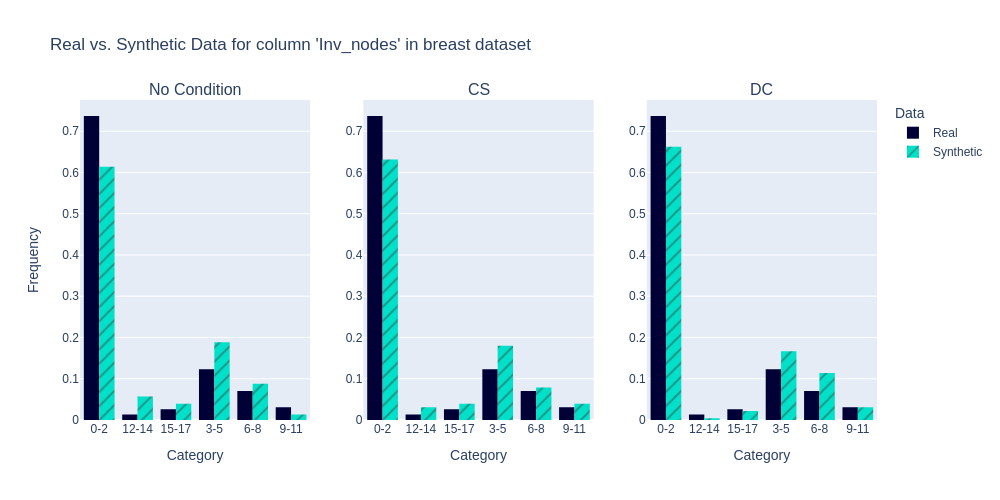


Figure A3-29. Data quality of “inv nodes” column in the breast cancer data.


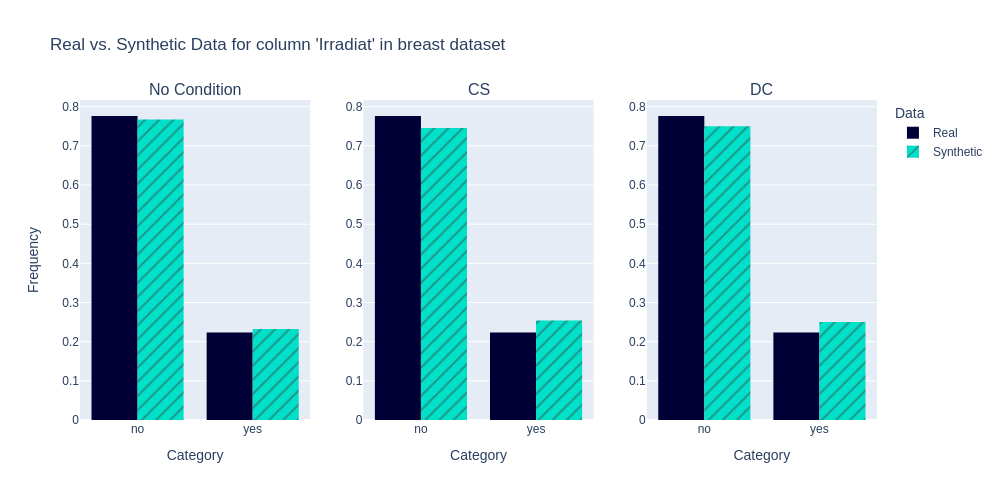


Figure A3-30. Data quality of “irradiat” column in the breast cancer data.


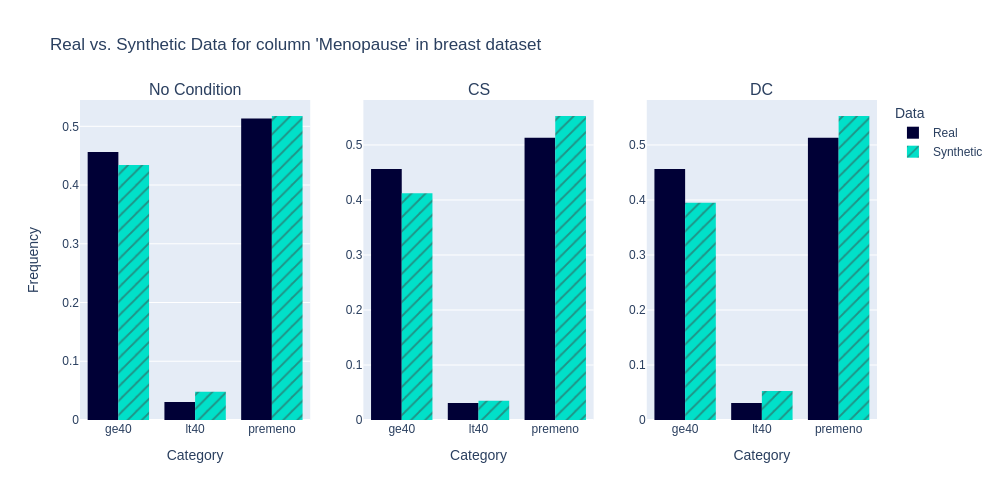


Figure A3-31. Data quality of “menopause” column in the breast cancer data.


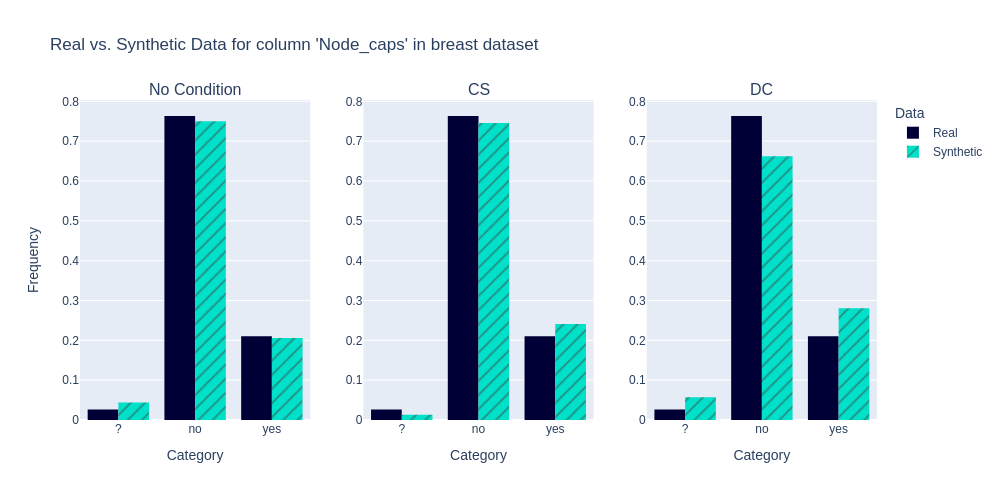


Figure A3-32. Data quality of “node caps” column in the breast cancer data.


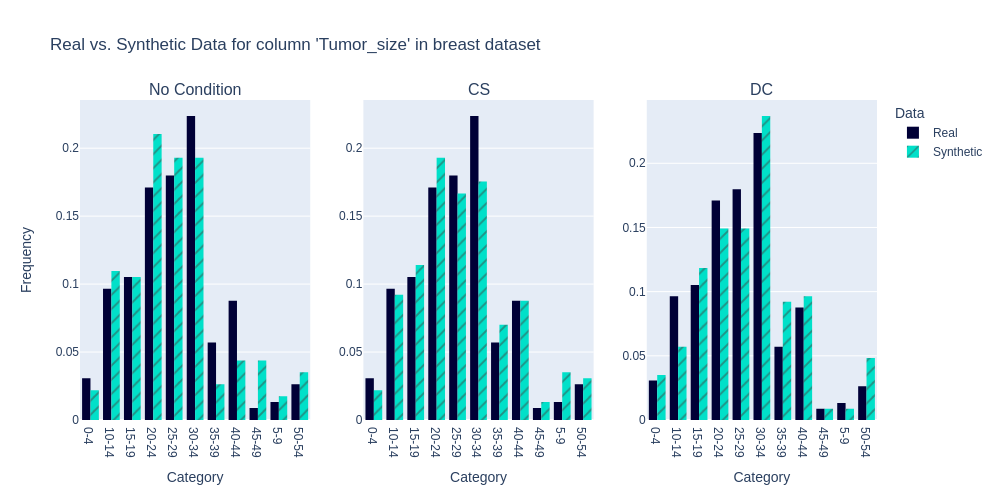


Figure A3-33. Data quality of “tumor size” column in the breast cancer data.


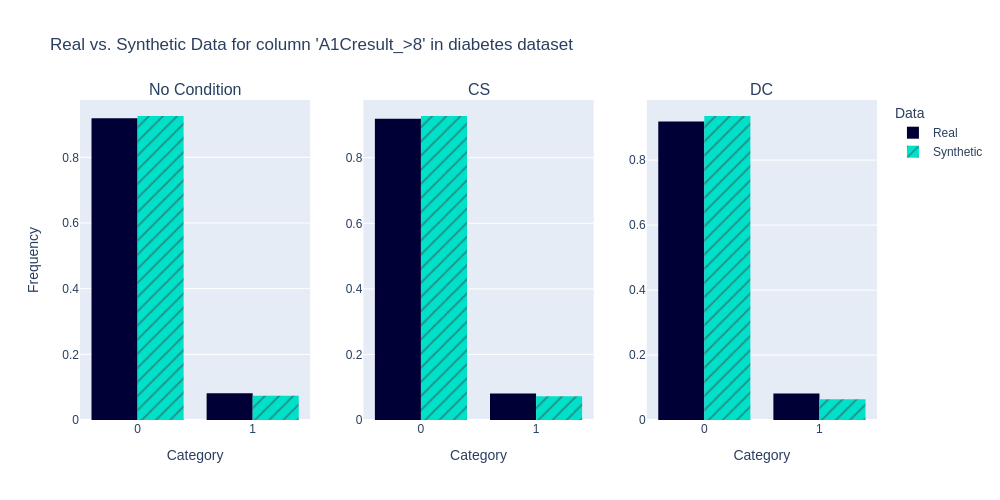


Figure A3-34. Data quality of “A1C result > 8” column in the diabetes data.


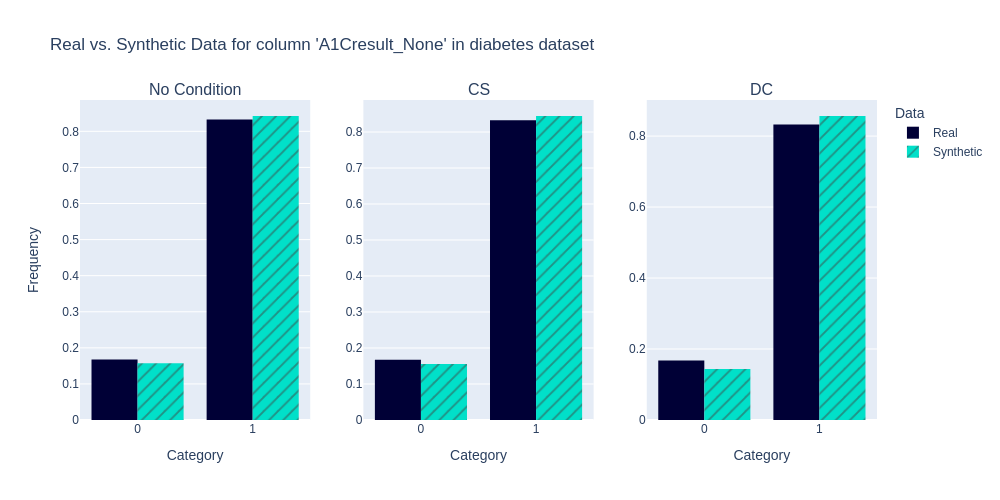


Figure A3-35. Data quality of “A1C result none” column in the diabetes data.


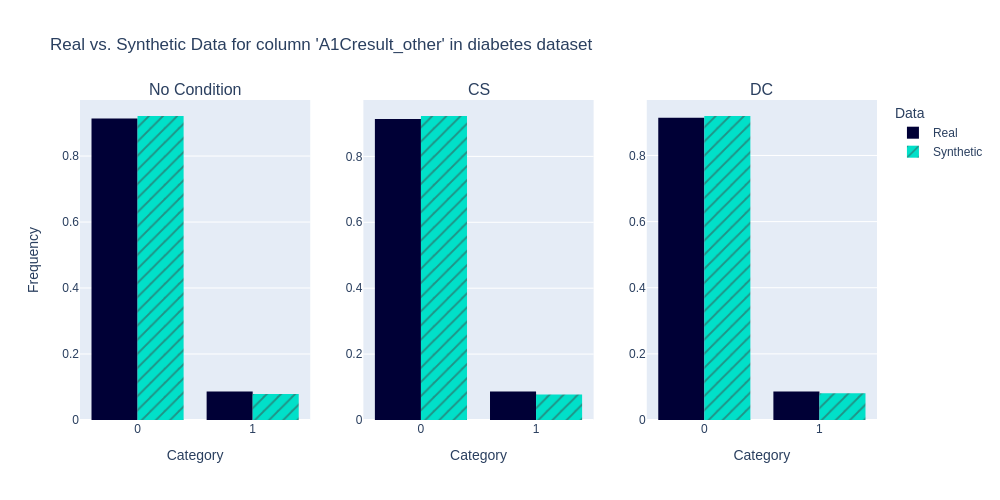


Figure A3-36. Data quality of “A1C result other” column in the diabetes data.


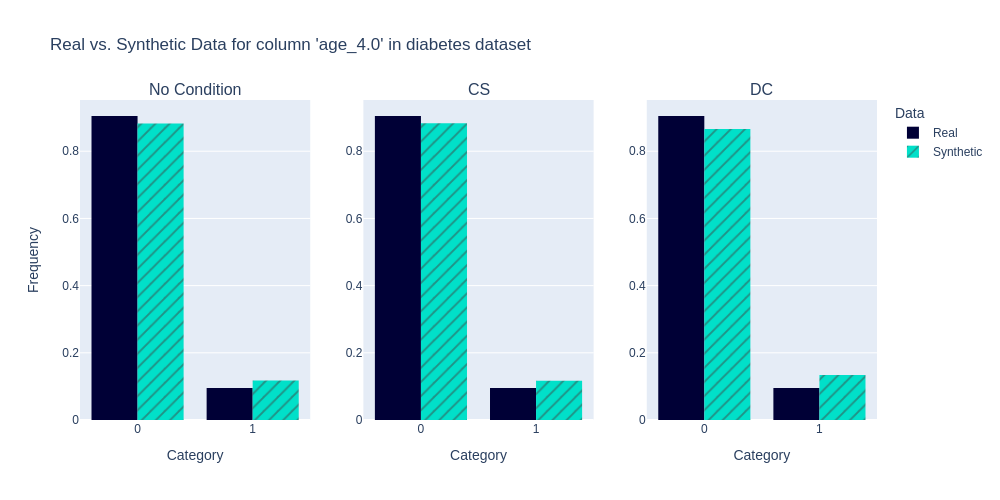


Figure A3-37. Data quality of “age 4.0” column in the diabetes data.


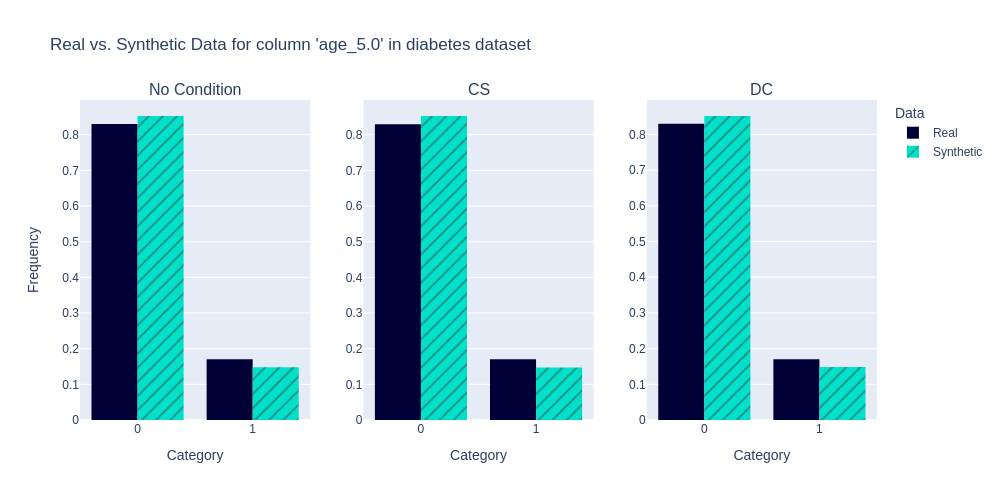


Figure A3-38. Data quality of “age 5.0” column in the diabetes data.


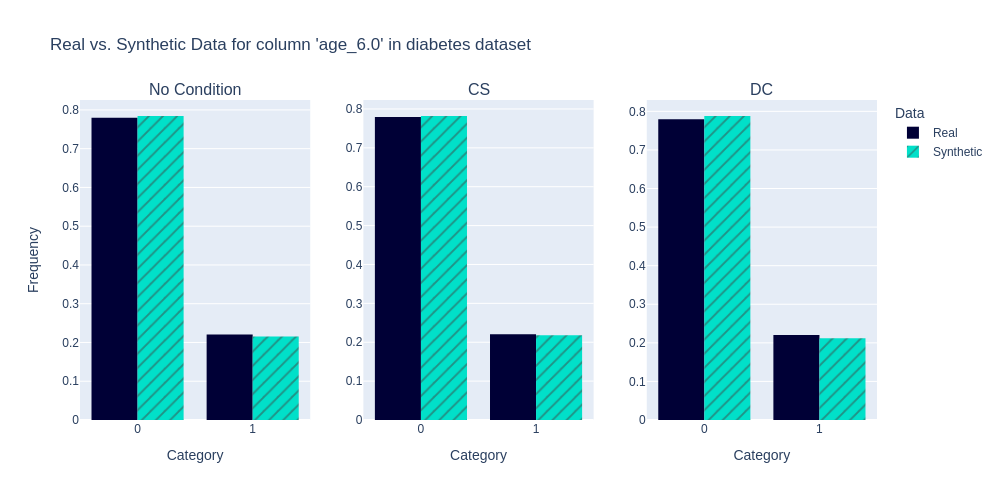


Figure A3-39. Data quality of “age 6.0” column in the diabetes data.


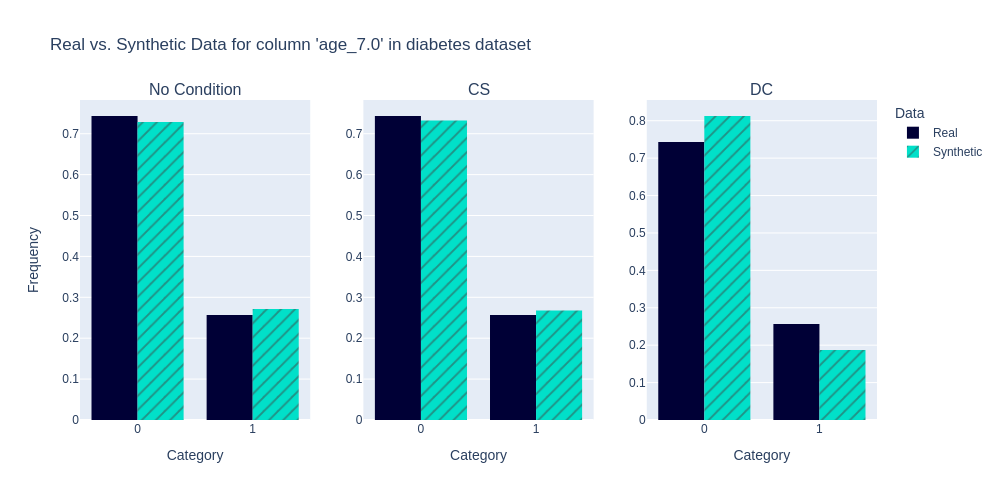


Figure A3-40. Data quality of “age 7.0” column in the diabetes data.


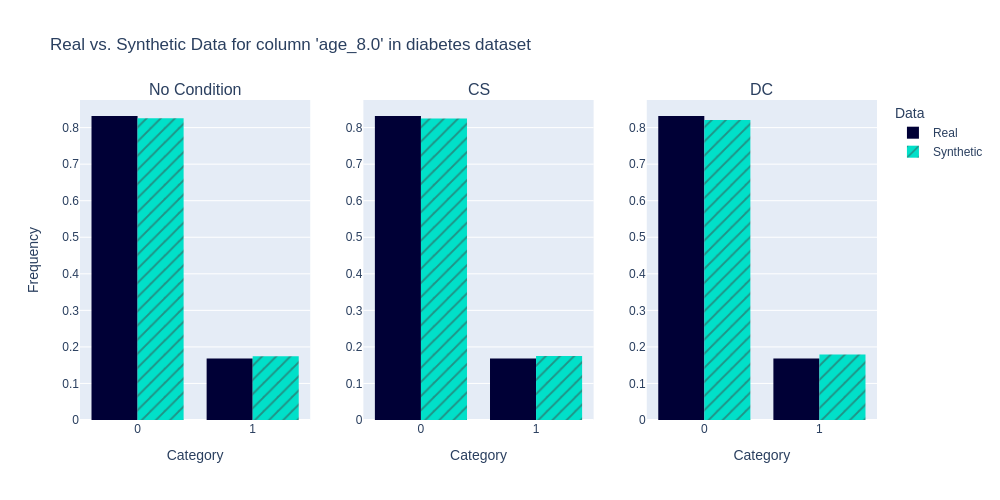


Figure A3-41. Data quality of “age 8.0” column in the diabetes data.


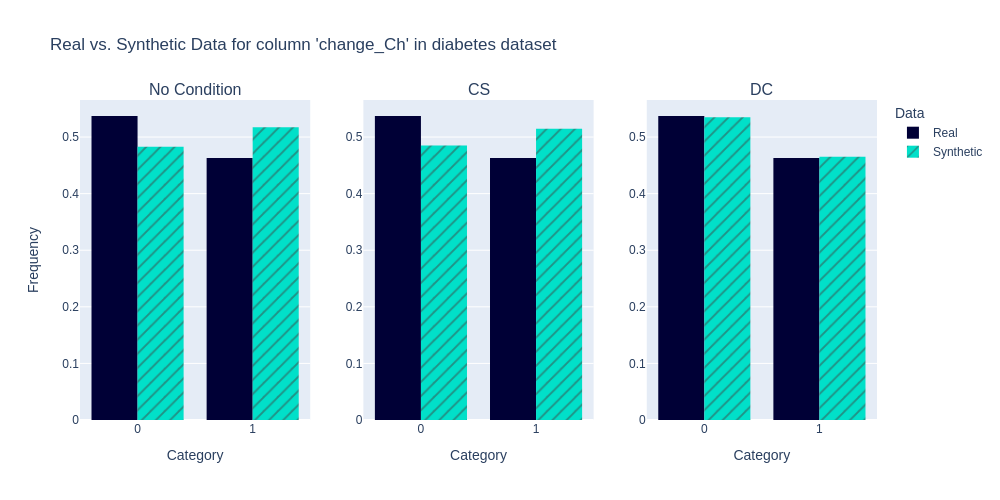


Figure A3-42. Data quality of “change ch” column in the diabetes data.


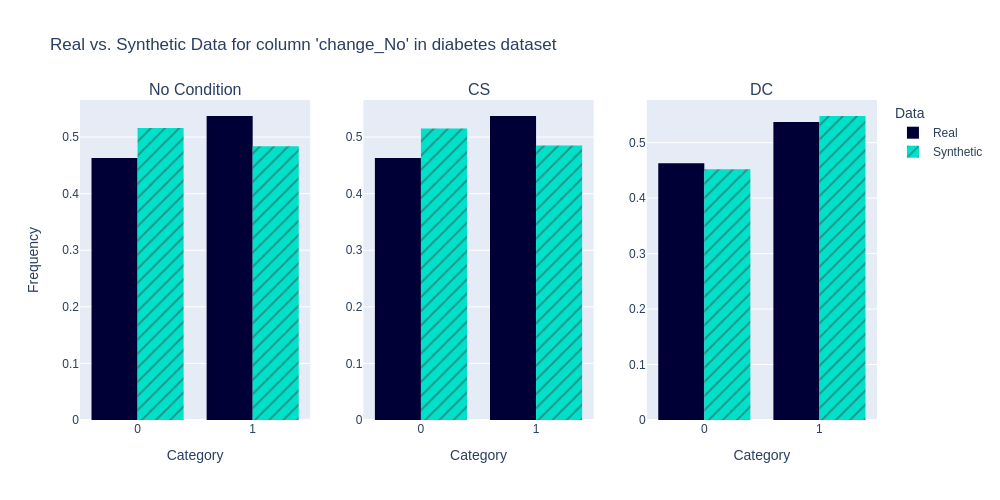


Figure A3-43. Data quality of “change no” column in the diabetes data.


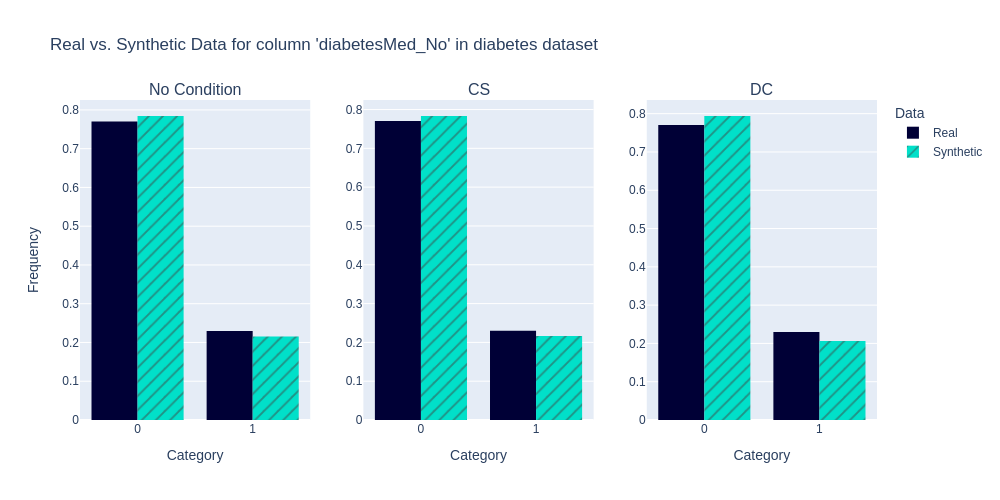


Figure A3-44. Data quality of “diabetes med no” column in the diabetes data.


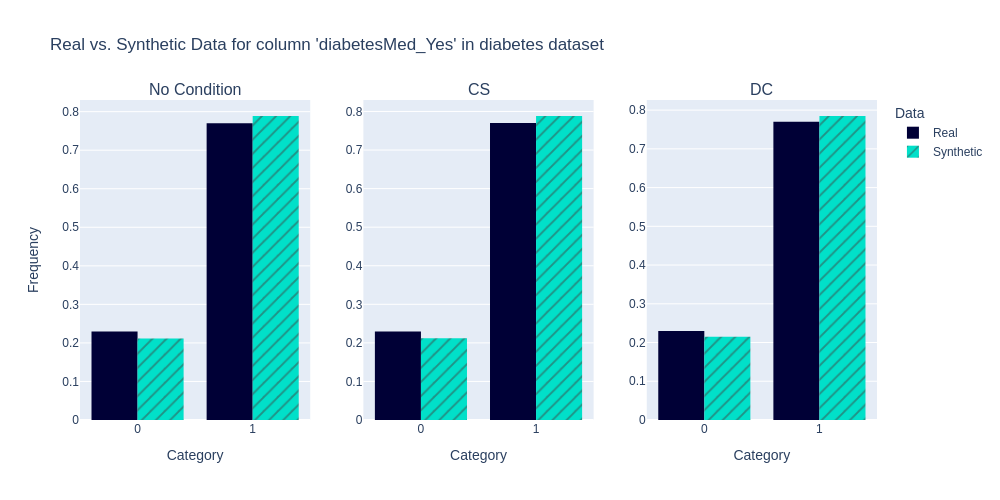


Figure A3-45. Data quality of “diabetes med yes” column in the diabetes data.


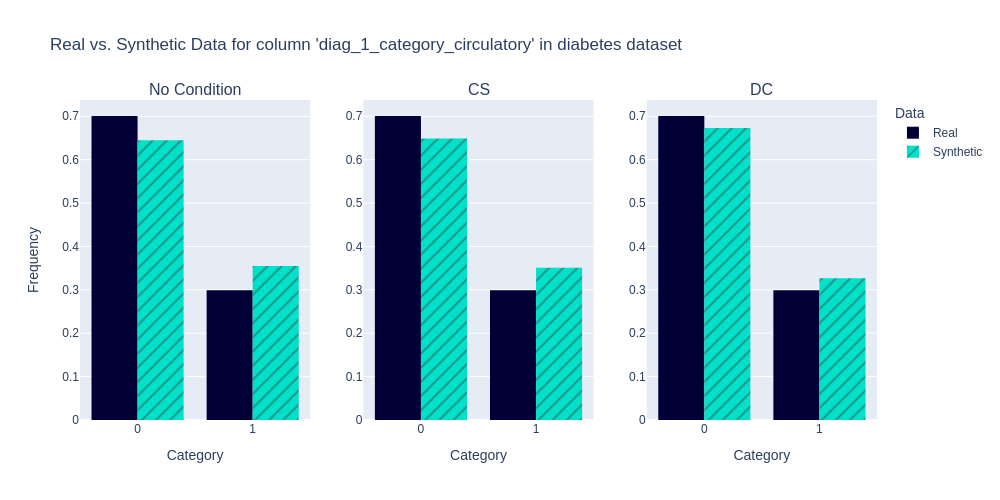


Figure A3-46. Data quality of “diag 1 category circulatory” column in the diabetes data.


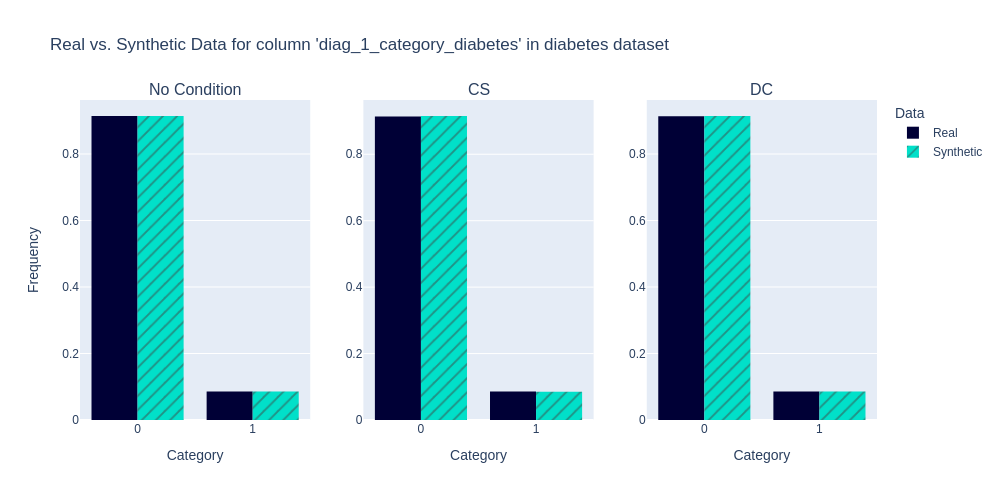


Figure A3-47. Data quality of “diag 1 category diabetes” column in the diabetes data.


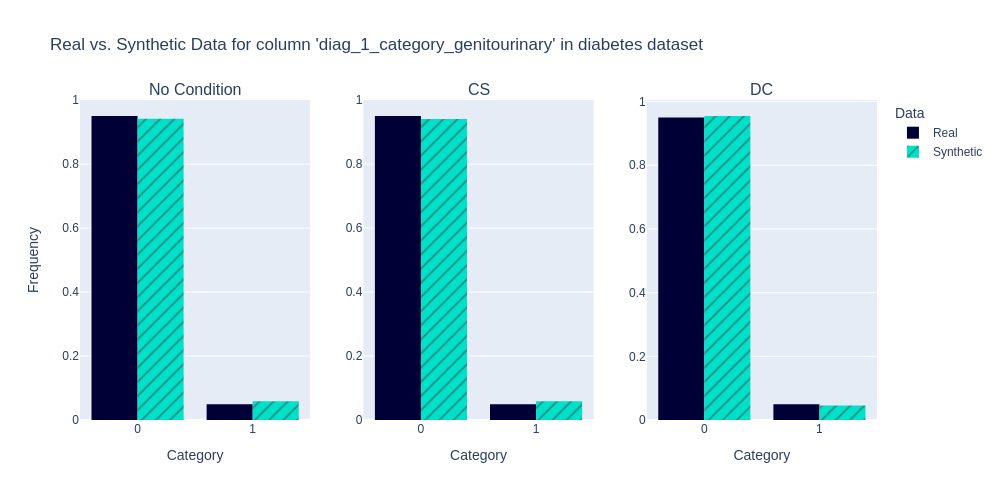


Figure A3-48. Data quality of “diag 1 category genitourinary” column in the diabetes data.


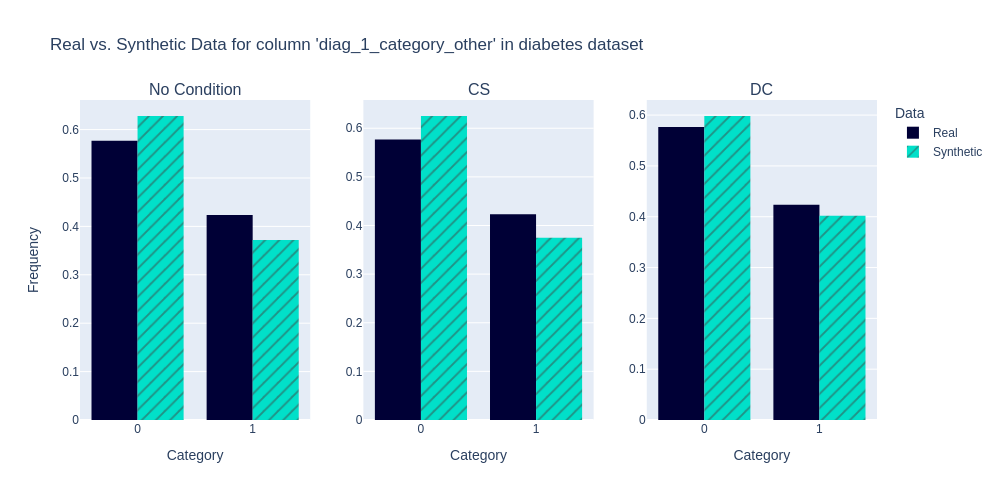


Figure A3-49. Data quality of “diag 1 category other” column in the diabetes data.


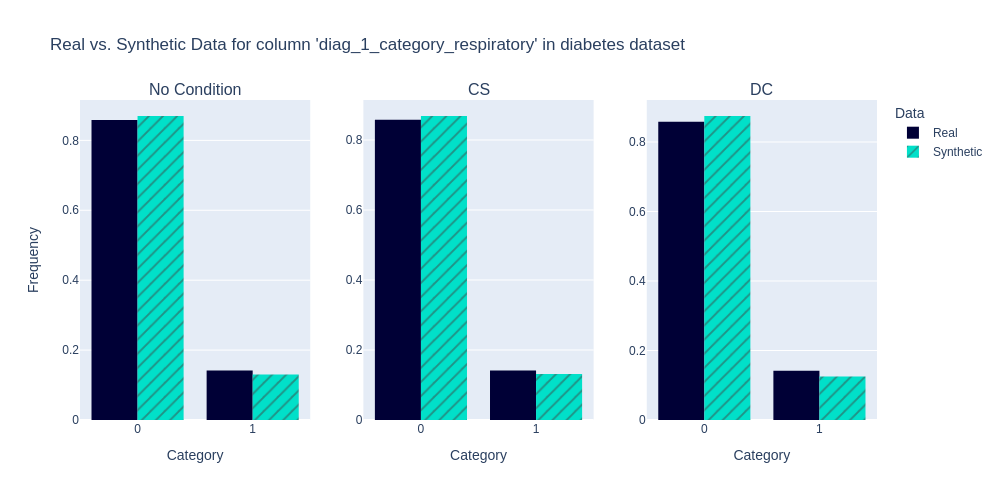


Figure A3-50. Data quality of “diag 1 category respiratory” column in the diabetes data.


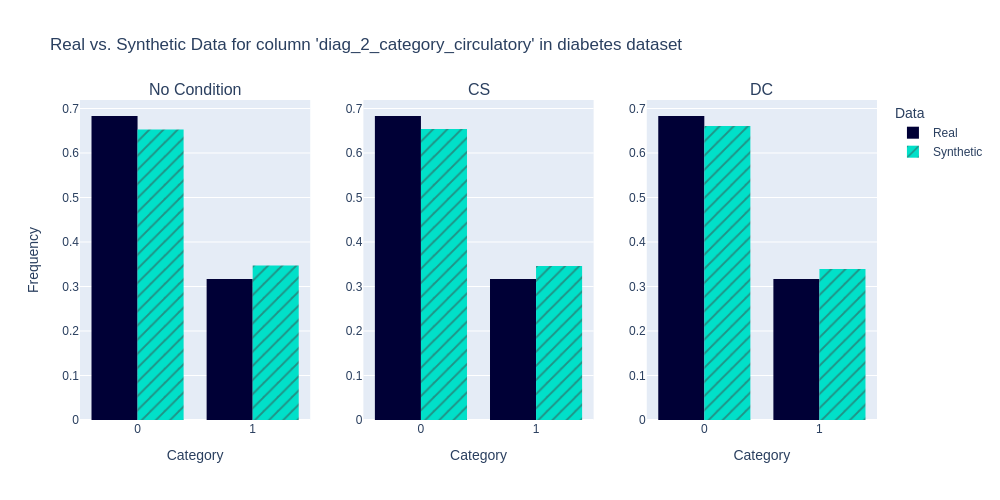


Figure A3-51. Data quality of “diag 2 category circulatory” column in the diabetes data.


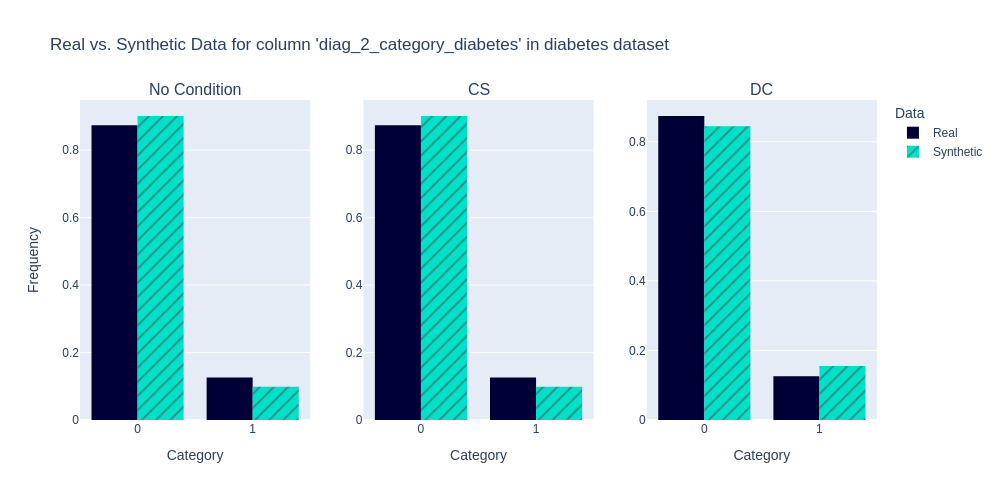


Figure A3-52. Data quality of “diag 2 category diabetes” column in the diabetes data.


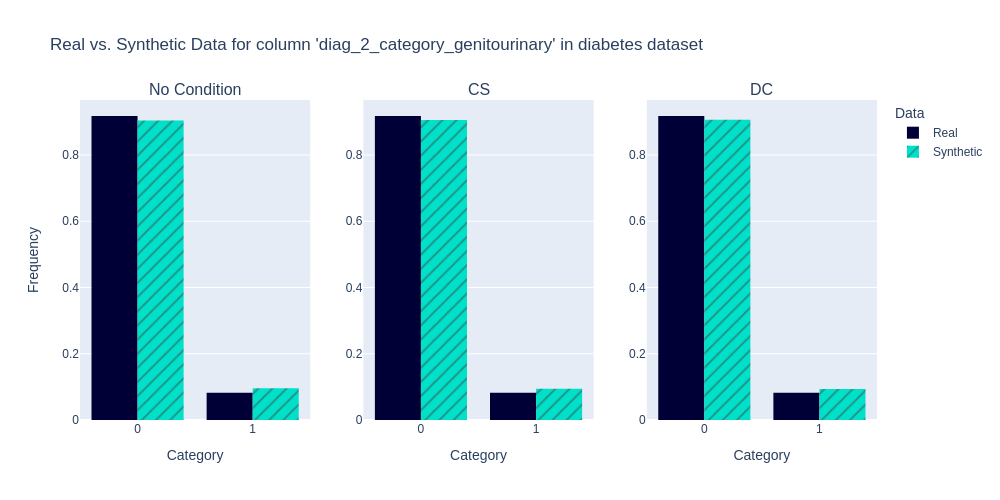


Figure A3-53. Data quality of “diag 2 category genitourinary” column in the diabetes data.


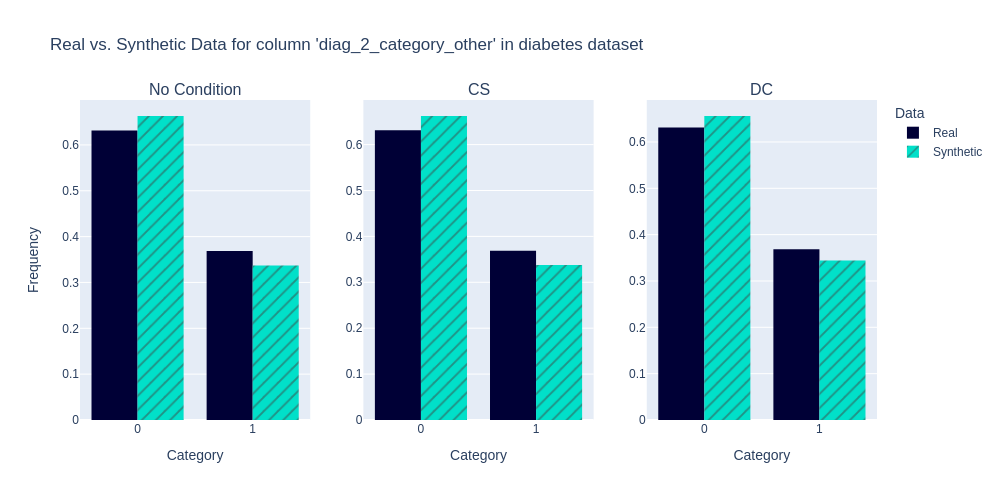


Figure A3-54. Data quality of “diag 2 category other” column in the diabetes data.


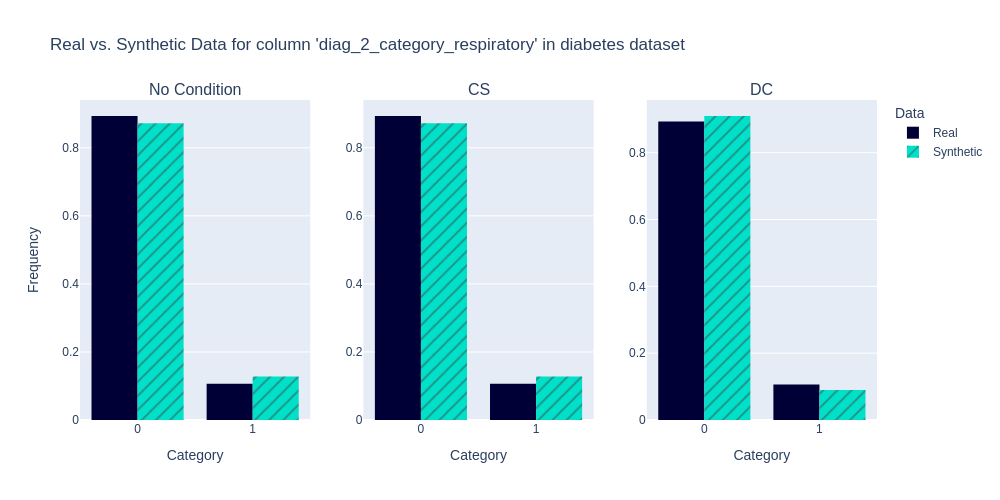


Figure A3-55. Data quality of “diag 2 category respiratory” column in the diabetes data.


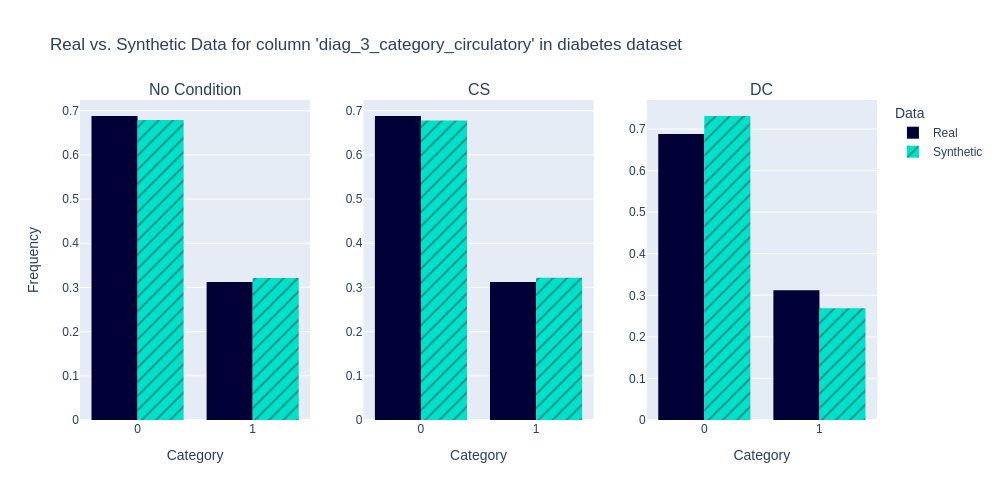


Figure A3-56. Data quality of “diag 3 category circulatory” column in the diabetes data.


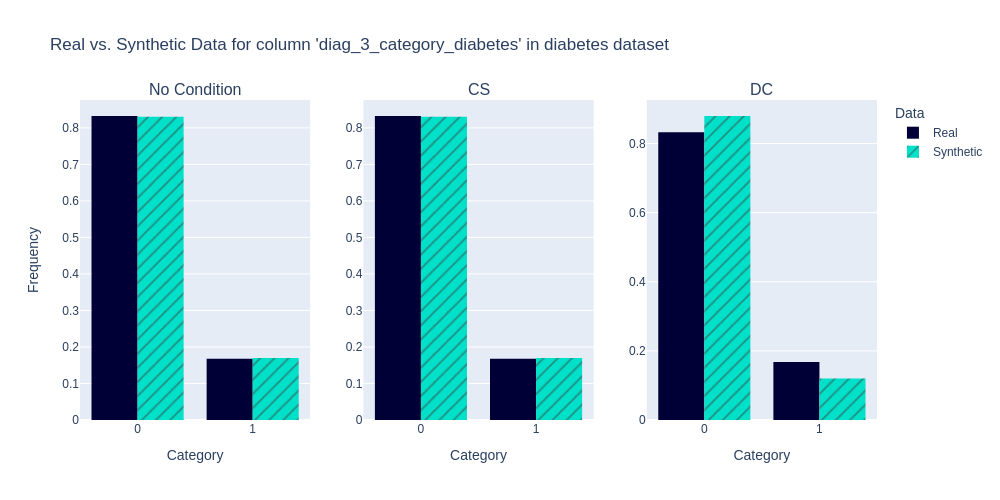


Figure A3-57. Data quality of “diag 3 category diabetes” column in the diabetes data.


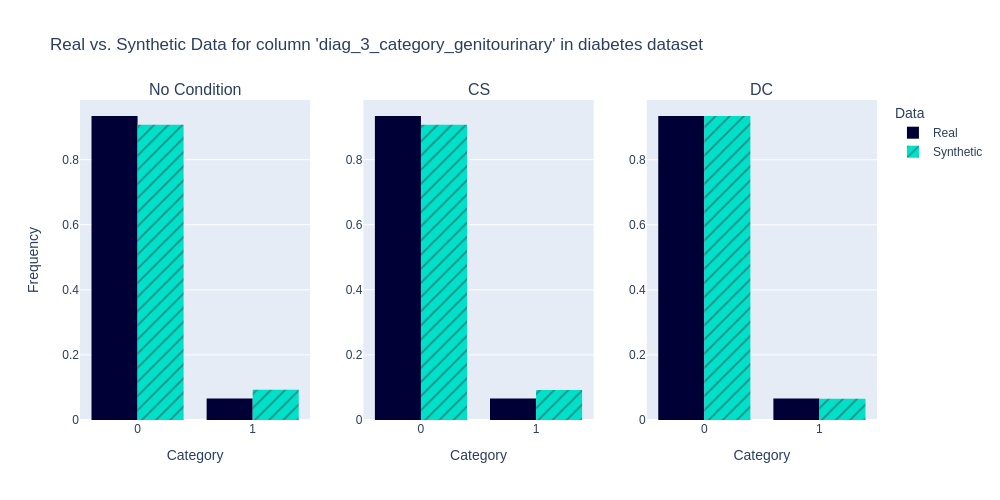


Figure A3-58. Data quality of “diag 3 category genitourinary” column in the diabetes data.


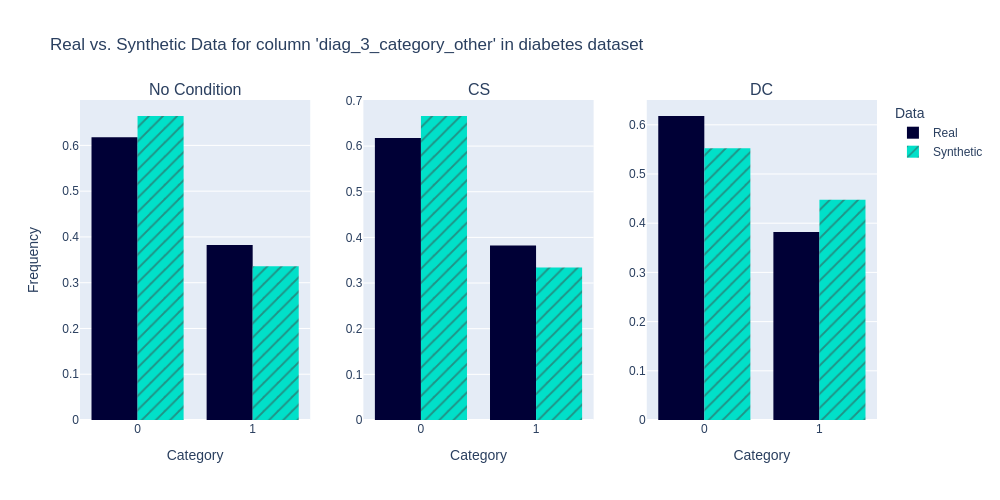


Figure A3-59. Data quality of “diag 3 category other” column in the diabetes data.


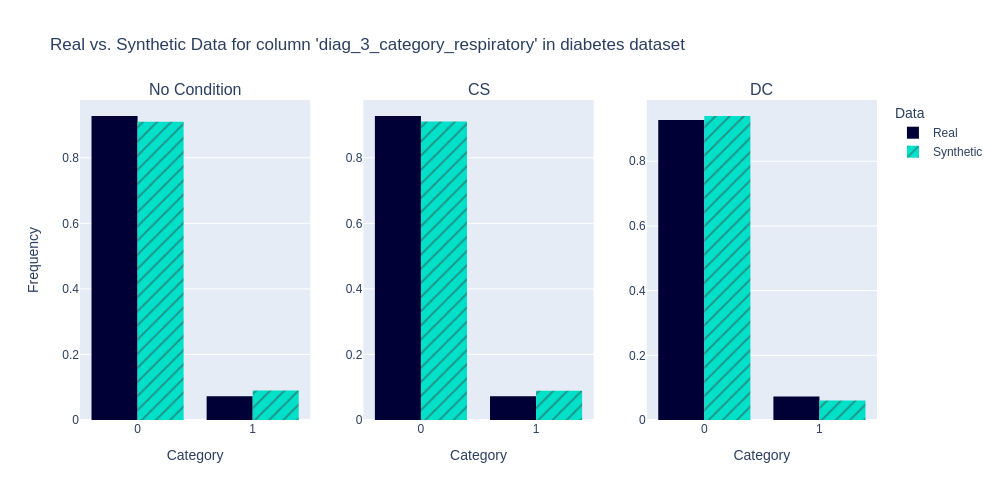


Figure A3-60. Data quality of “diag 3 category respiratory” column in the diabetes data.


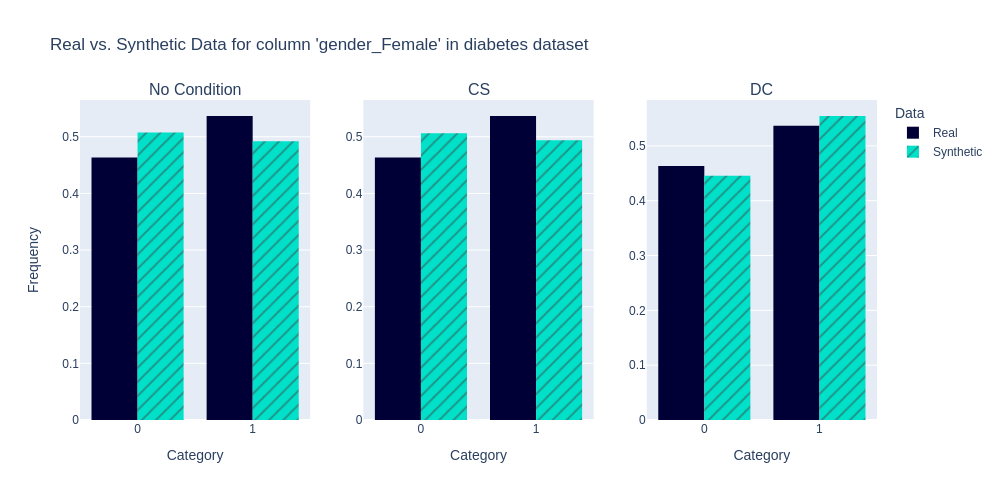


Figure A3-61. Data quality of “gender female” column in the diabetes data.


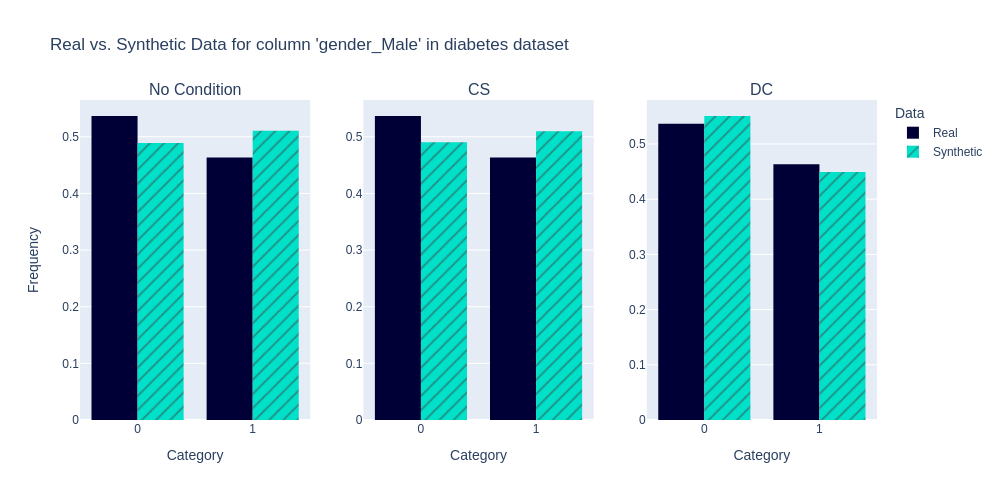


Figure A3-62. Data quality of “gender male” column in the diabetes data.


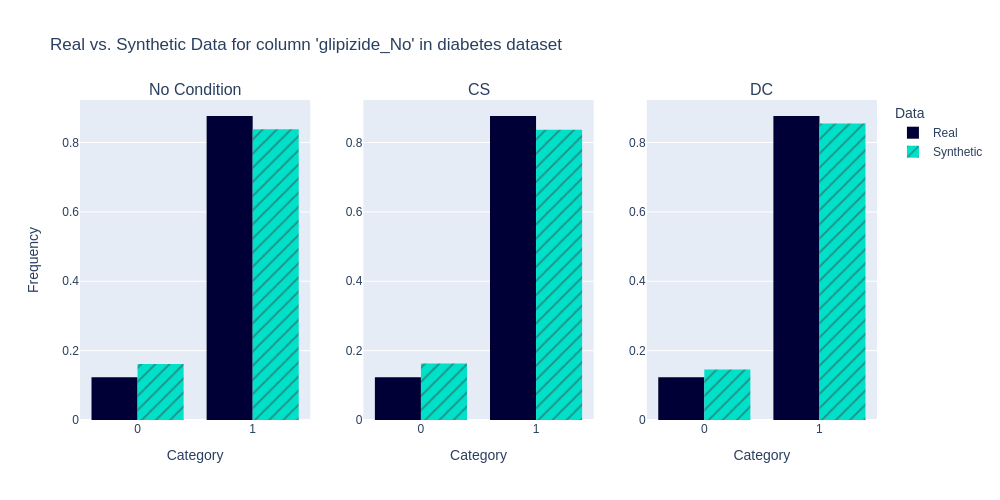


Figure A3-63. Data quality of “glipizide no” column in the diabetes data.


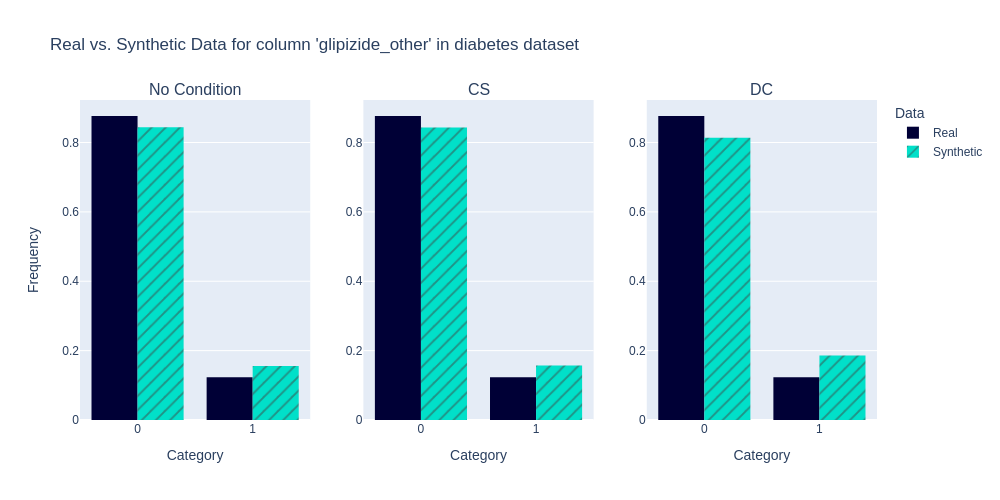


Figure A3-64. Data quality of “glipizide other” column in the diabetes data.


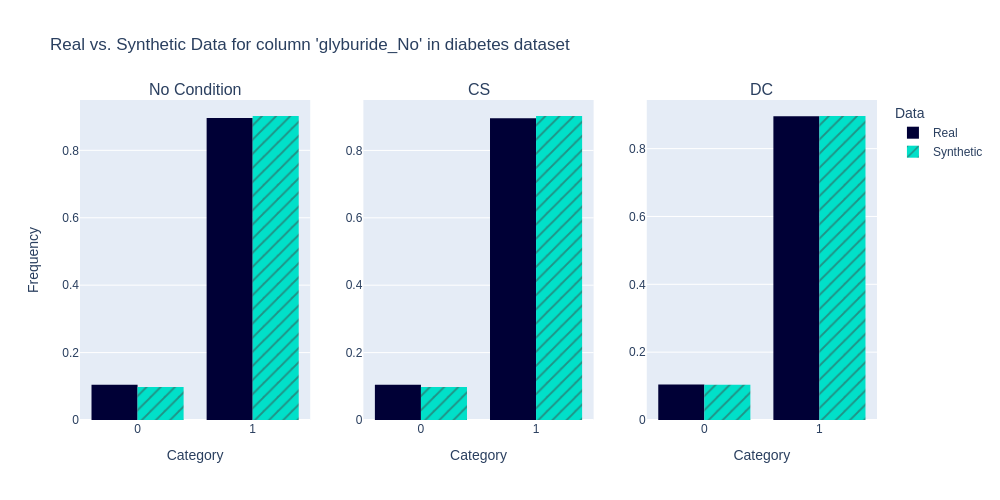


Figure A3-65. Data quality of “glyburide no” column in the diabetes data.


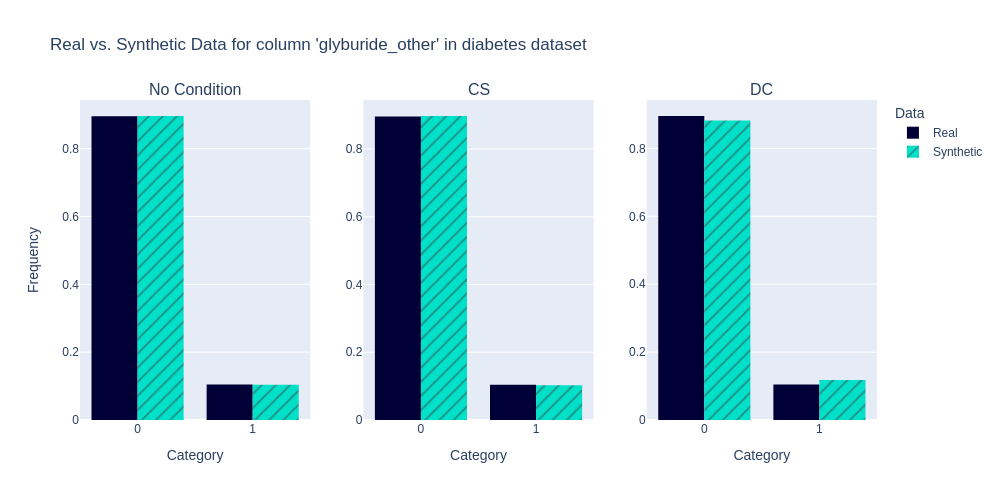


Figure A3-66. Data quality of “glyburide other” column in the diabetes data.


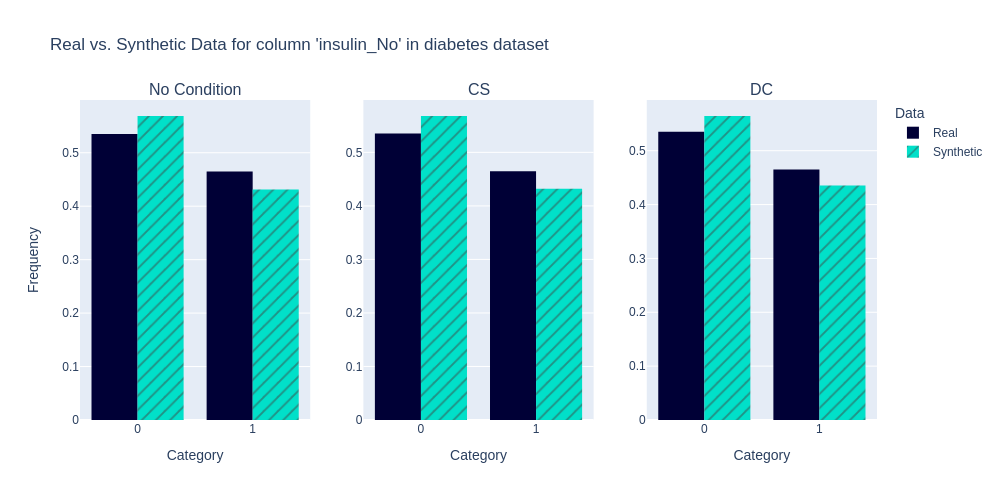


Figure A3-67. Data quality of “insulin no” column in the diabetes data.


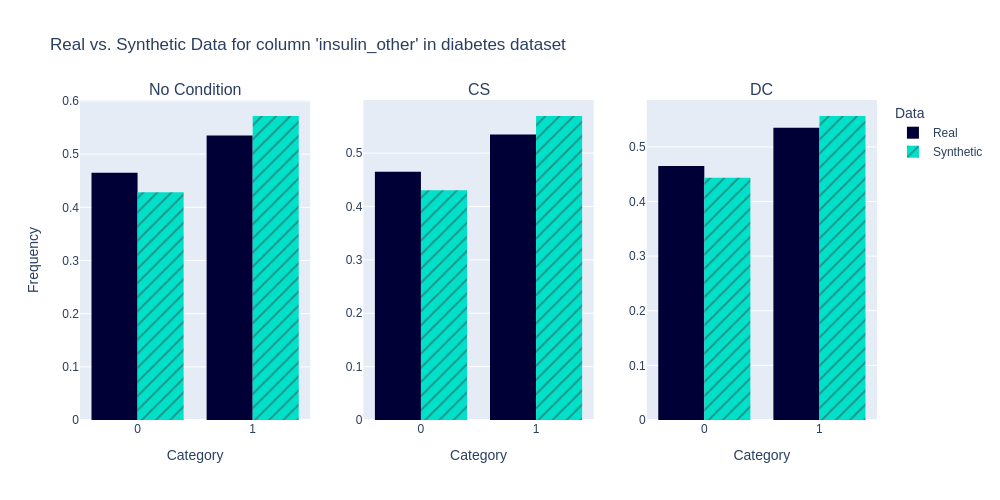


Figure A3-68. Data quality of “insulin other” column in the diabetes data.


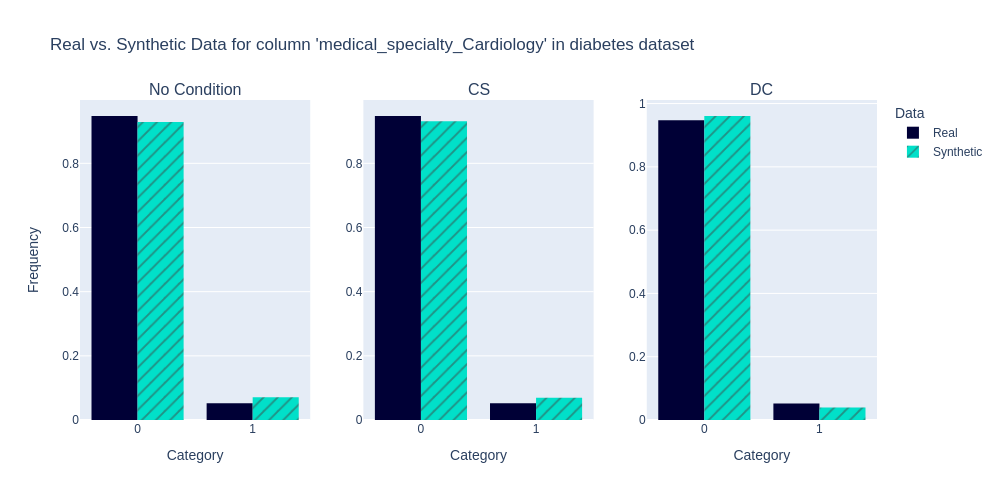


Figure A3-69. Data quality of “medical specialty cardiology” column in the diabetes data.


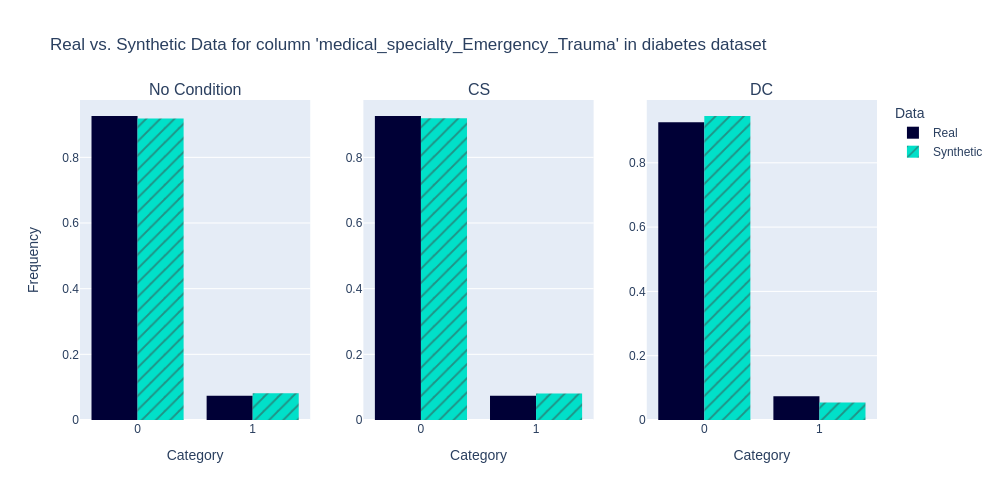


Figure A3-70. Data quality of “medical specialty emergency trauma” column in the diabetes data.


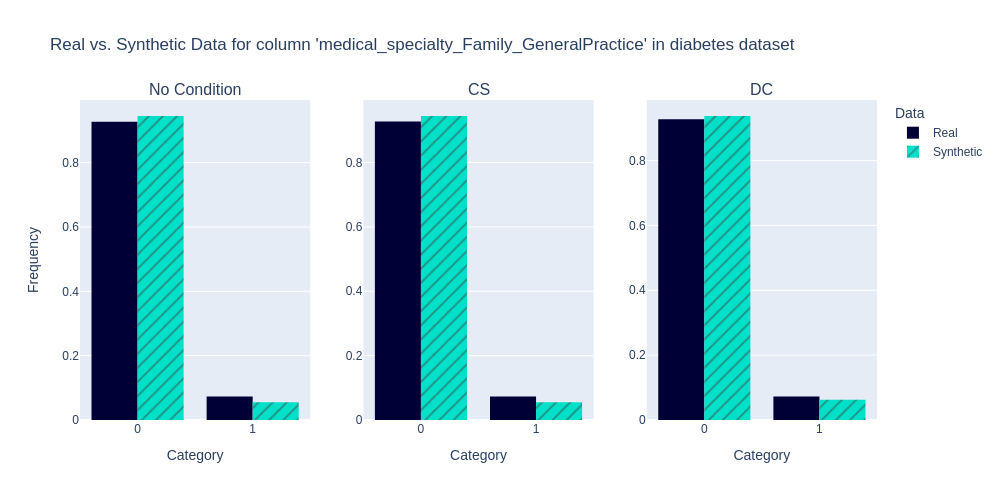


Figure A3-71. Data quality of “medical specialty family general practice” column in the diabetes data.


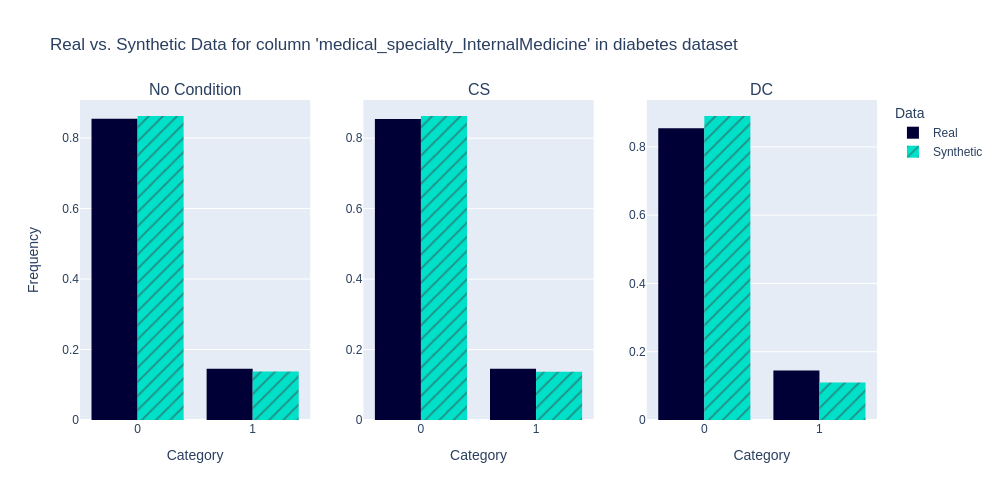


Figure A3-72. Data quality of “medical specialty internal medicine” column in the diabetes data.


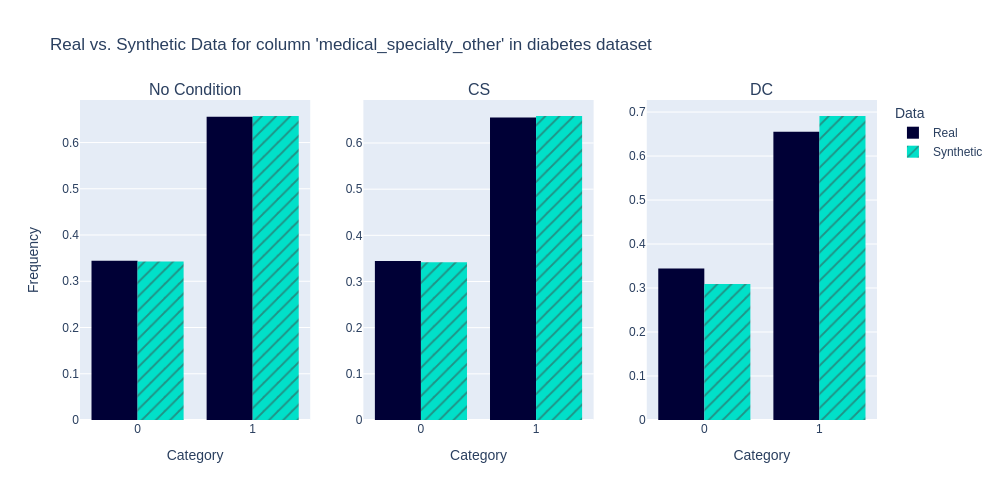


Figure A3-73. Data quality of “medical specialty other” column in the diabetes data.


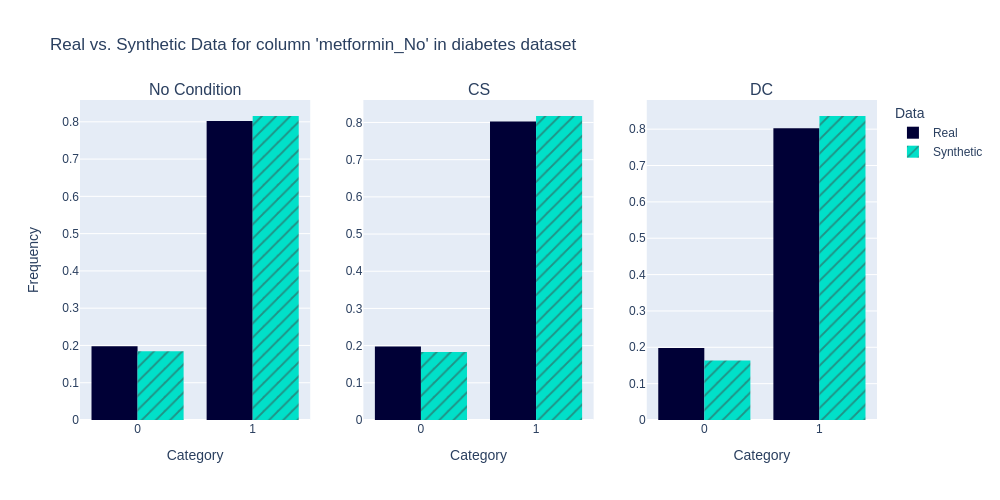


Figure A3-74. Data quality of “metformin no” column in the diabetes data.


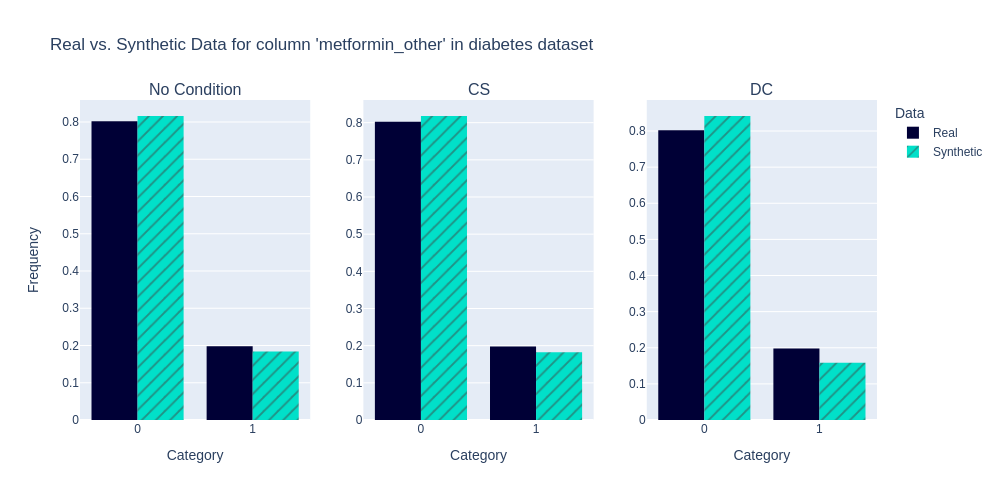


Figure A3-75. Data quality of “metformin other” column in the diabetes data.


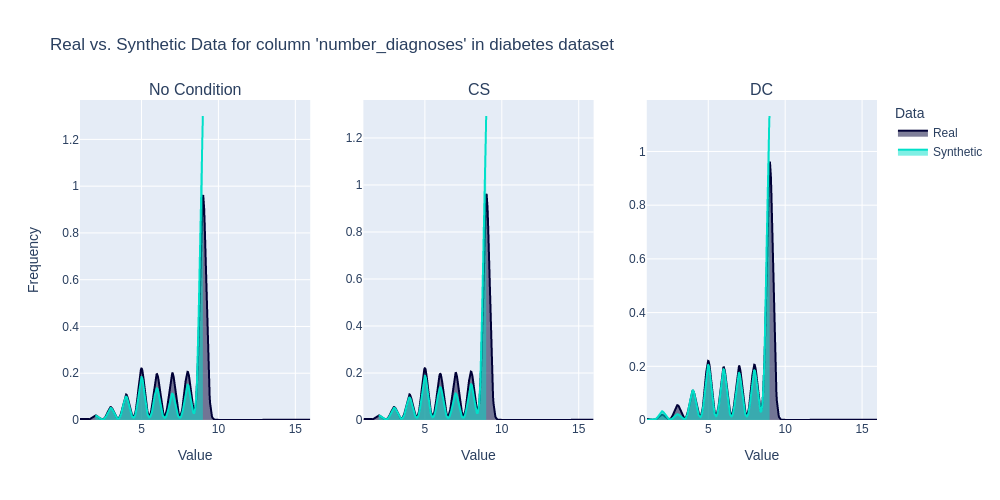


Figure A3-76. Data quality of “number diagnoses” column in the diabetes data.


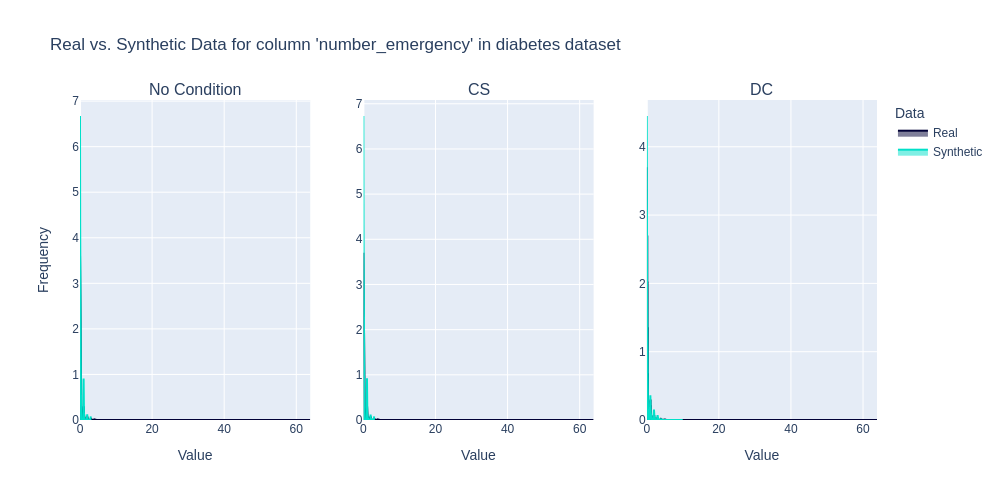


Figure A3-77. Data quality of “number emergency” column in the diabetes data.


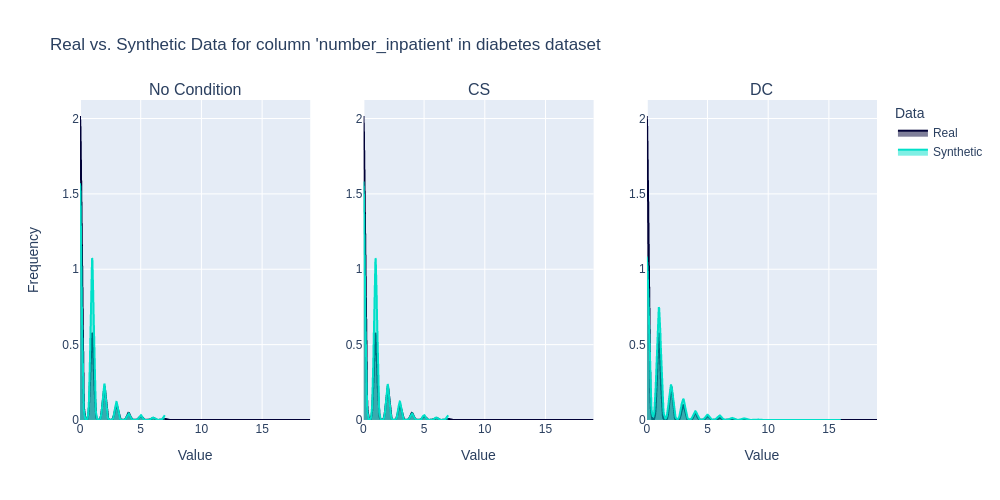


Figure A3-78. Data quality of “number inpatient” column in the diabetes data.


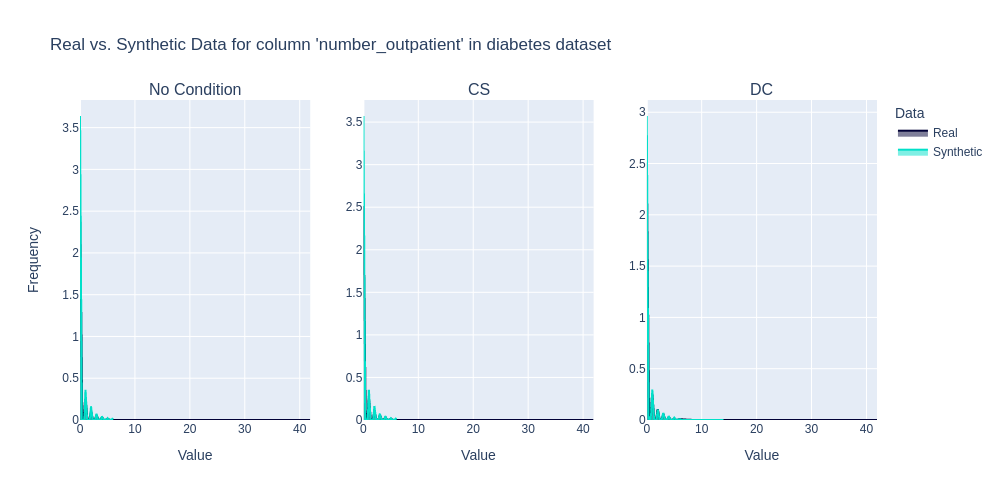


Figure A3-79. Data quality of “number outpatient” column in the diabetes data.


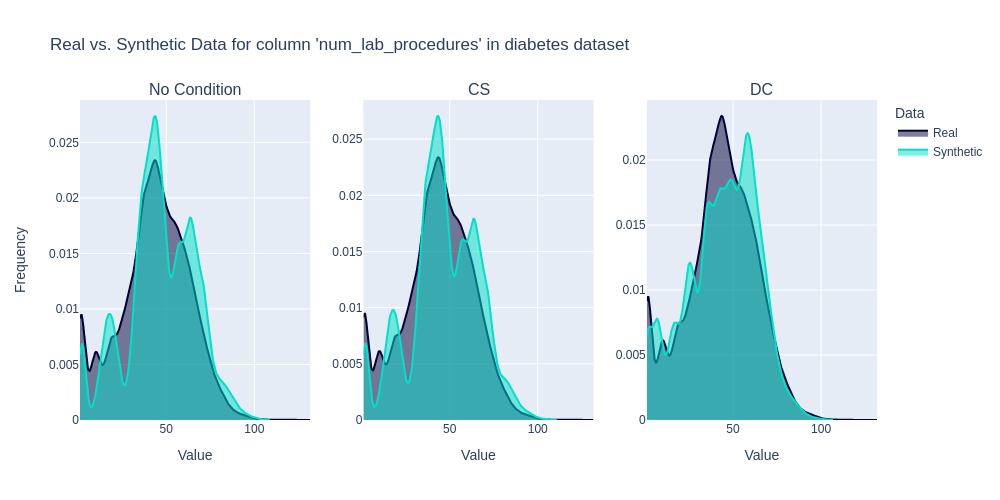


Figure A3-80. Data quality of “num lab procedures” column in the diabetes data.


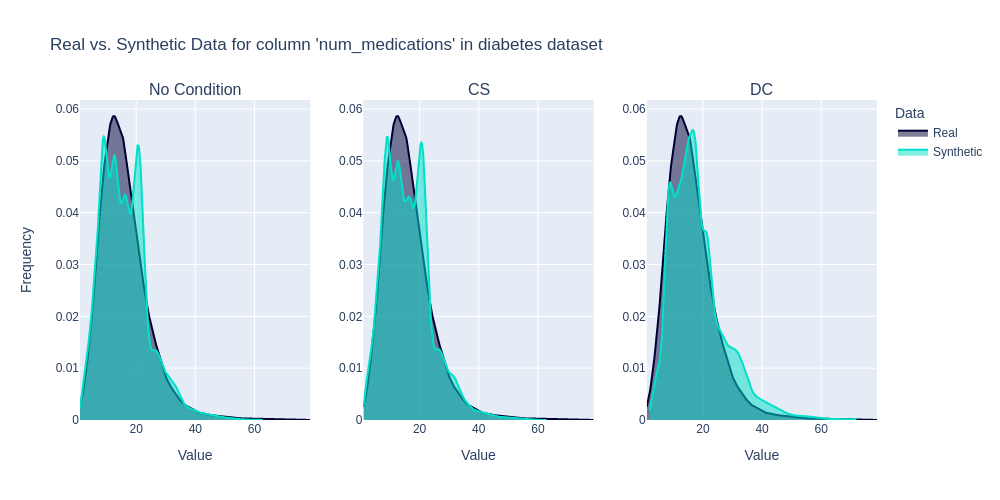


Figure A3-81. Data quality of “num medications” column in the diabetes data.


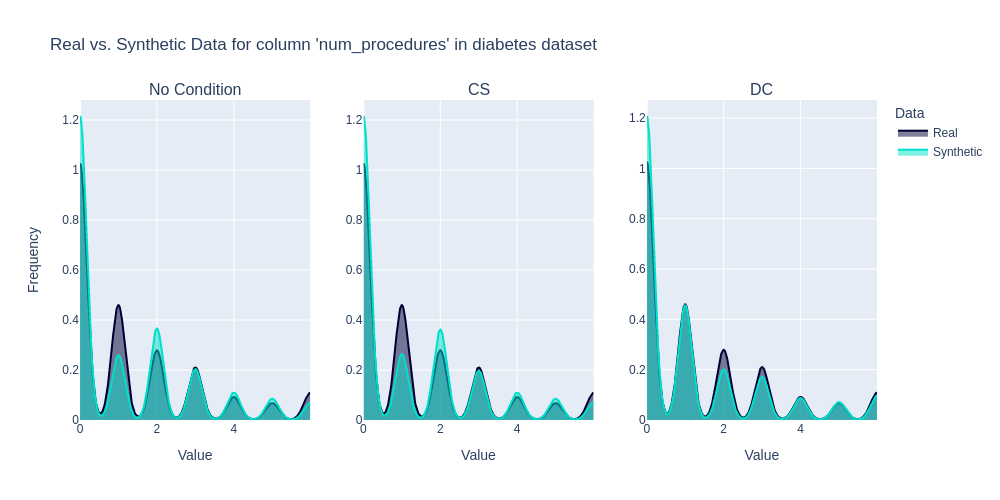


Figure A3-82. Data quality of “num procedures” column in the diabetes data.


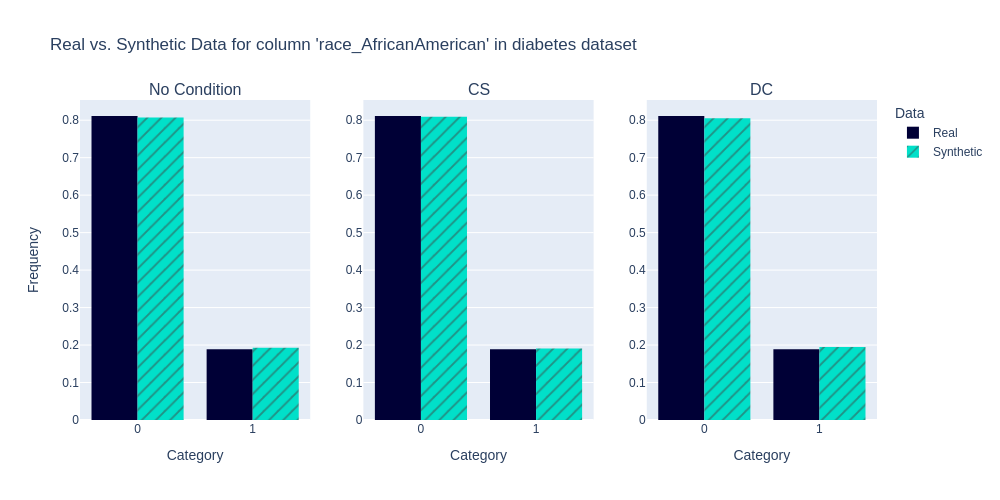


Figure A3-83. Data quality of “race african american” column in the diabetes data.


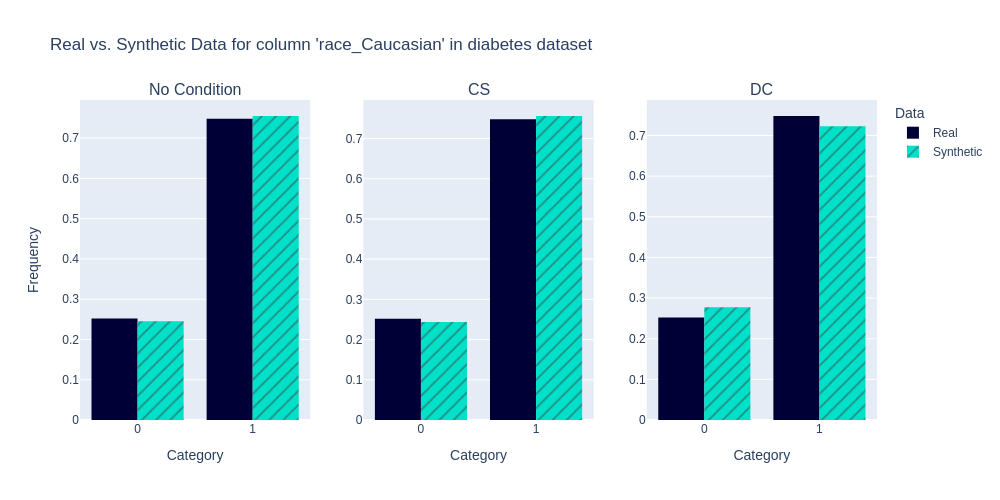


Figure A3-84. Data quality of “race caucasian” column in the diabetes data.


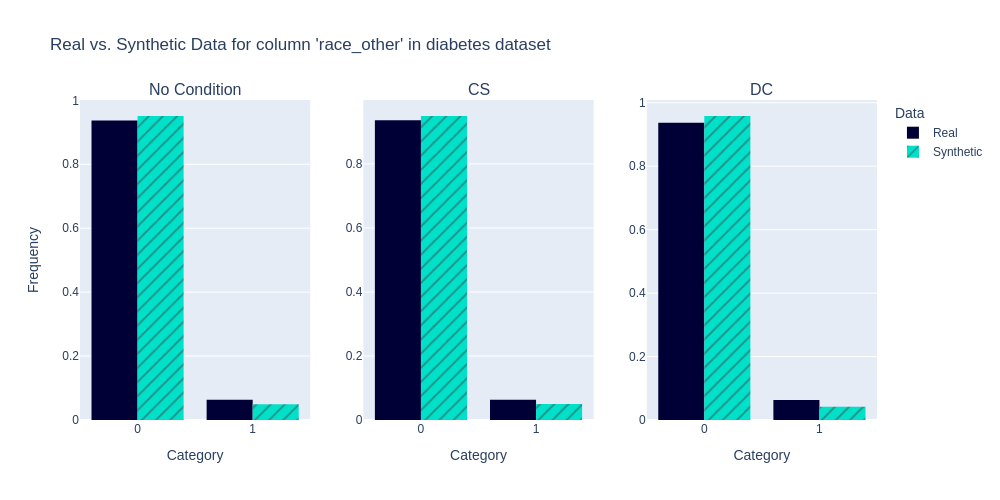


Figure A3-85. Data quality of “race other” column in the diabetes data.


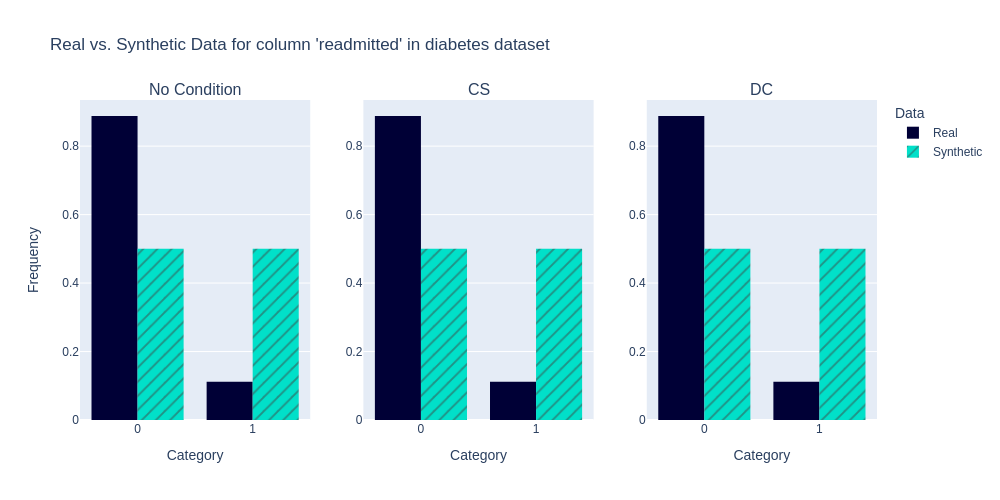


Figure A3-86. Data quality of “readmitted” column in the diabetes data.


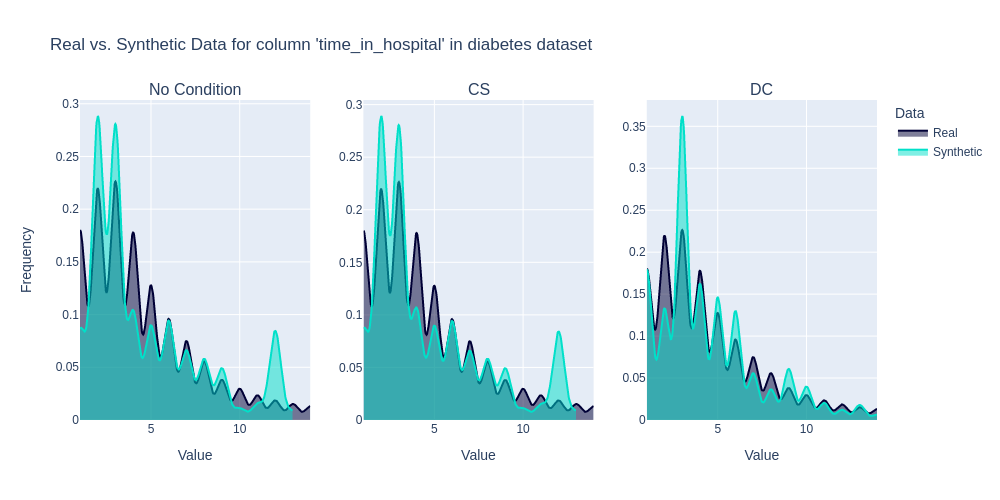


Figure A3-87. Data quality of “time in hospital” column in the diabetes data.
