# Supplementary material for: Synthetic Tabular Data Based on Generative Adversarial Networks in Health Care: Generation and Validation Using the Divide-and-Conquer Strategy
Source: JMIR Med Inform. 2023 Nov 24;11:e47859. doi: 10.2196/47859 (PMC10709788; doi:10.2196/47859)
Supplement: Multimedia Appendix 4 [file medinform_v11i1e47859_app4.docx]

**Multimedia Appendix 4**

In Figure A4-1, comparison between the AS-IS and TO-BE states, highlighting the differences between CS (conditional sampling) and DC (divide and conquer) training strategies.


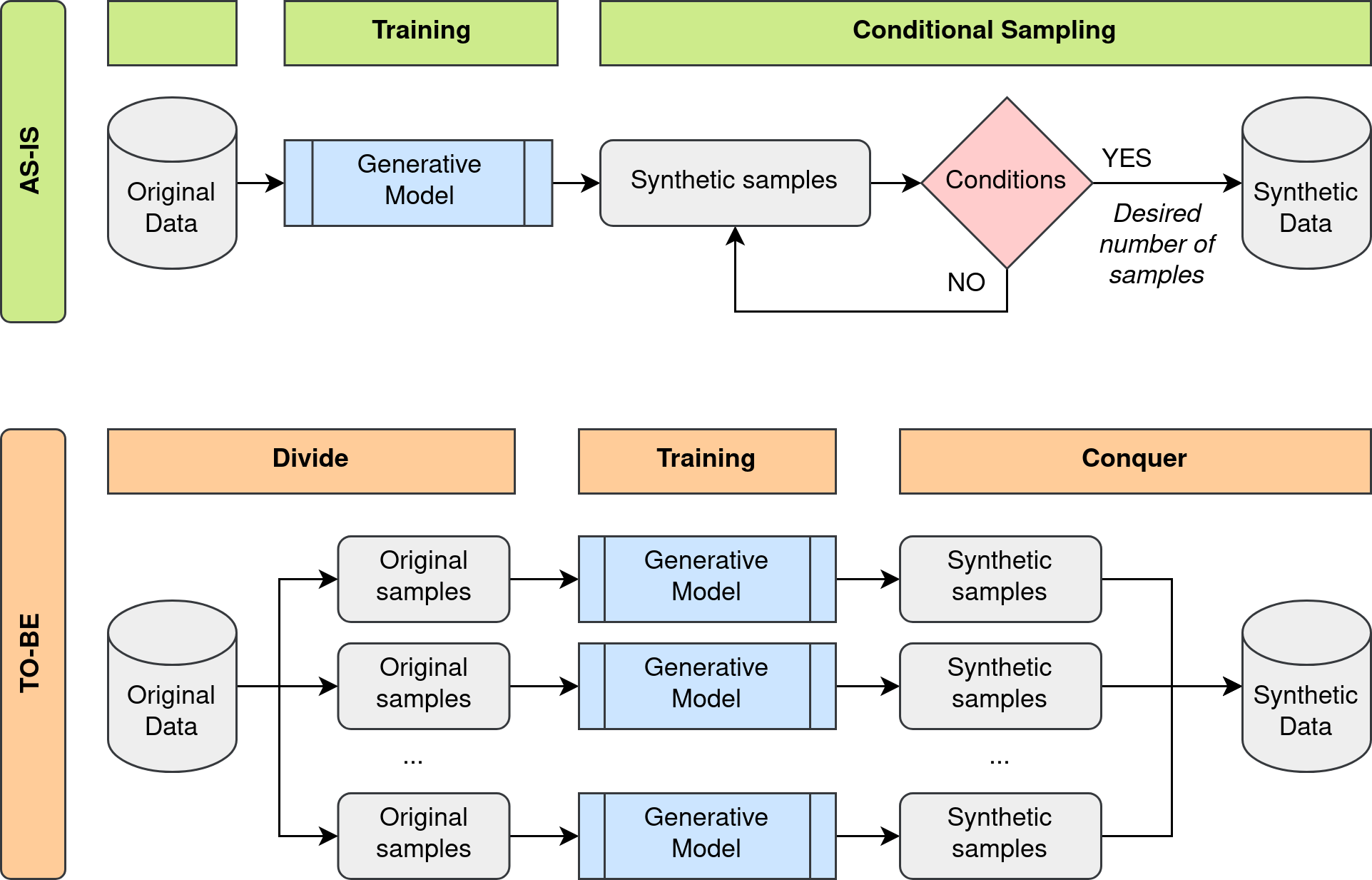


Figure A4-1. AS-IS and TO-BE.
