# Supplementary material for: Synthetic Tabular Data Based on Generative Adversarial Networks in Health Care: Generation and Validation Using the Divide-and-Conquer Strategy
Source: JMIR Med Inform. 2023 Nov 24;11:e47859. doi: 10.2196/47859 (PMC10709788; doi:10.2196/47859)
Supplement: Multimedia Appendix 5 [file medinform_v11i1e47859_app5.docx]

**Multimedia Appendix 5**

Figures A5-[1-48] demonstrates the impact of the number of epochs on performance measures such as AUC and F1-score.


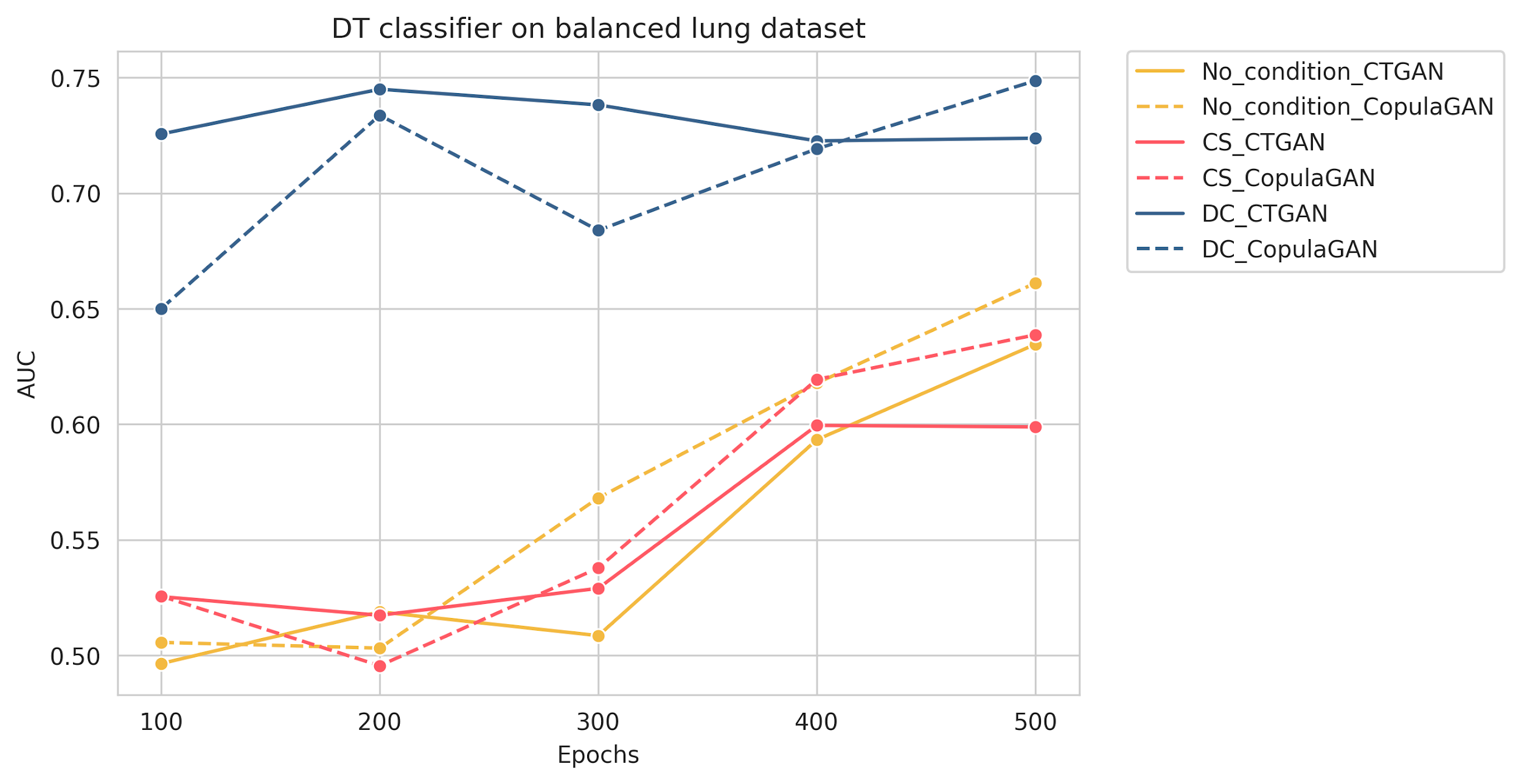


Figure A5-1. Effect of epoch on AUC in the balanced lung dataset using DT classifier.


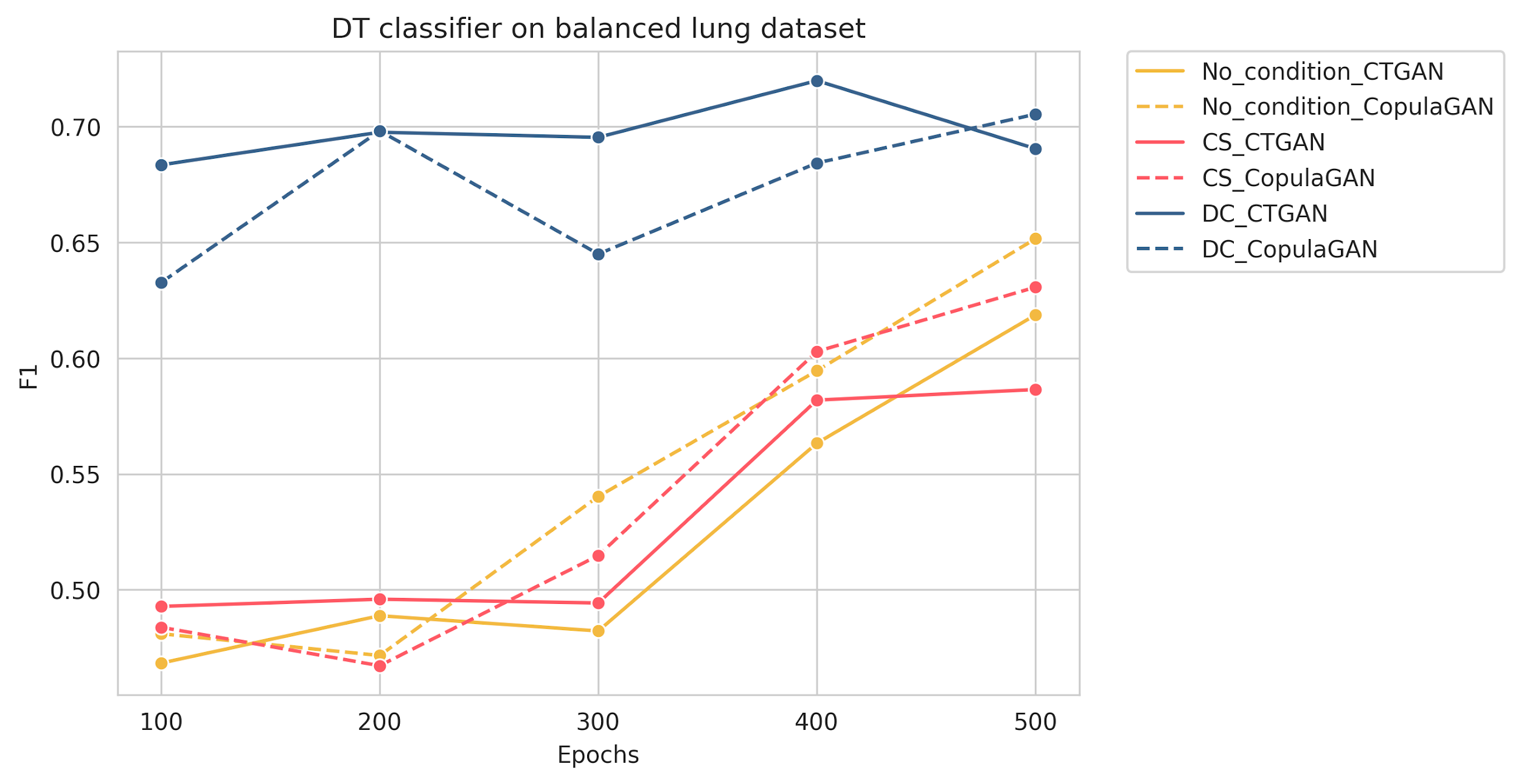


Figure A5-2. Effect of epoch on F1 in the balanced lung dataset using DT classifier.


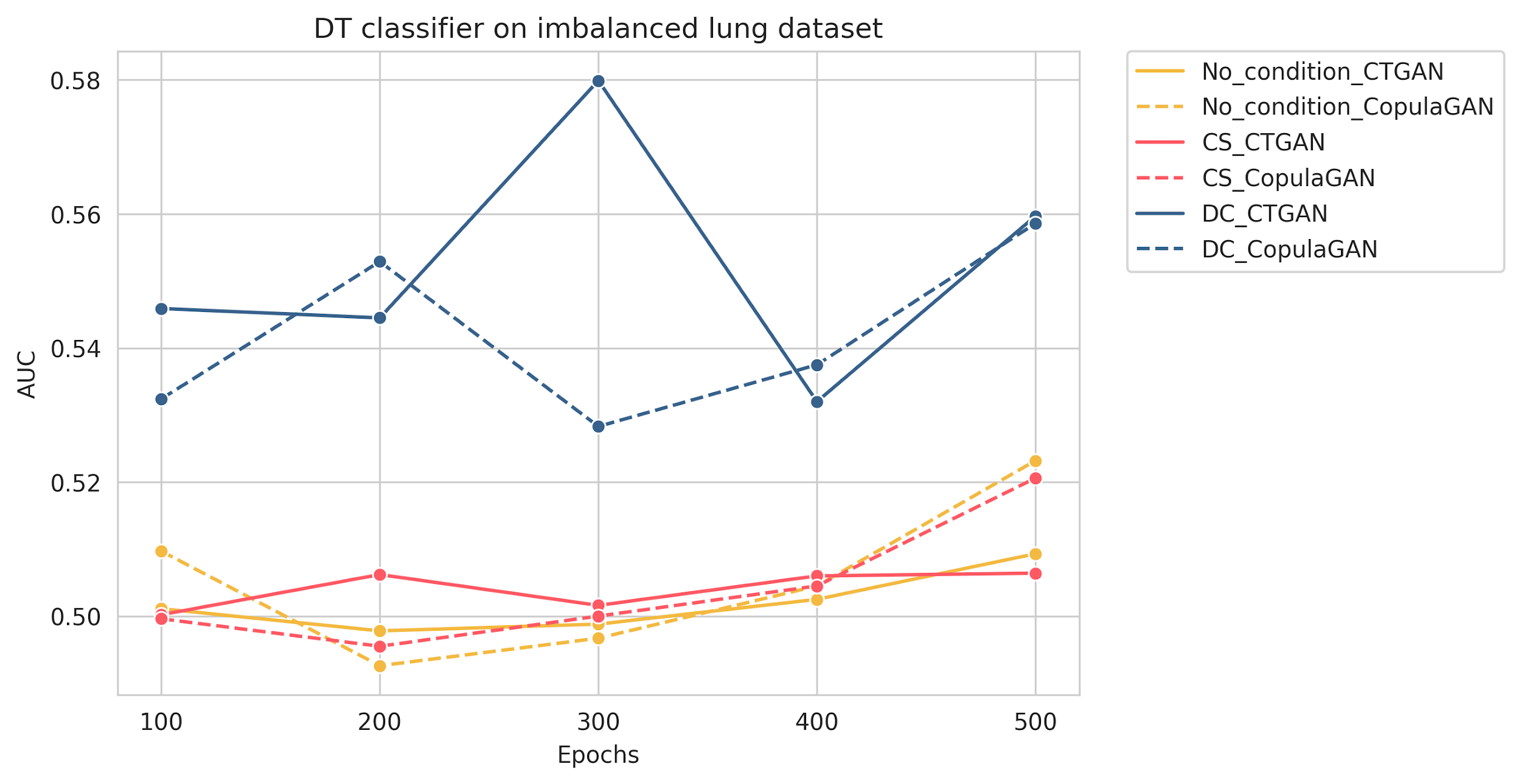


Figure A5-3. Effect of epoch on AUC in the imbalanced lung dataset using DT classifier.


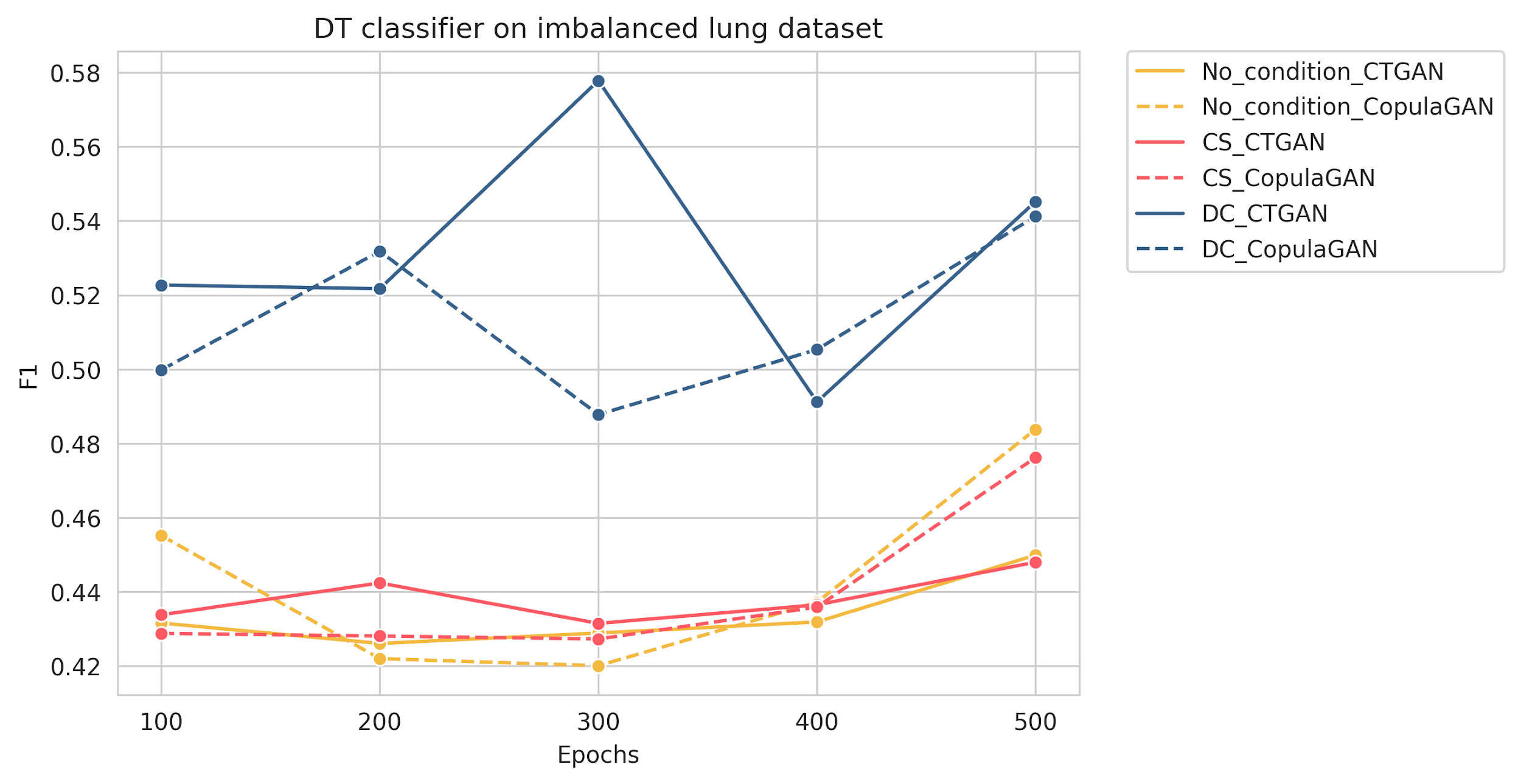


Figure A5-4. Effect of epoch on F1 in the imbalanced lung dataset using DT classifier.


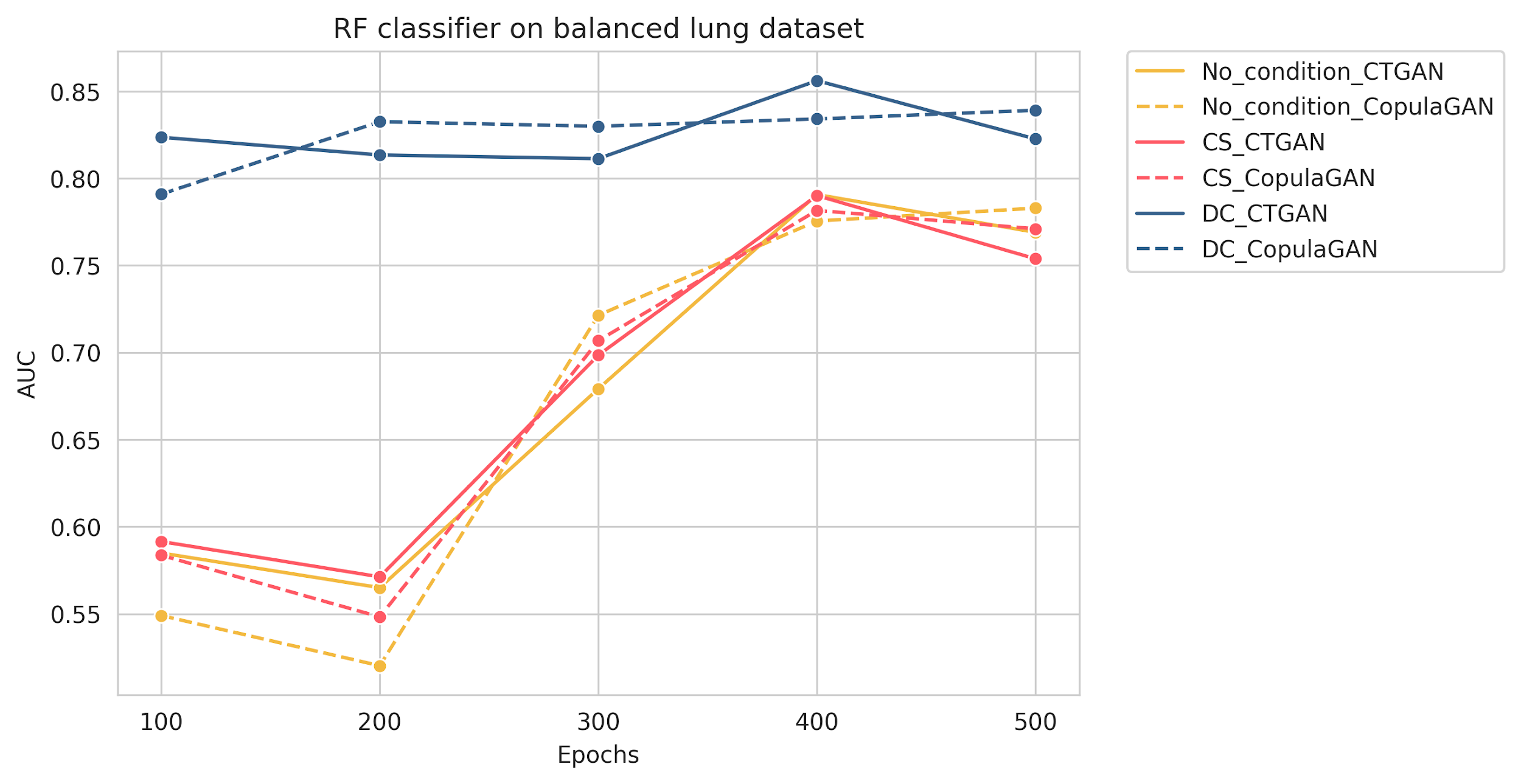


Figure A5-5. Effect of epoch on AUC in the balanced lung dataset using RF classifier.


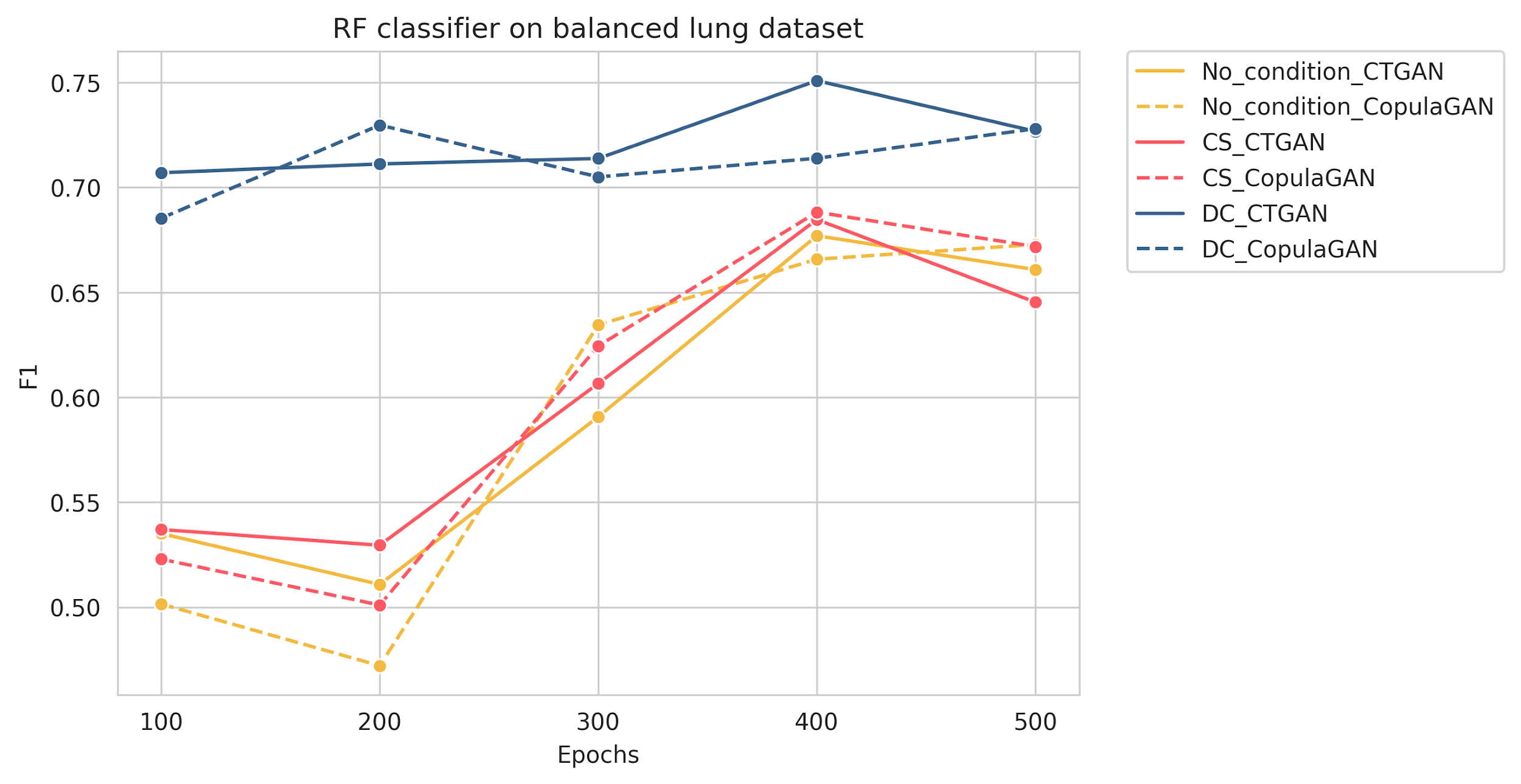


Figure A5-6. Effect of epoch on F1 in the balanced lung dataset using RF classifier.


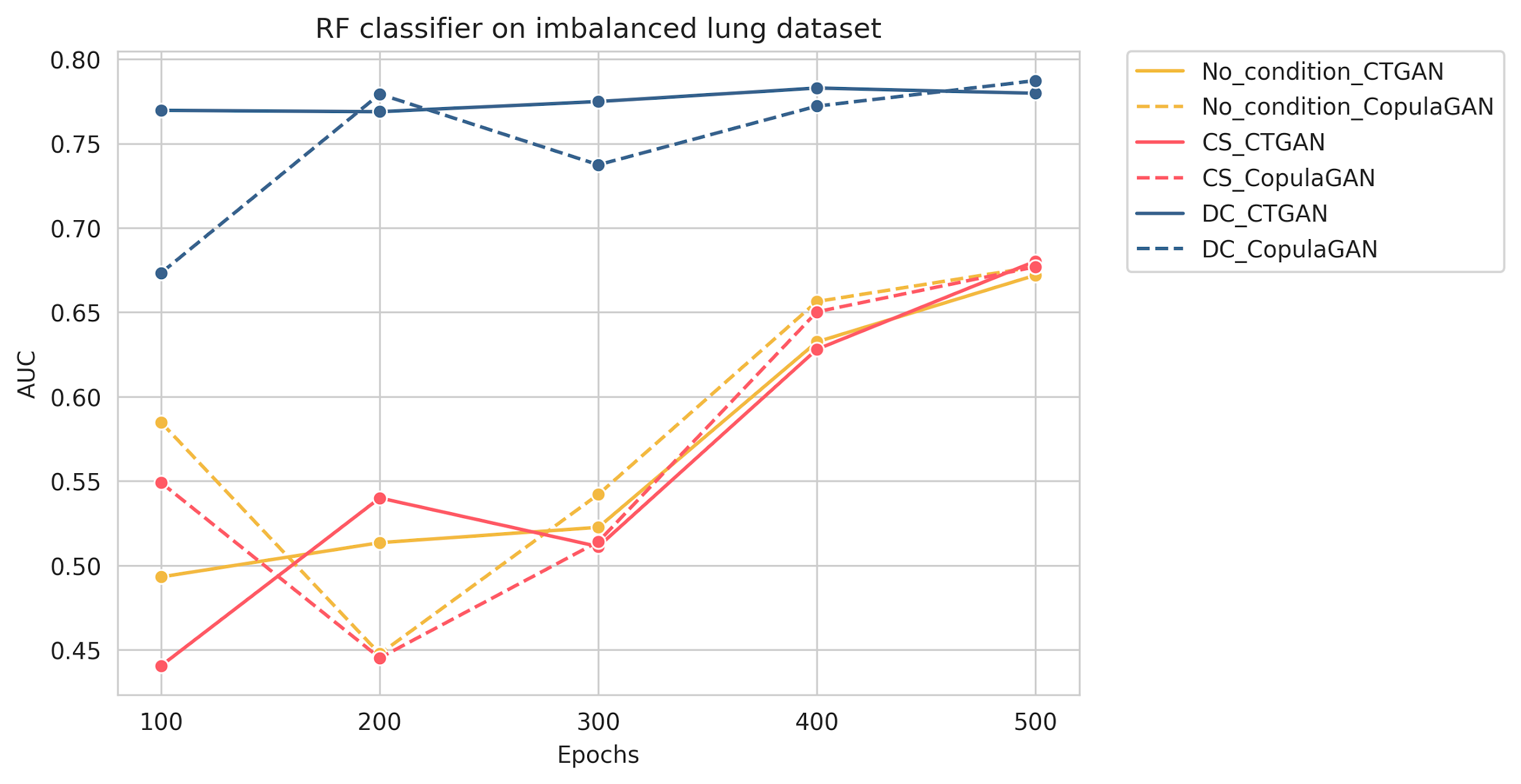


Figure A5-7. Effect of epoch on AUC in the imbalanced lung dataset using RF classifier.


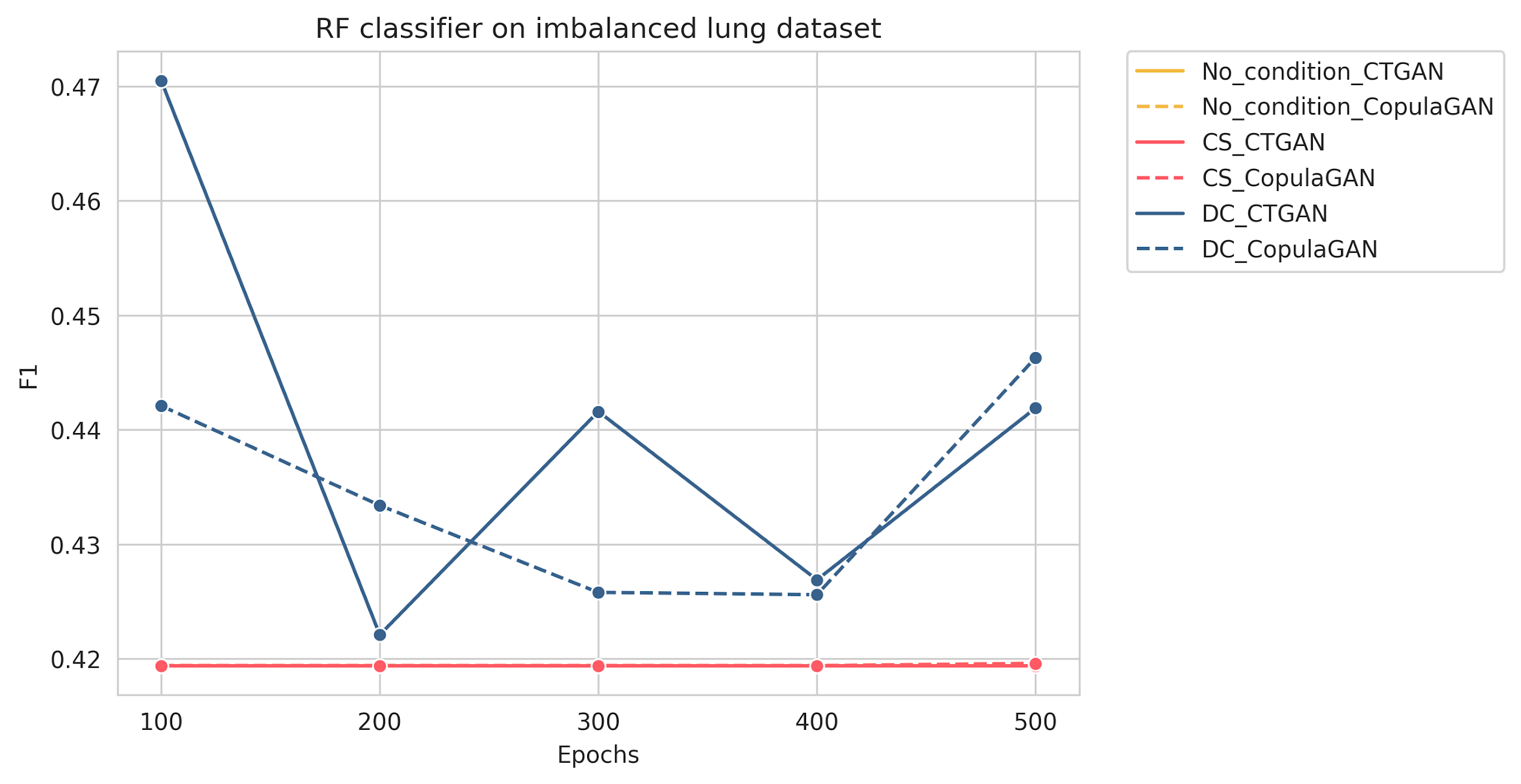


Figure A5-8. Effect of epoch on F1 in the imbalanced lung dataset using RF classifier.


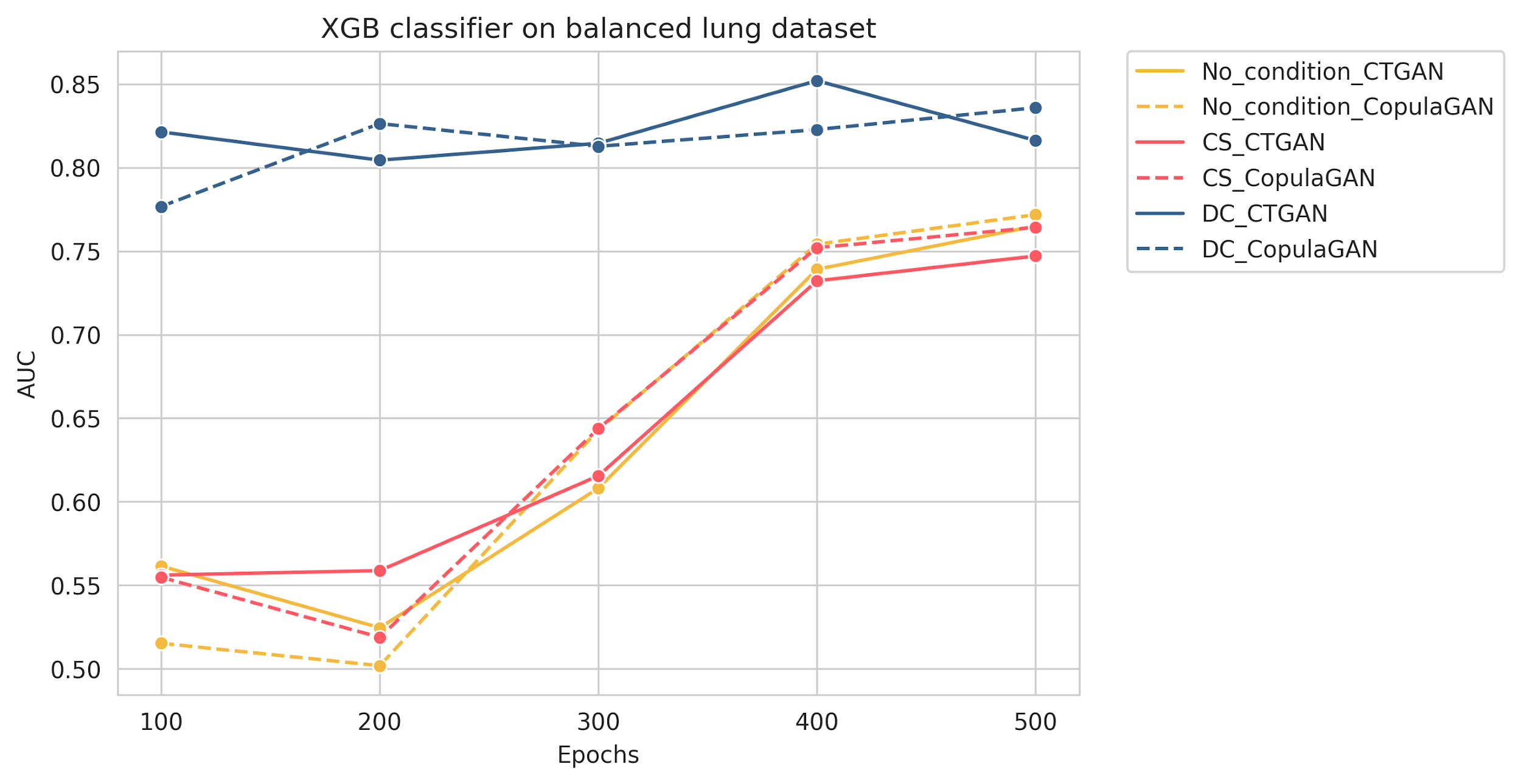


Figure A5-9. Effect of epoch on AUC in the balanced lung dataset using XGB classifier.


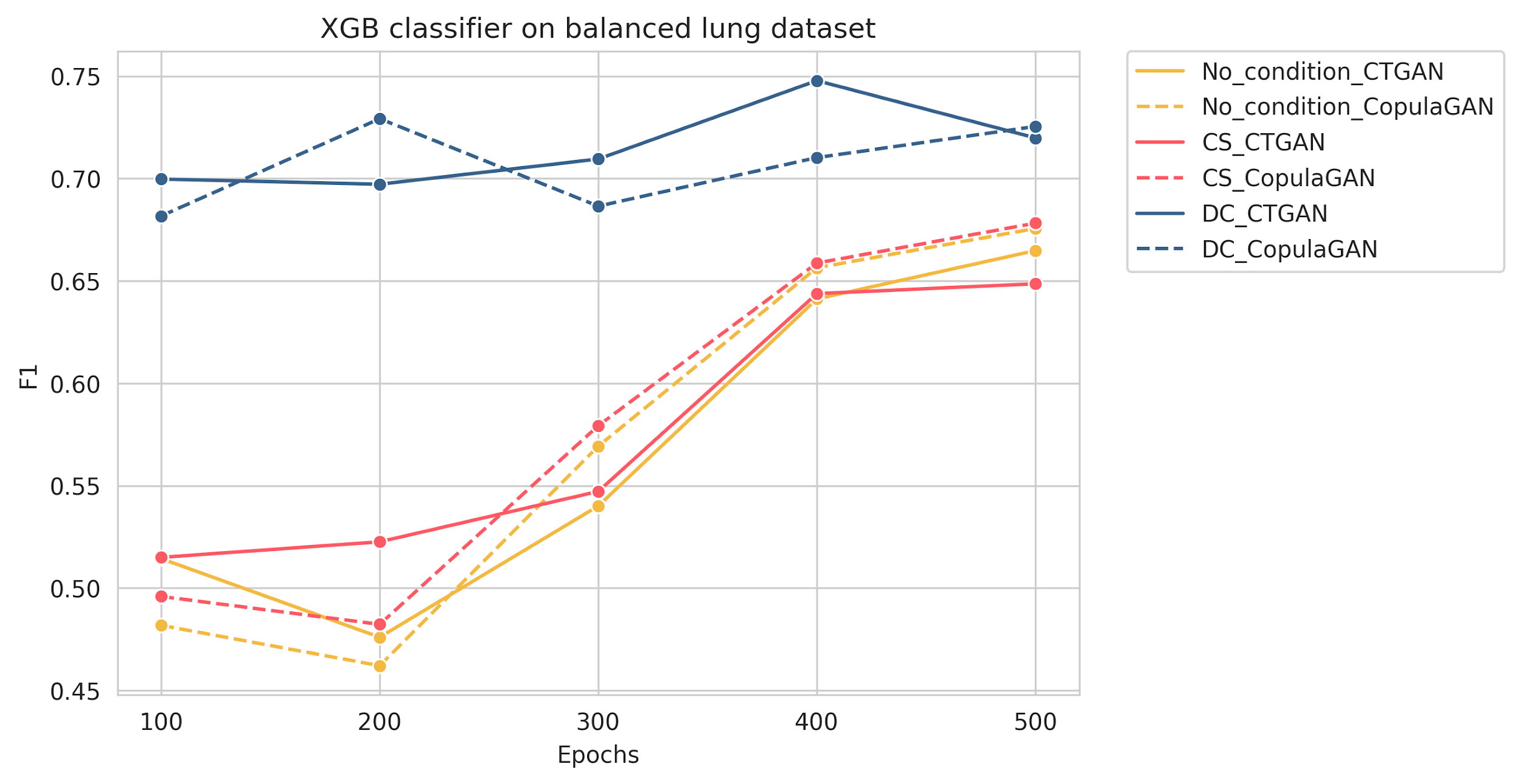


Figure A5-10. Effect of epoch on F1 in the balanced lung dataset using XGB classifier.


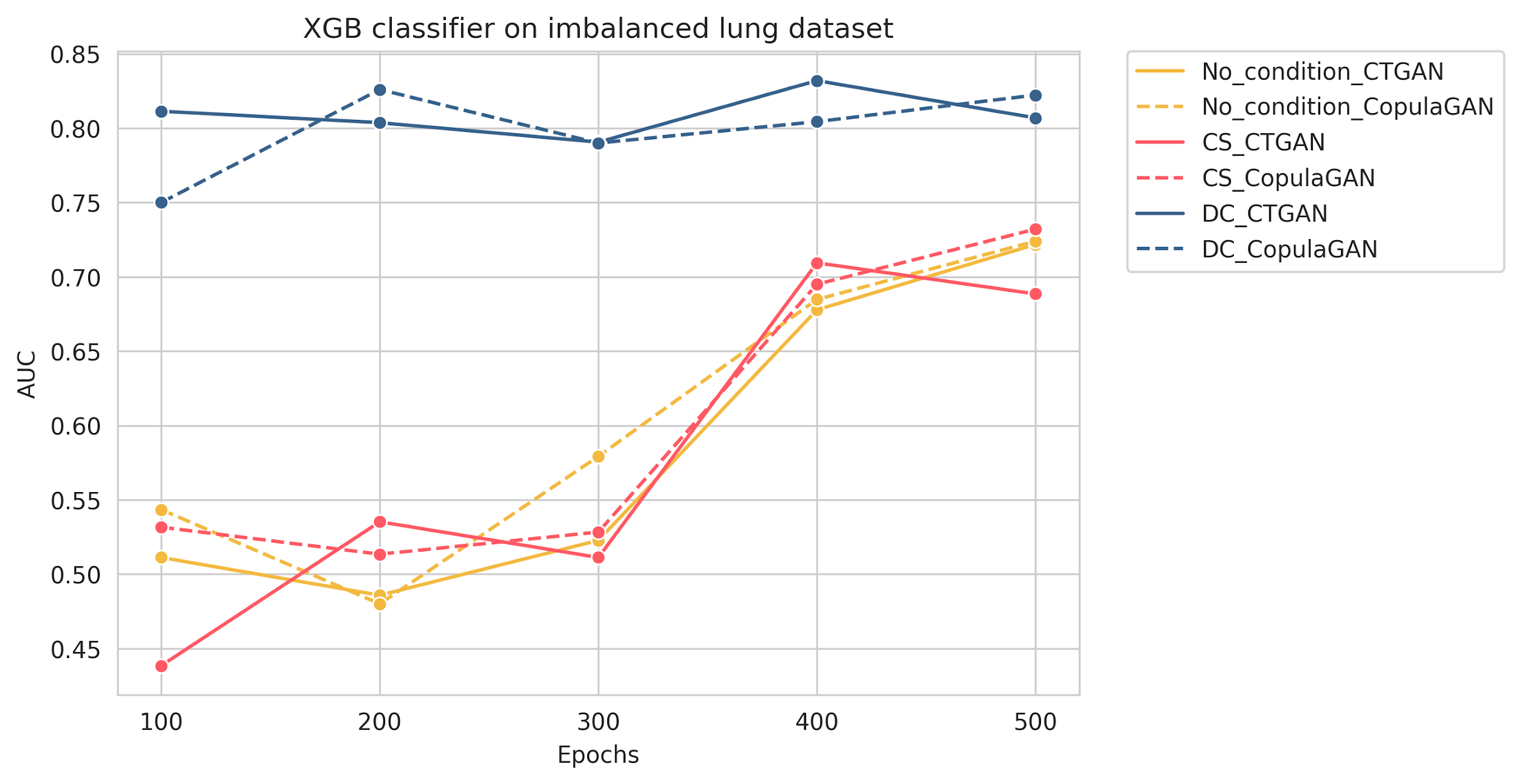


Figure A5-11. Effect of epoch on AUC in the imbalanced lung dataset using XGB classifier.


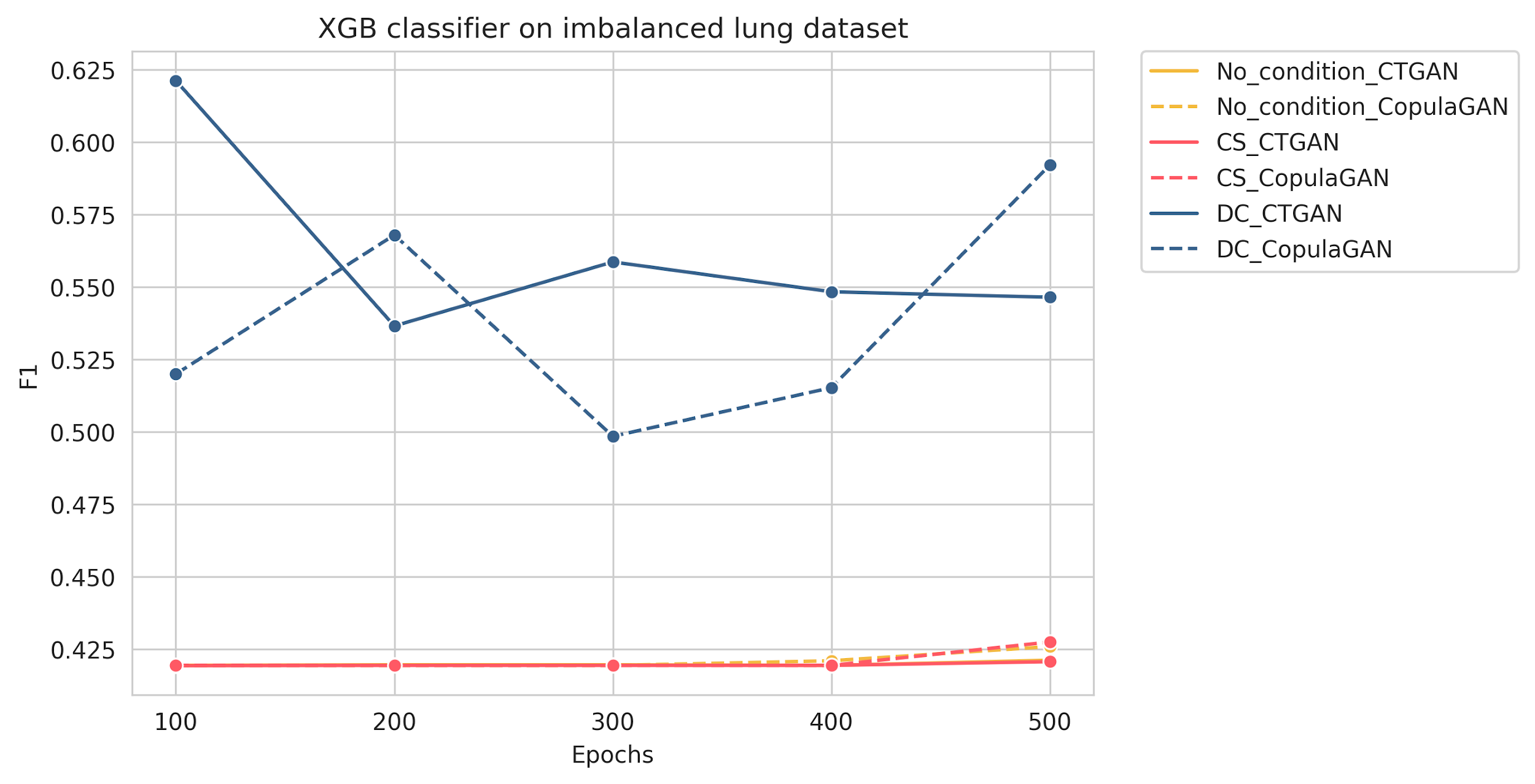


Figure A5-12. Effect of epoch on F1 in the imbalanced lung dataset using XGB classifier.


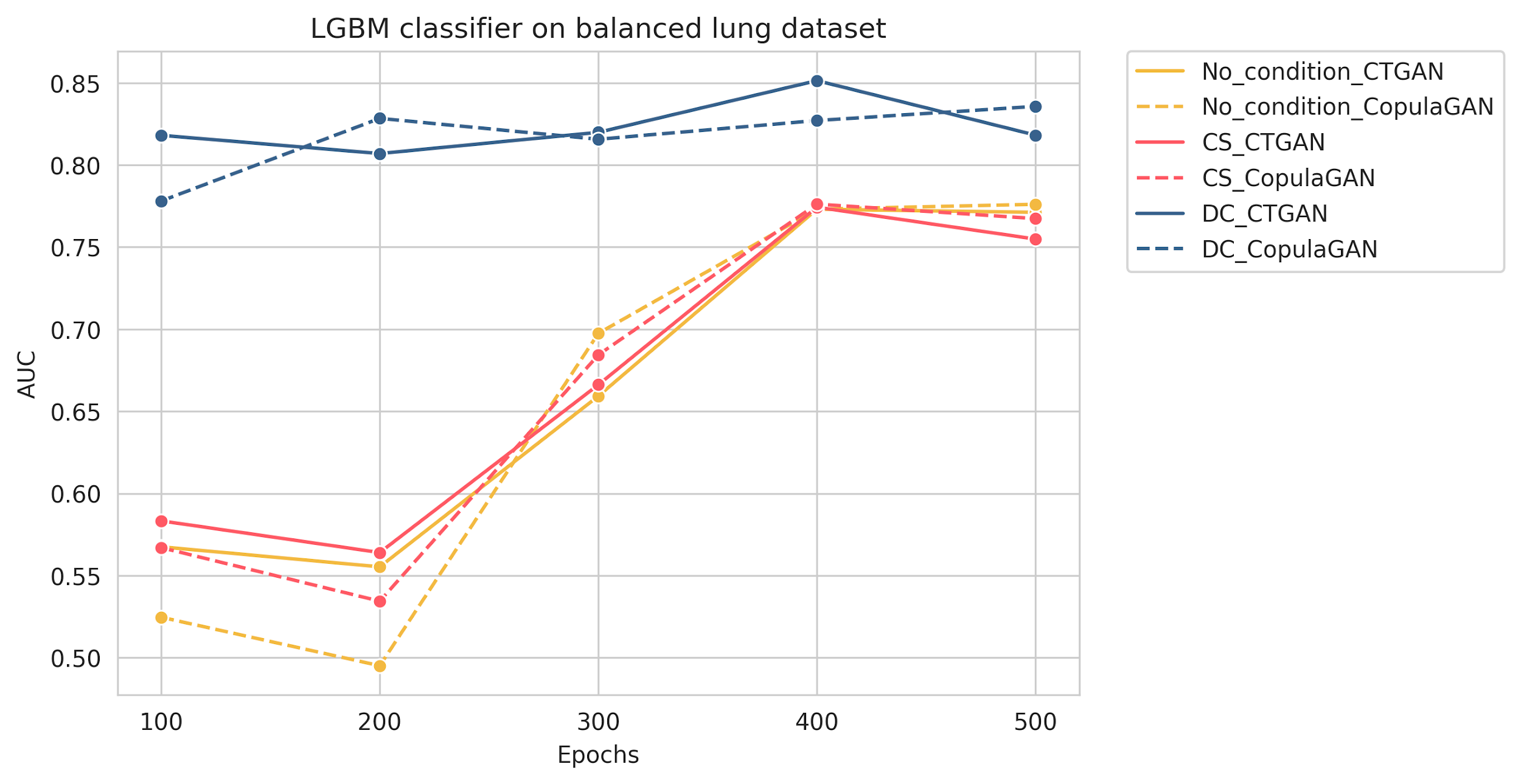


Figure A5-13. Effect of epoch on AUC in the balanced lung dataset using LGBM classifier.


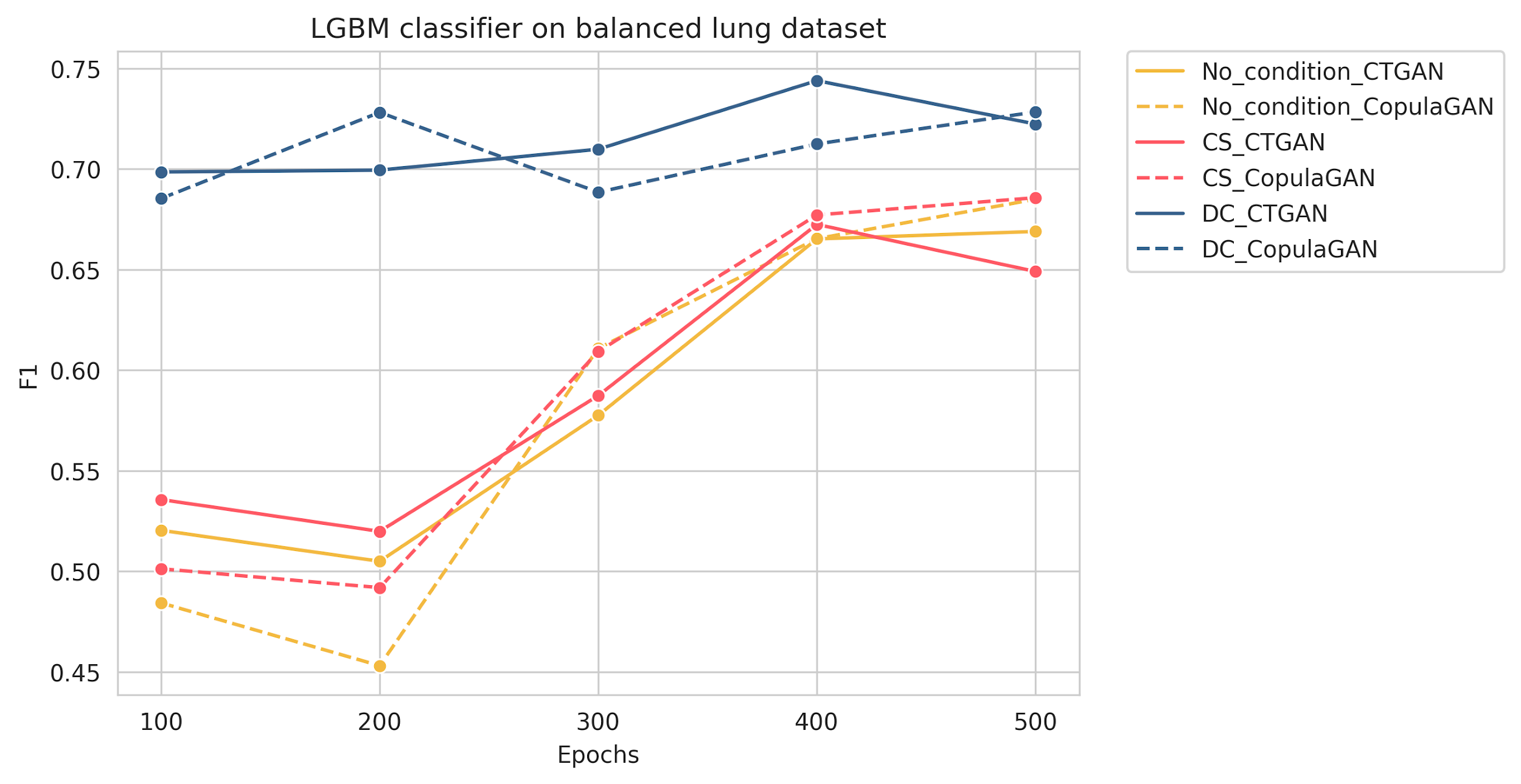


Figure A5-14. Effect of epoch on F1 in the balanced lung dataset using LGBM classifier.


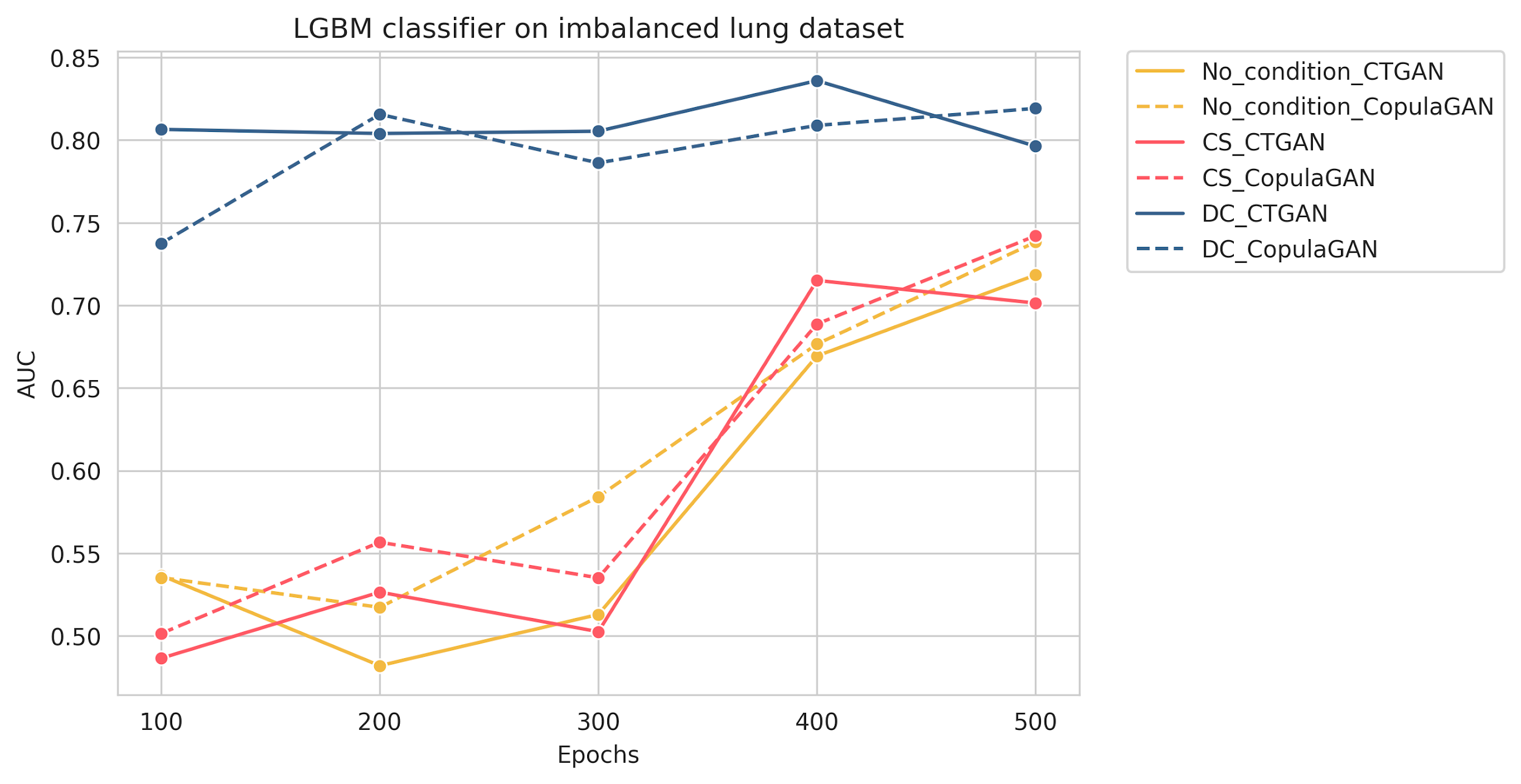


Figure A5-15. Effect of epoch on AUC in the imbalanced lung dataset using LGBM classifier.


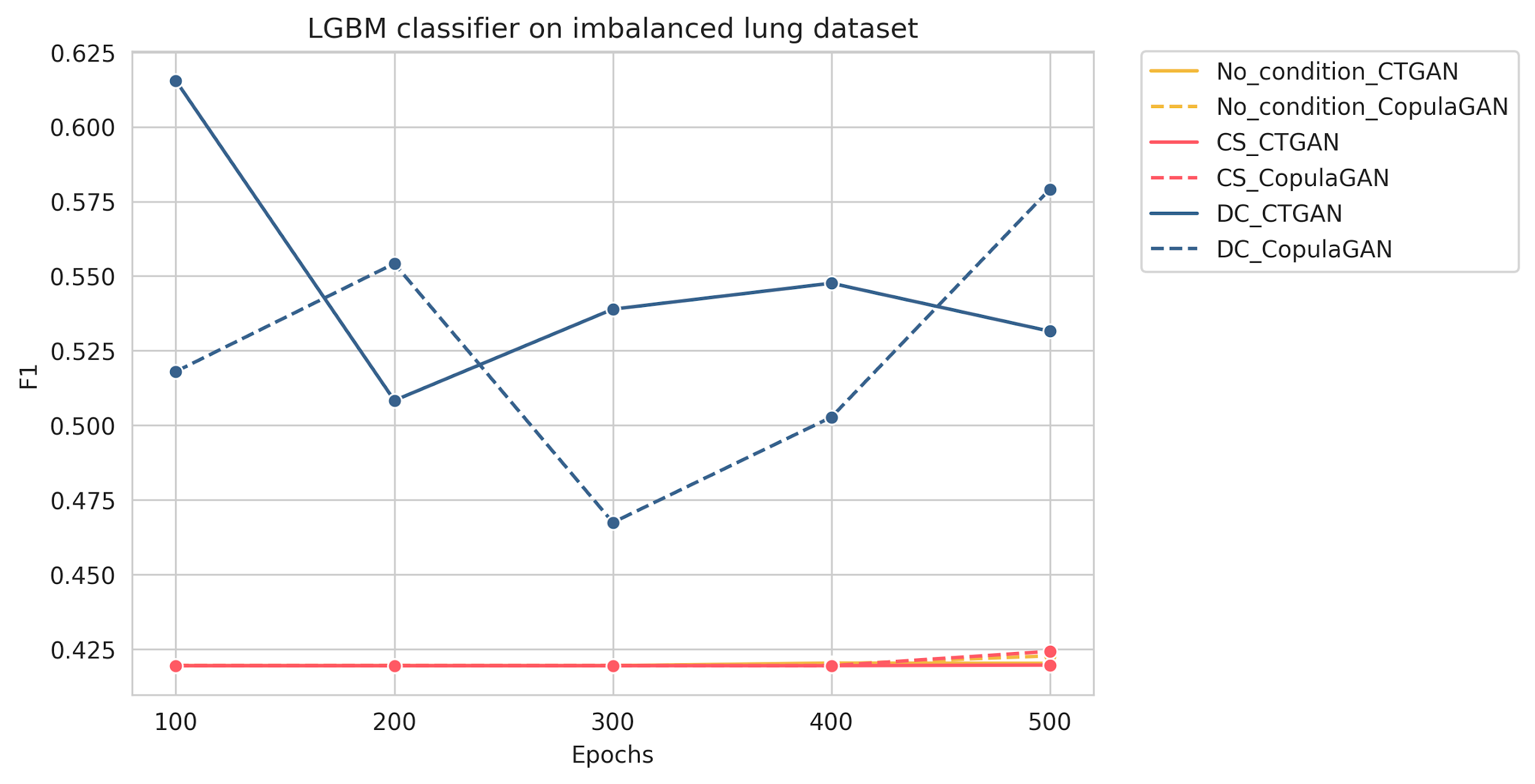


Figure A5-16. Effect of epoch on F1 in the imbalanced lung dataset using LGBM classifier.


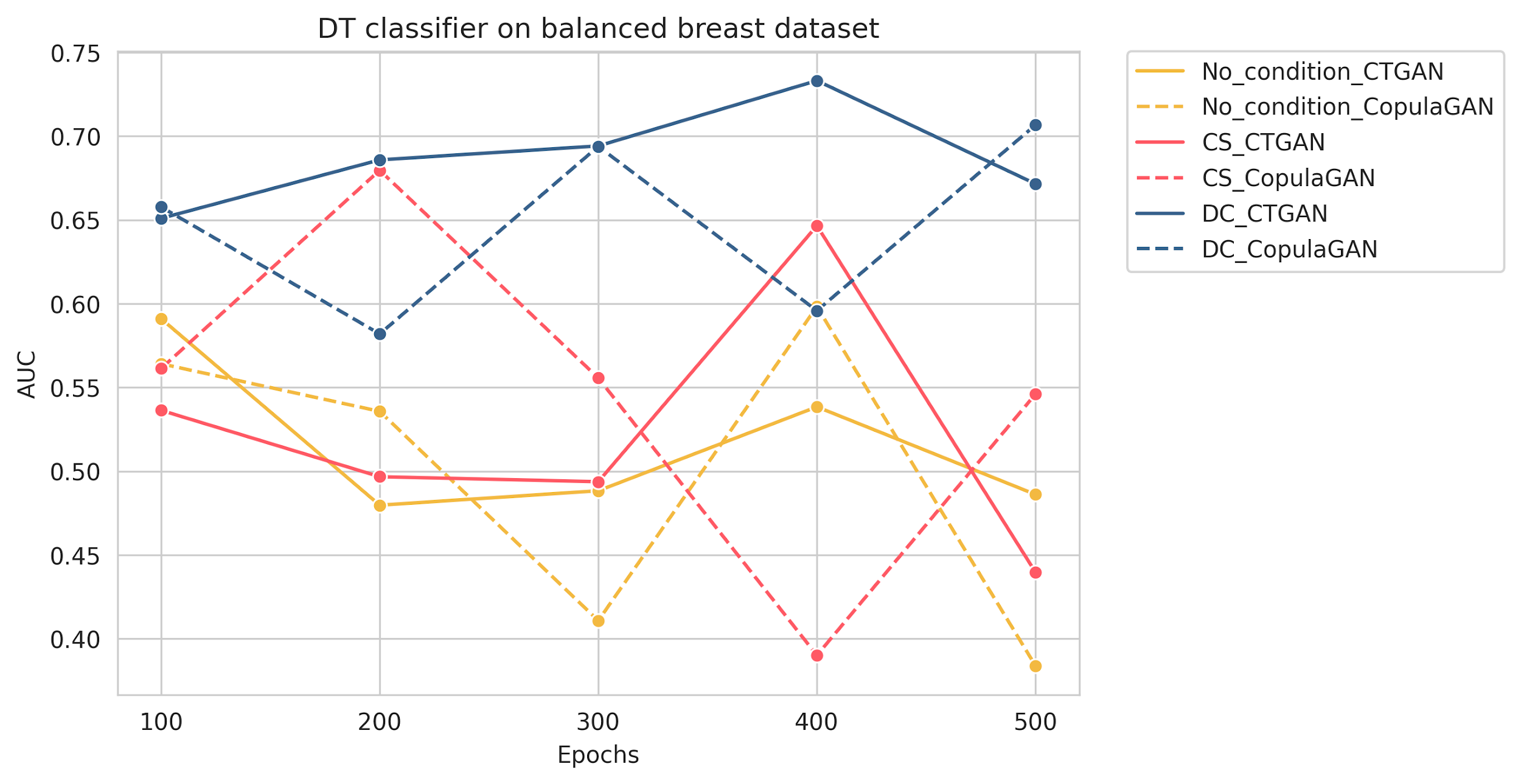


Figure A5-17. Effect of epoch on AUC in the balanced breast dataset using DT classifier.


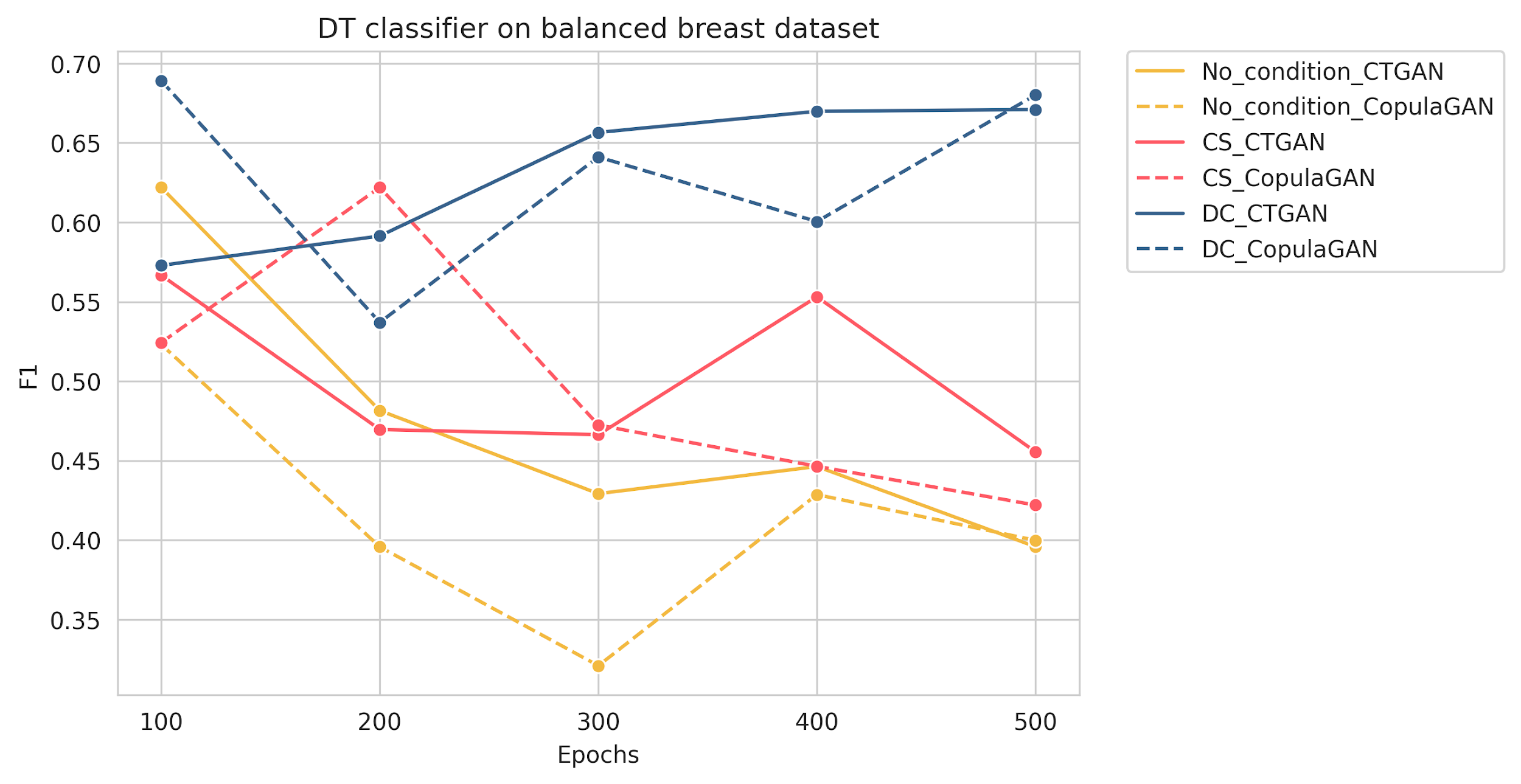


Figure A5-18. Effect of epoch on F1 in the balanced breast dataset using DT classifier.


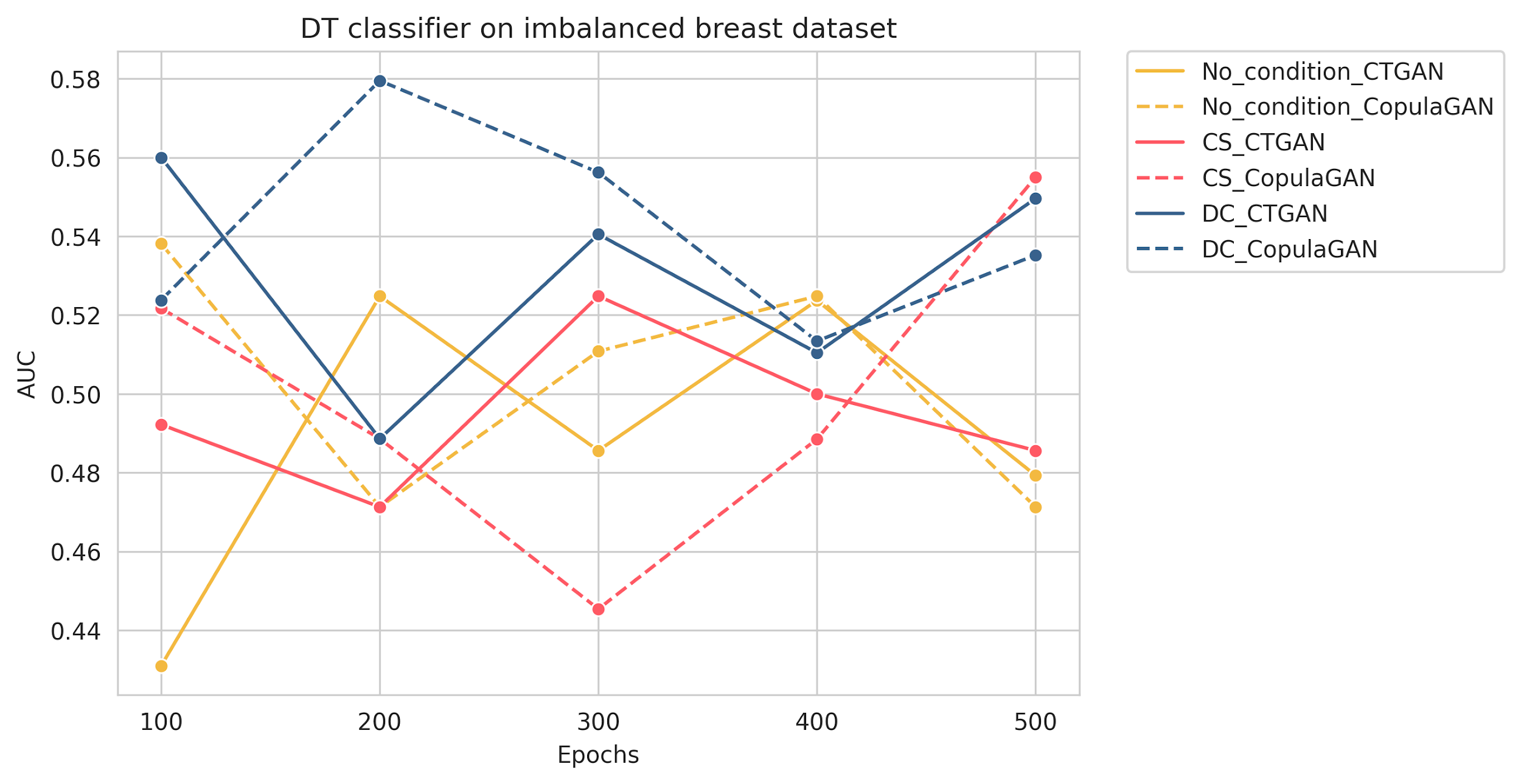


Figure A5-19. Effect of epoch on AUC in the imbalanced breast dataset using DT classifier.


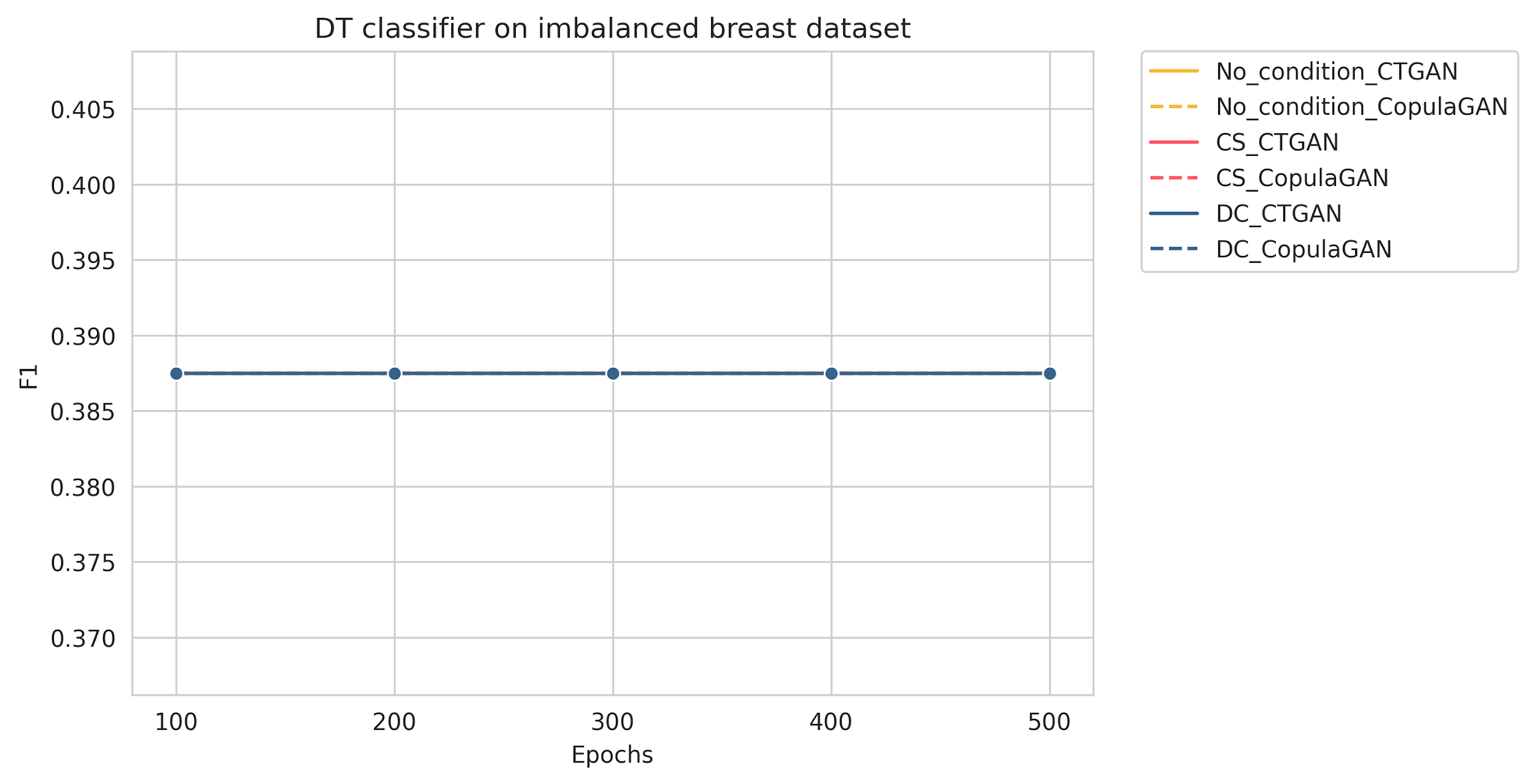


Figure A5-20. Effect of epoch on F1 in the imbalanced breast dataset using DT classifier.


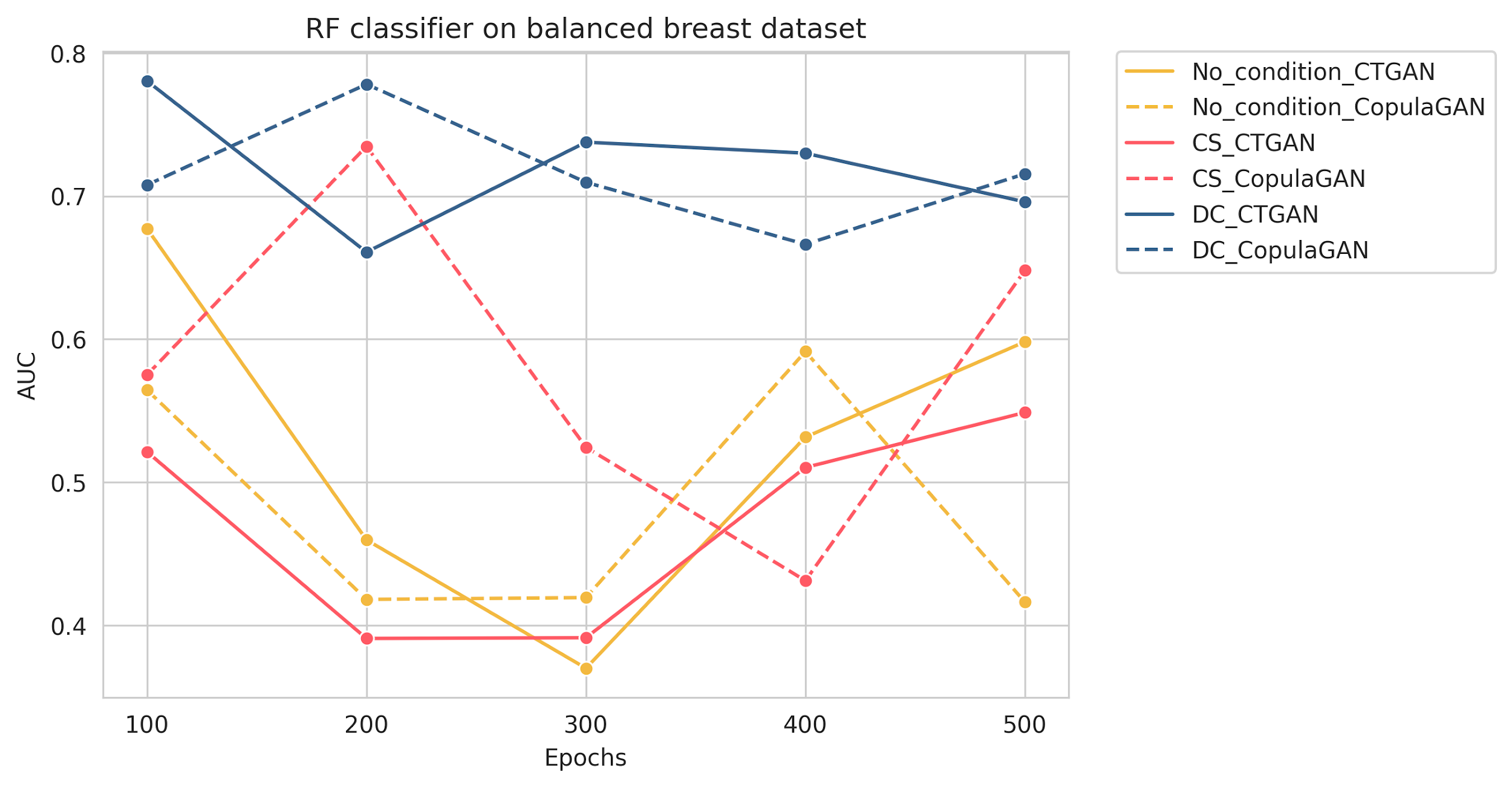


Figure A5-21. Effect of epoch on AUC in the balanced breast dataset using RF classifier.


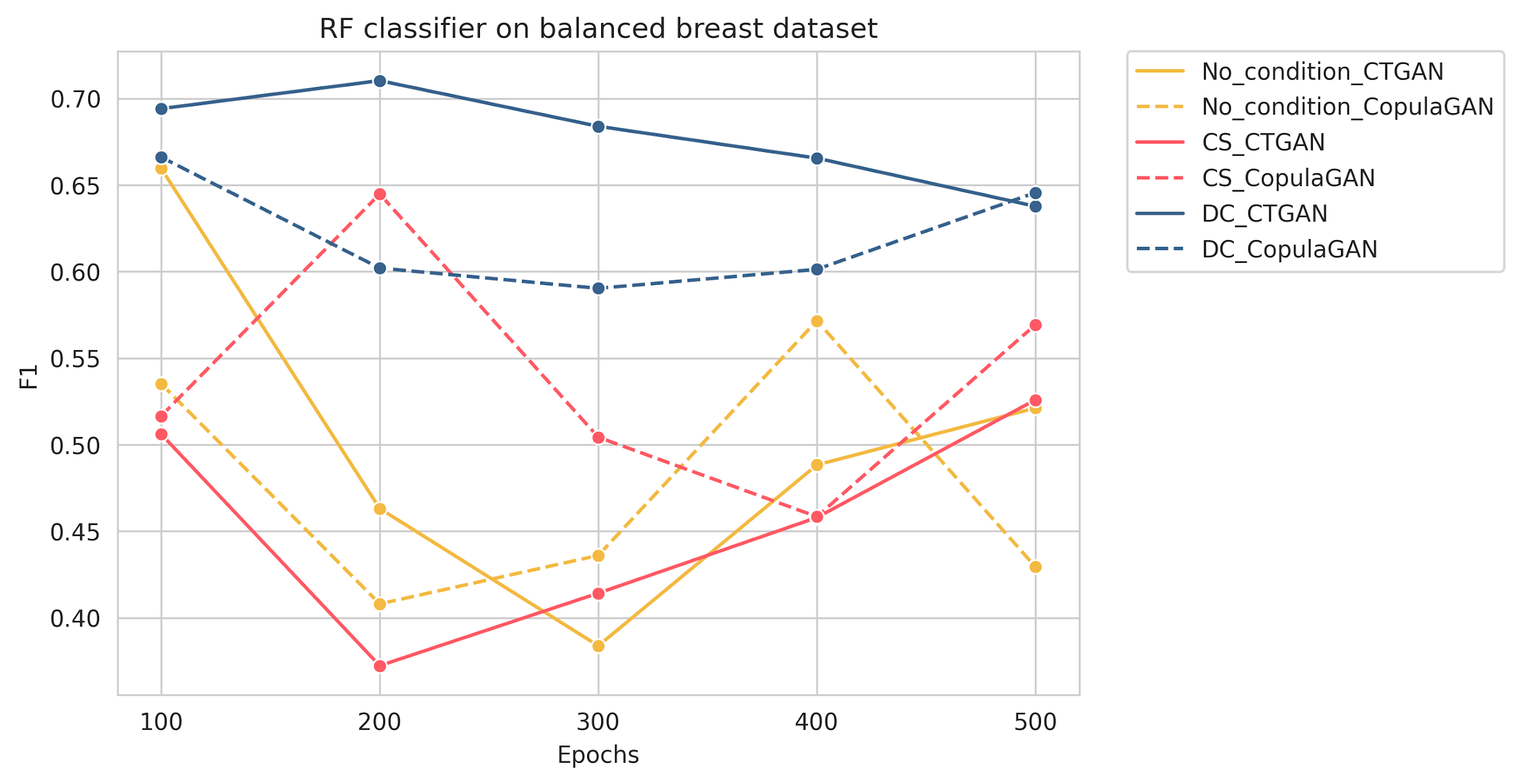


Figure A5-22. Effect of epoch on F1 in the balanced breast dataset using RF classifier.


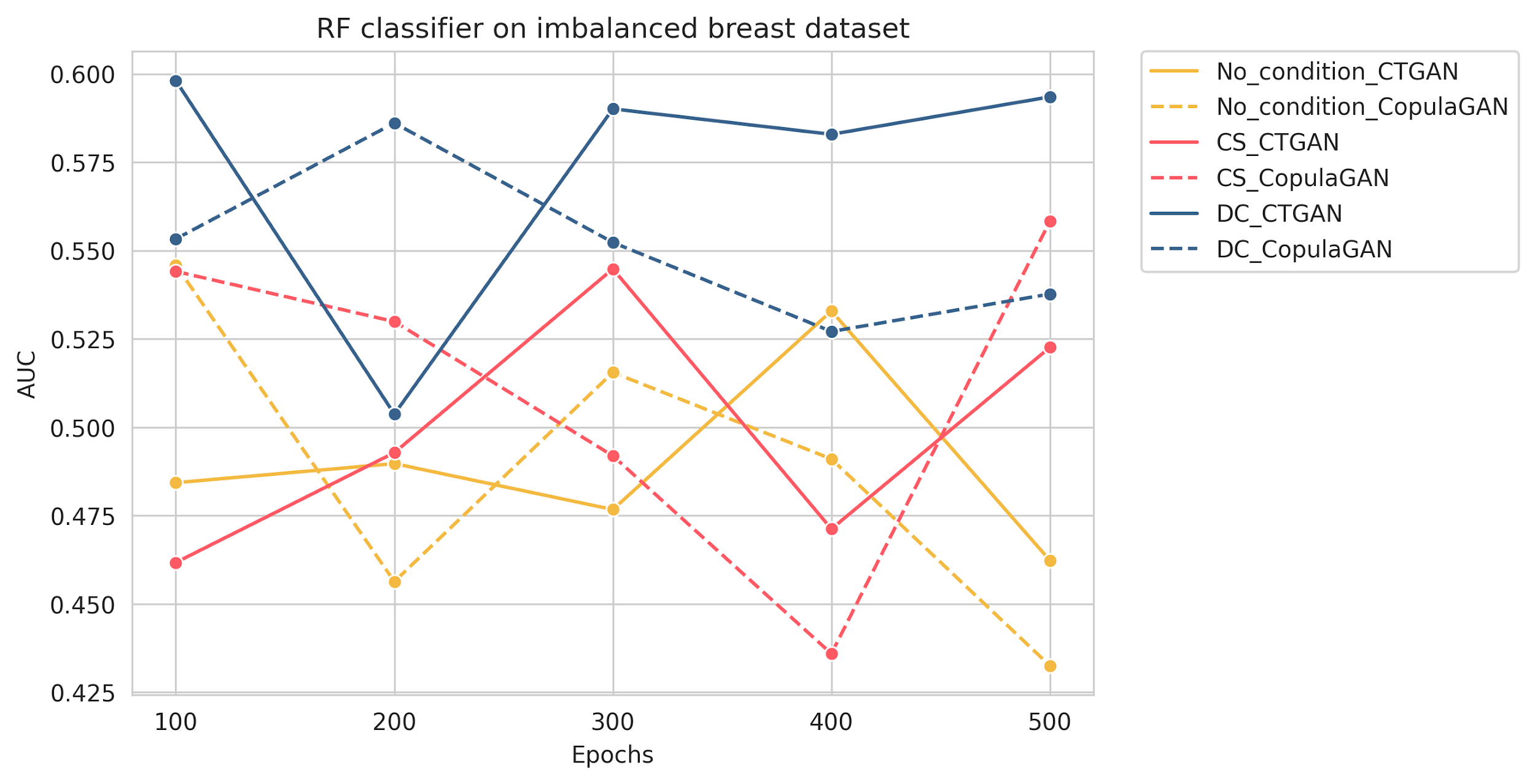


Figure A5-23. Effect of epoch on AUC in the imbalanced breast dataset using RF classifier.


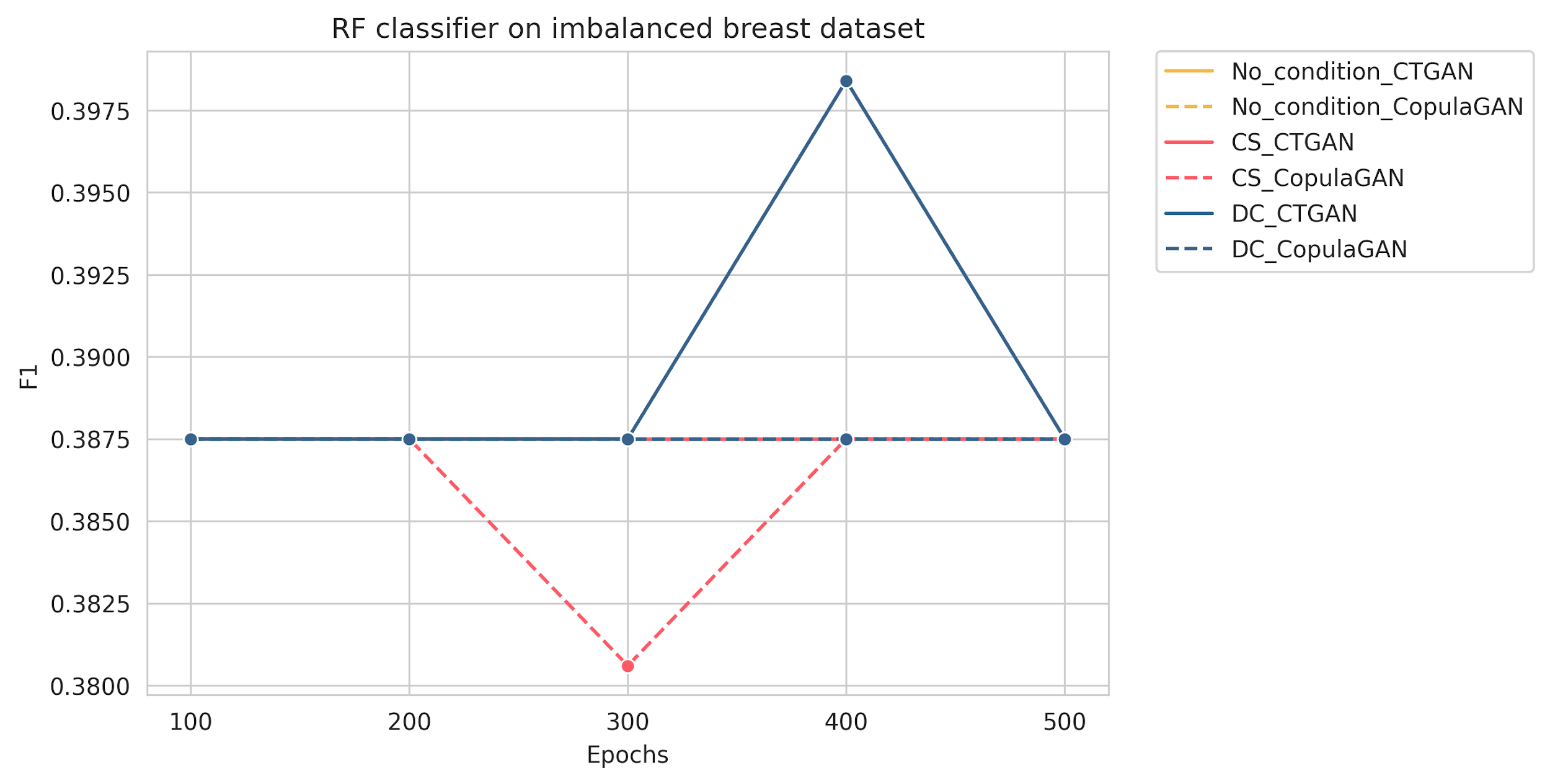


Figure A5-24. Effect of epoch on F1 in the imbalanced breast dataset using RF classifier.


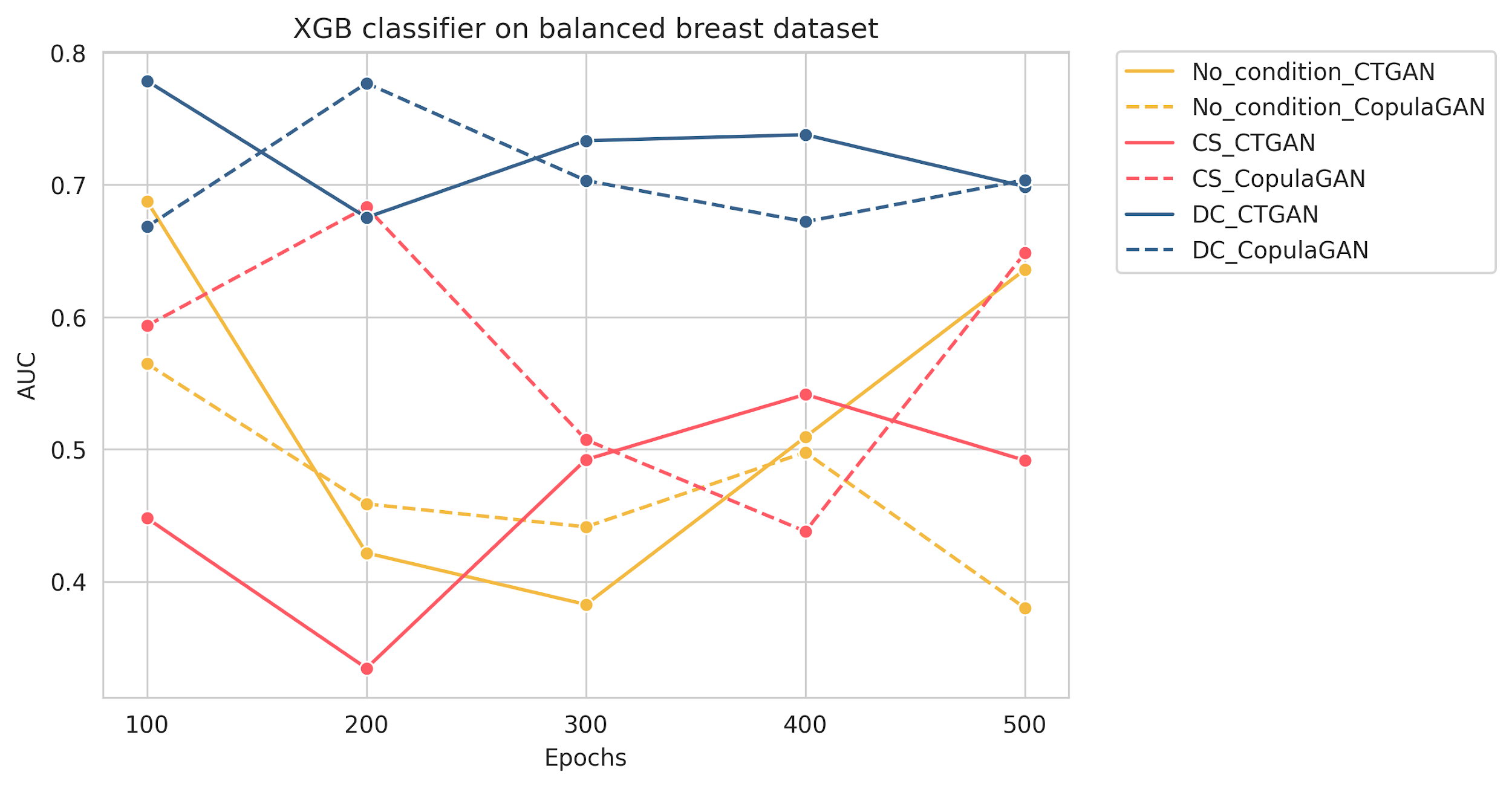


Figure A5-25. Effect of epoch on AUC in the balanced breast dataset using XGB classifier.


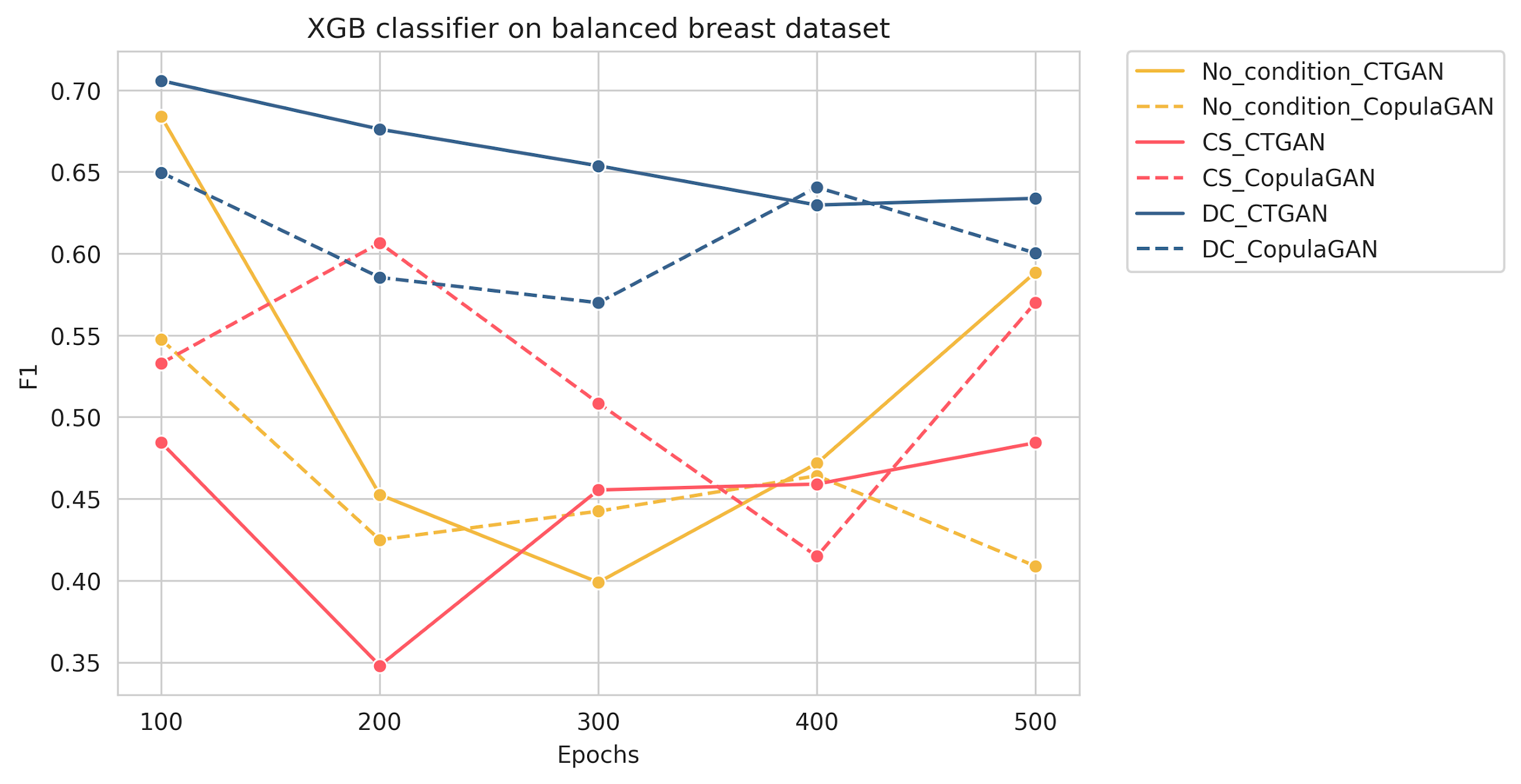


Figure A5-26. Effect of epoch on F1 in the balanced breast dataset using XGB classifier.


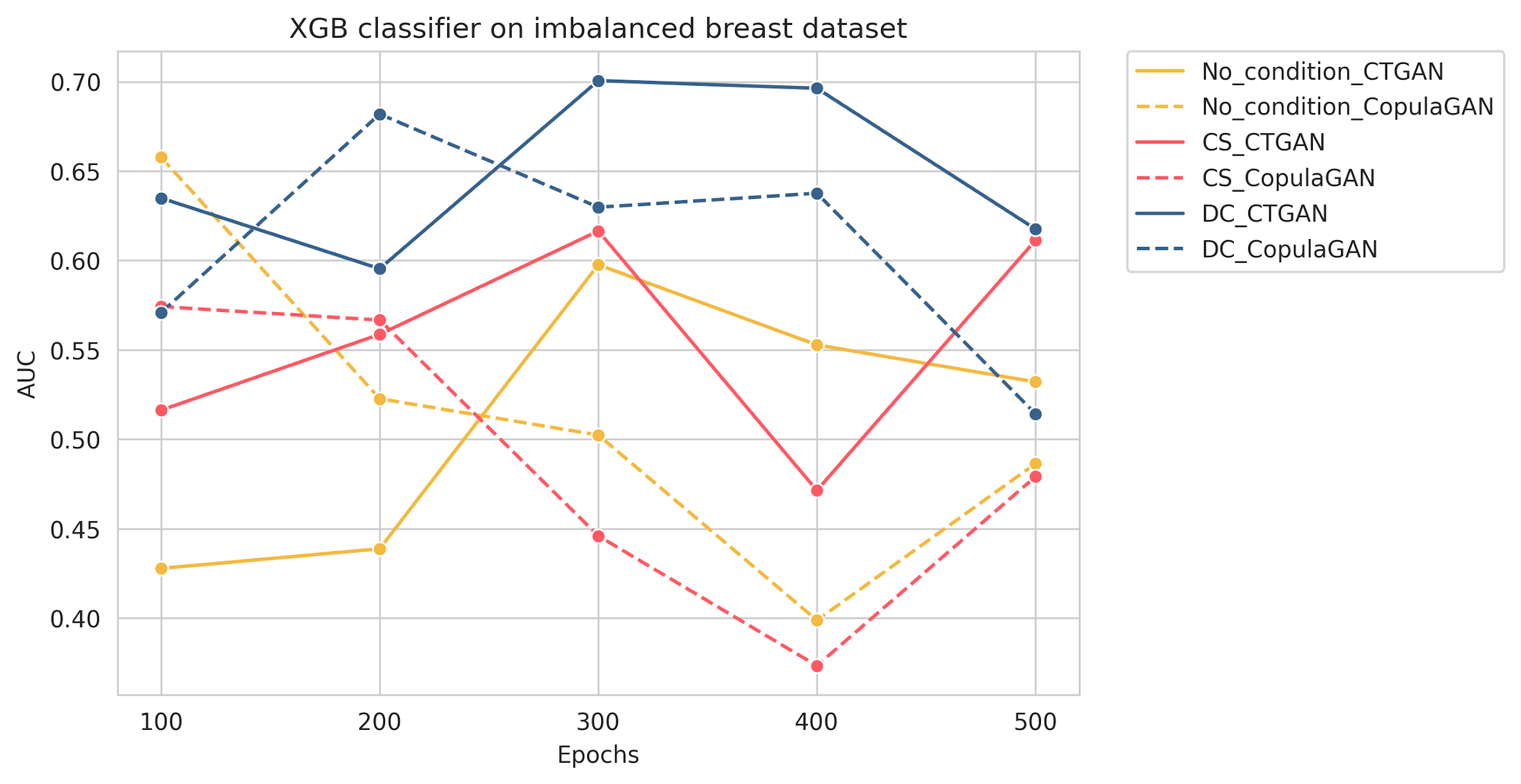


Figure A5-27. Effect of epoch on AUC in the imbalanced breast dataset using XGB classifier.


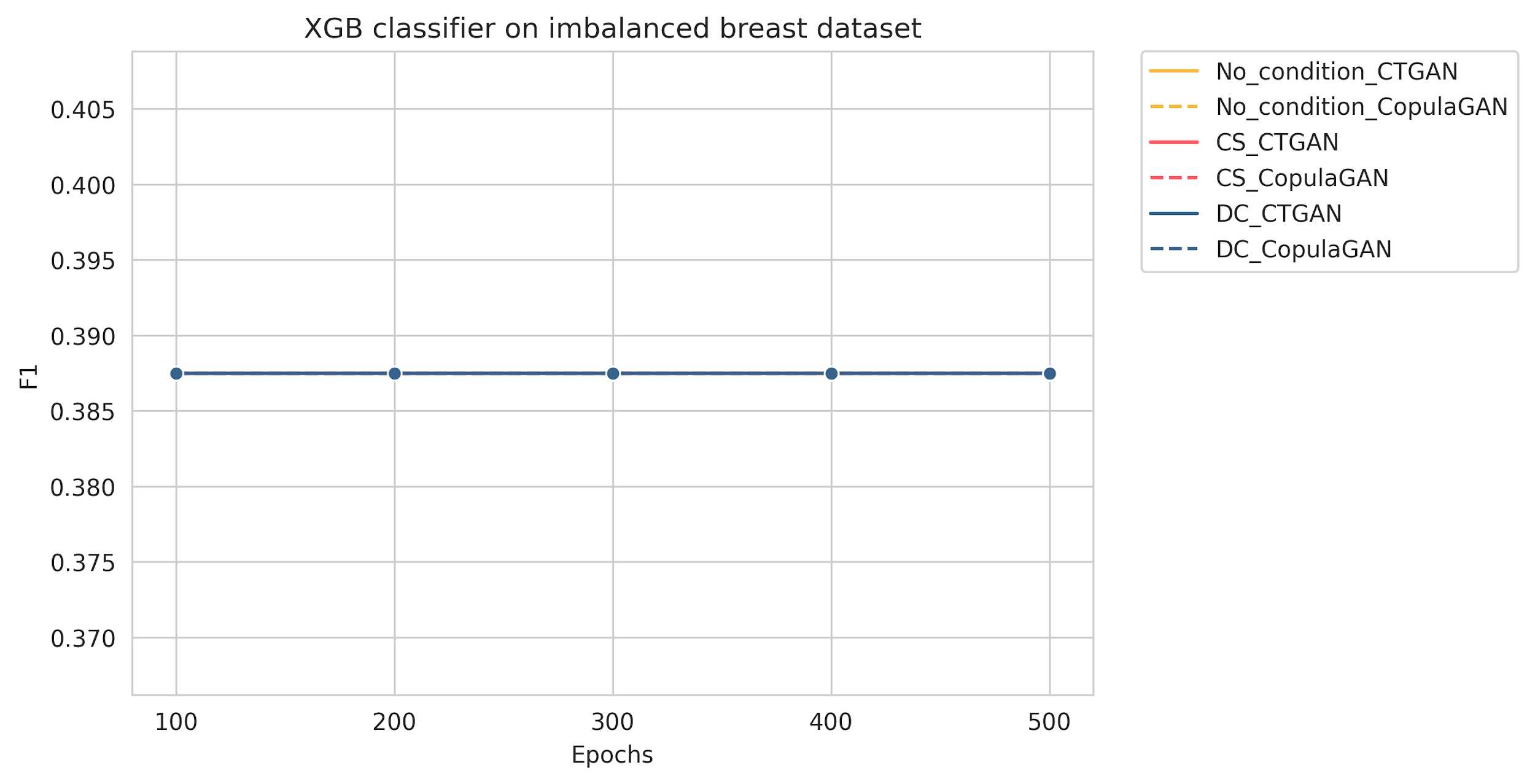


Figure A5-28. Effect of epoch on F1 in the imbalanced breast dataset using XGB classifier.


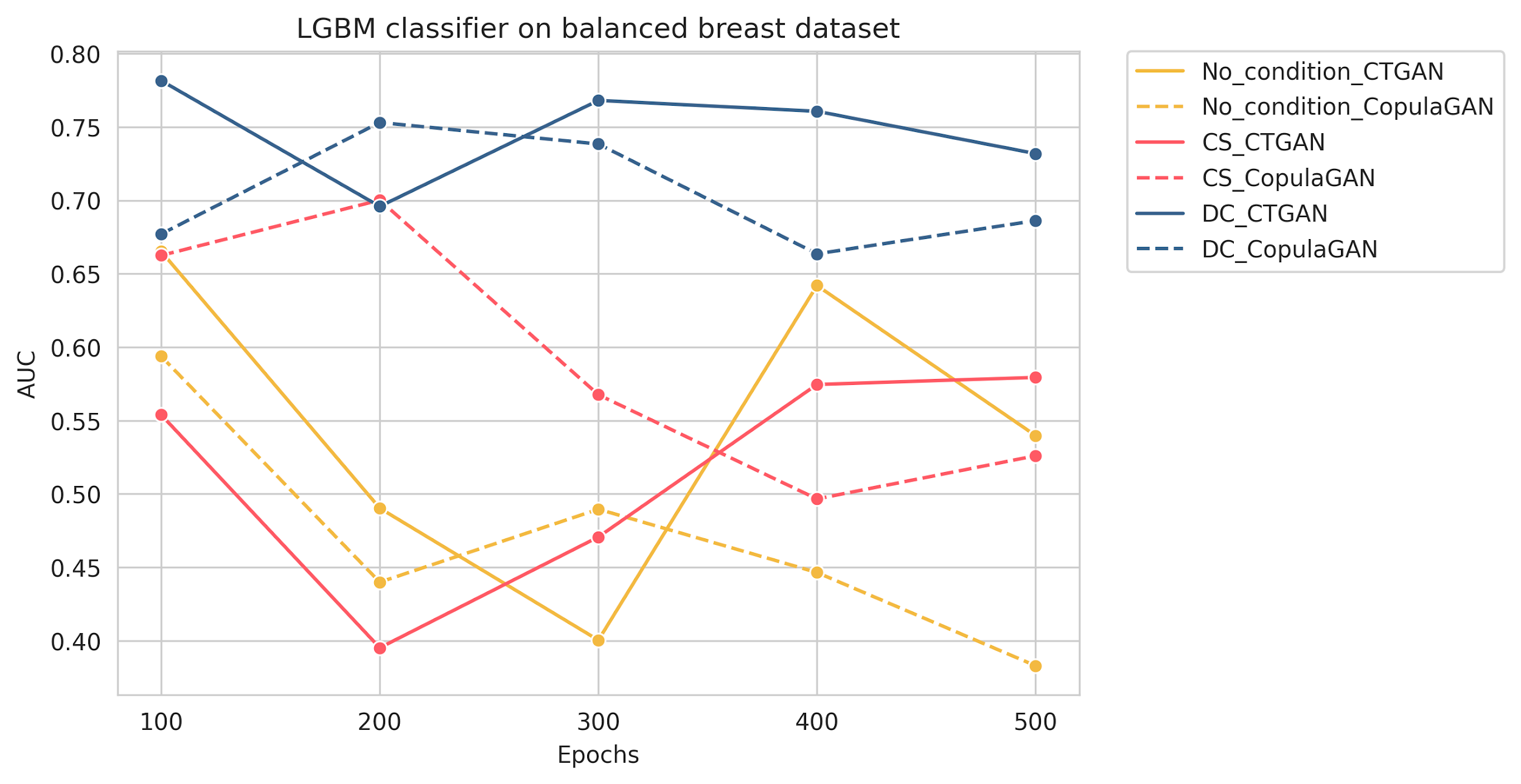


Figure A5-29. Effect of epoch on AUC in the balanced breast dataset using LGBM classifier.


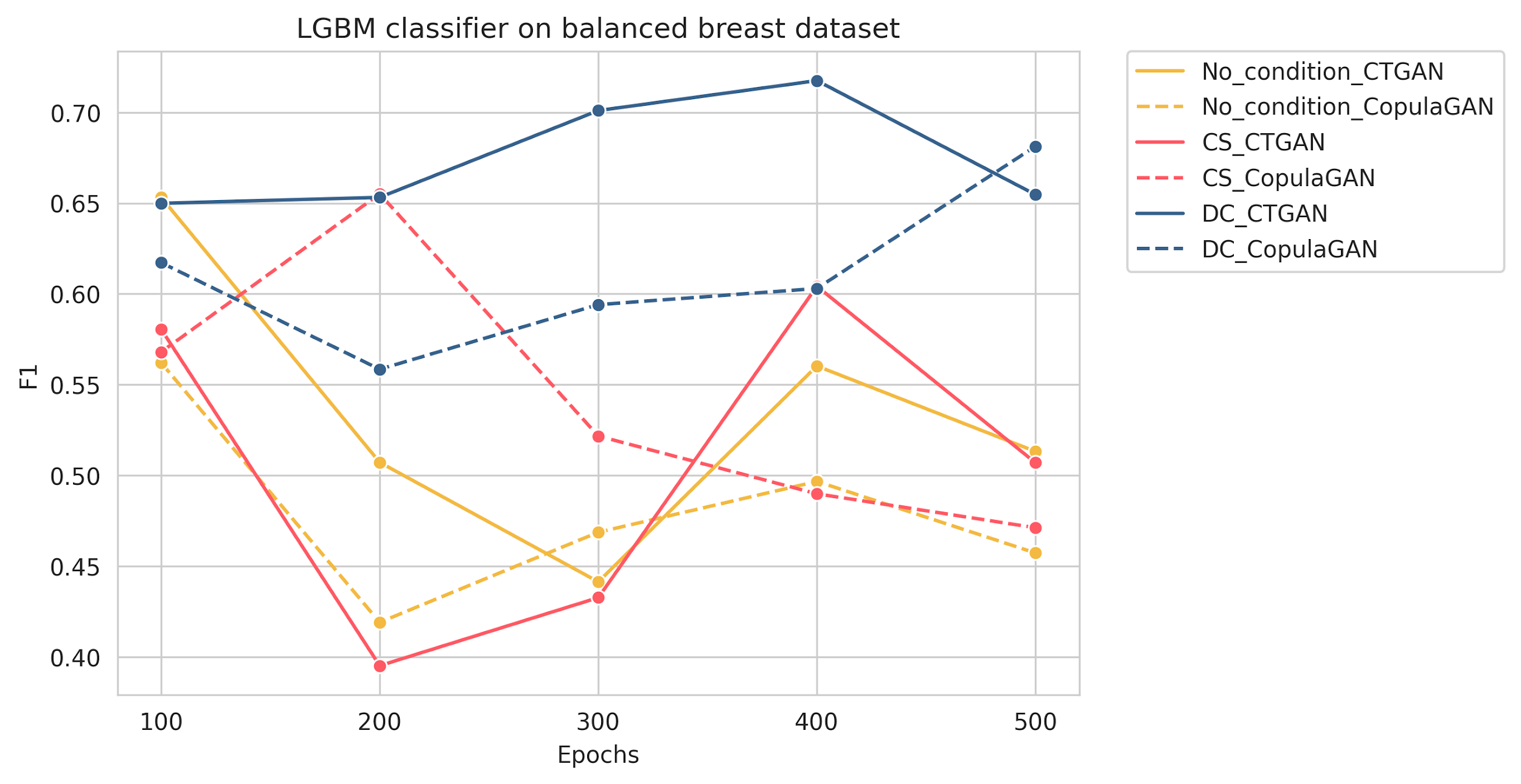


Figure A5-30. Effect of epoch on F1 in the balanced breast dataset using LGBM classifier.


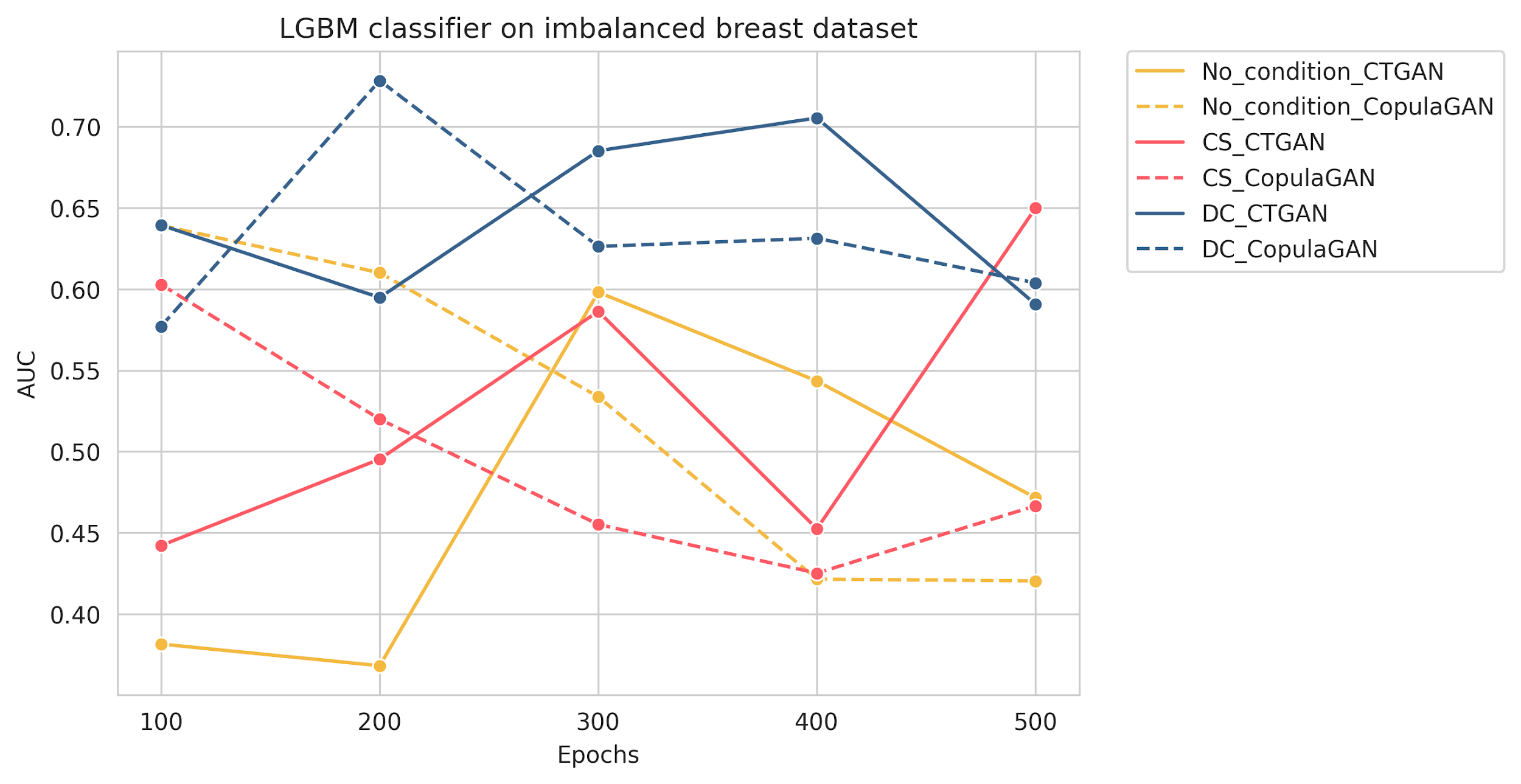


Figure A5-31. Effect of epoch on AUC in the imbalanced breast dataset using LGBM classifier.


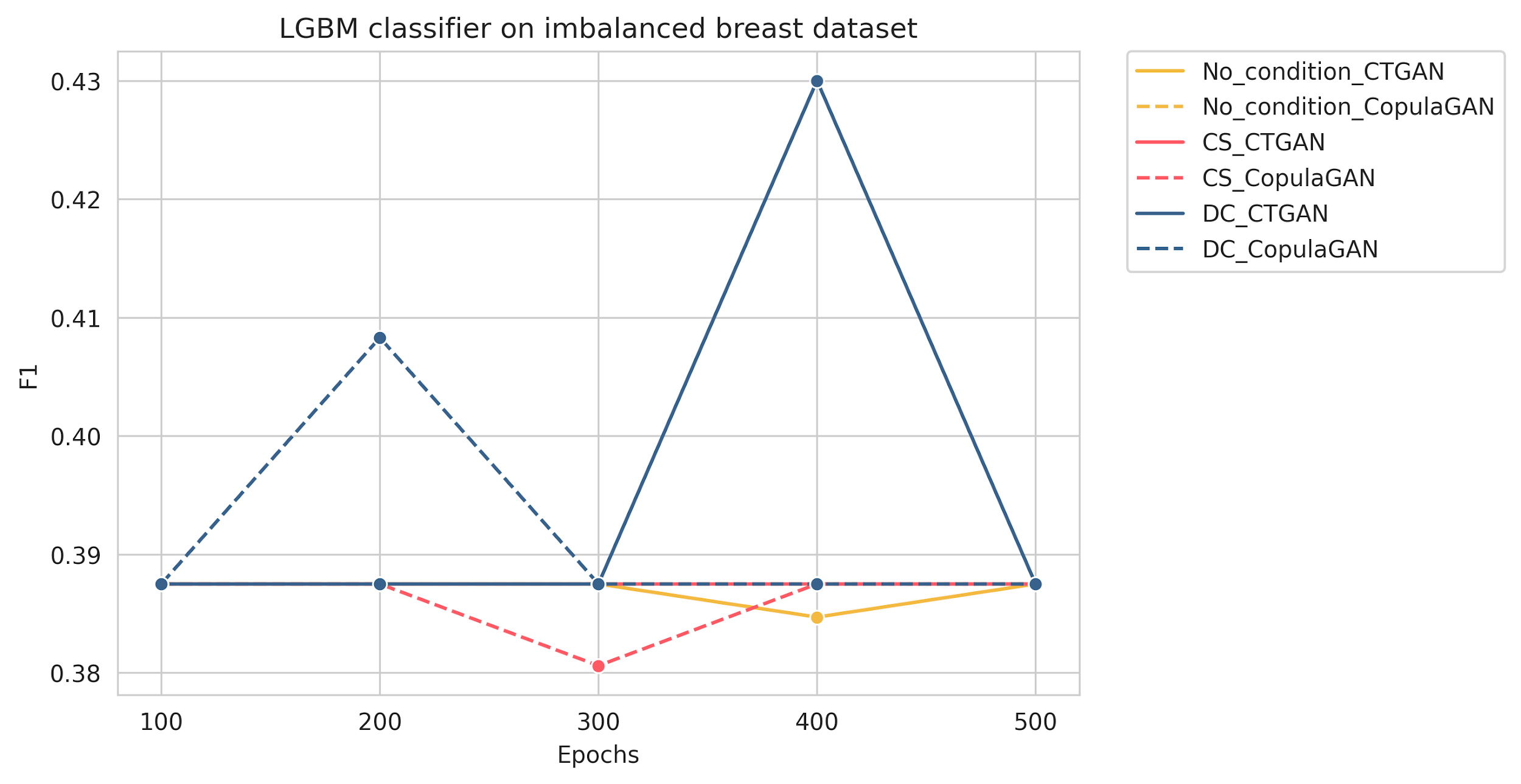


Figure A5-32. Effect of epoch on F1 in the imbalanced breast dataset using LGBM classifier.


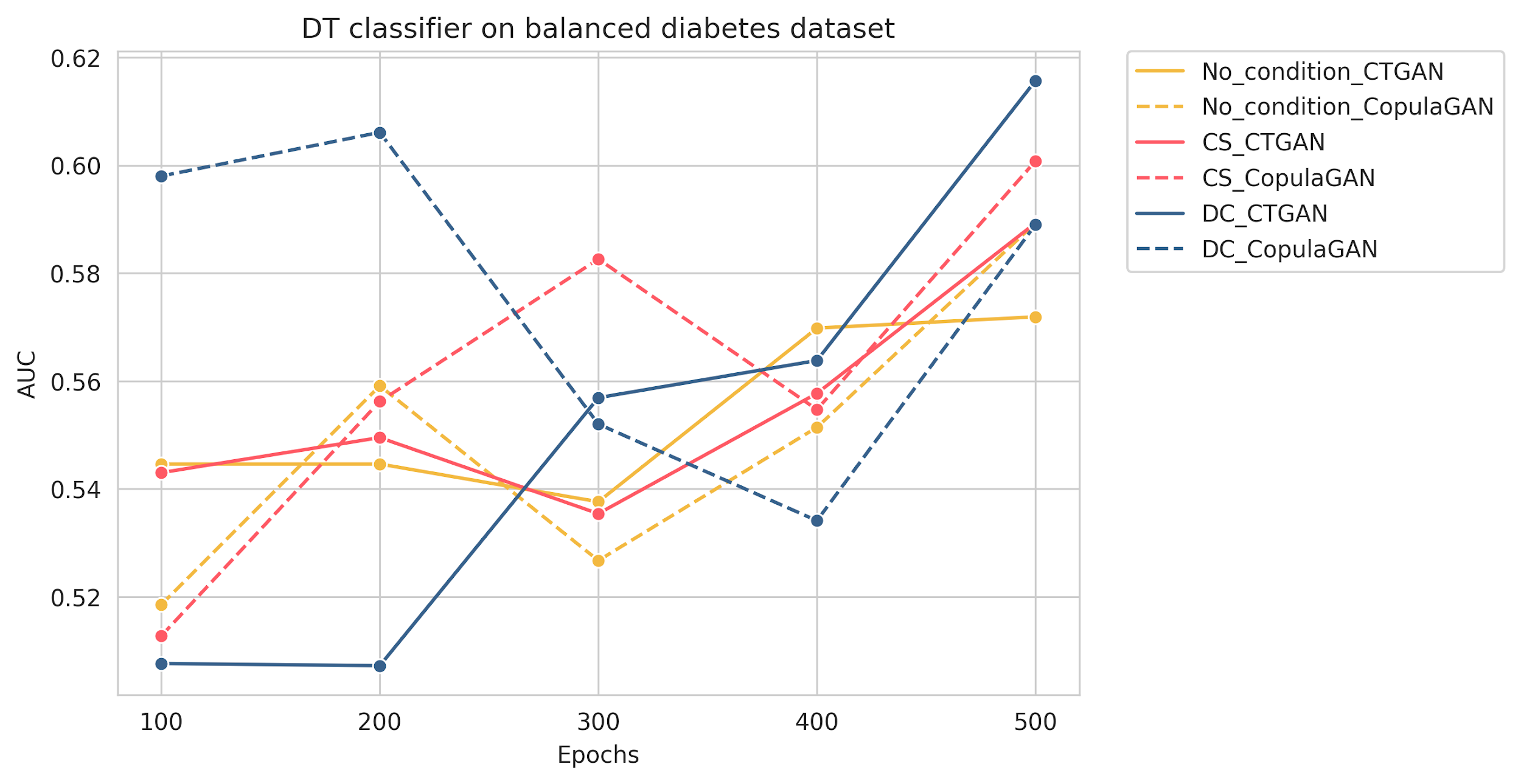


Figure A5-33. Effect of epoch on AUC in the balanced diabetes dataset using DT classifier.


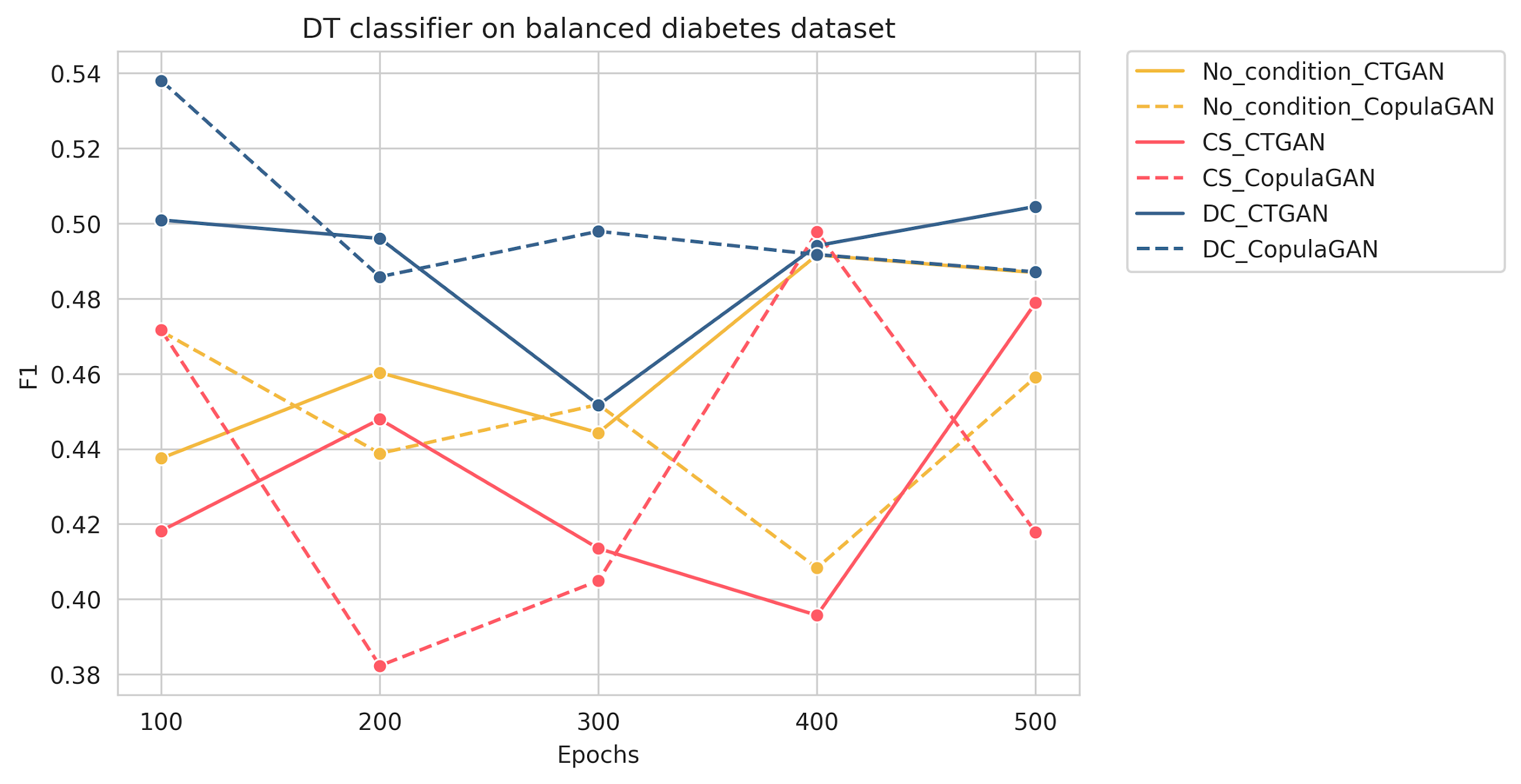


Figure A5-34. Effect of epoch on F1 in the balanced diabetes dataset using DT classifier.


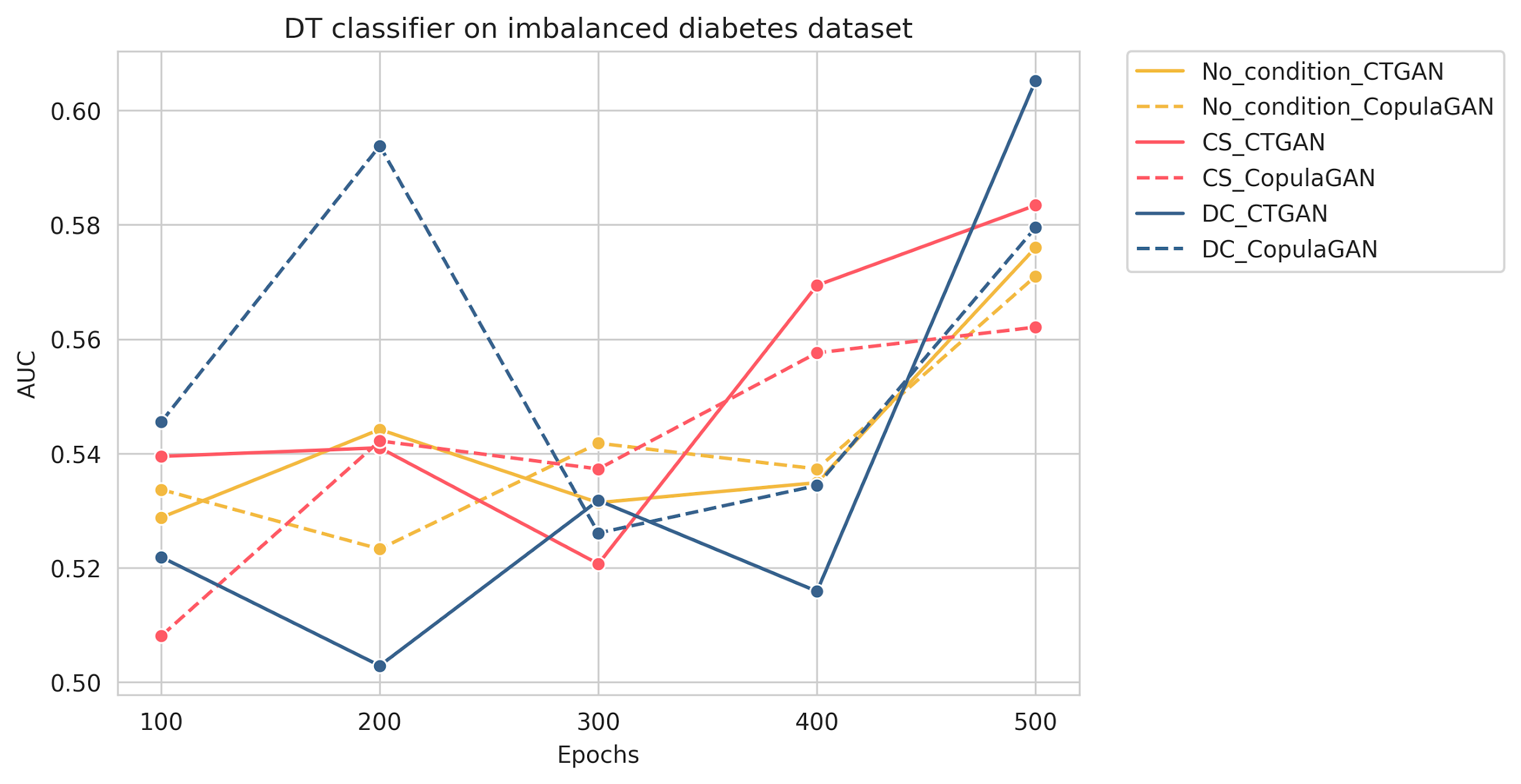


Figure A5-35. Effect of epoch on AUC in the imbalanced diabetes dataset using DT classifier.


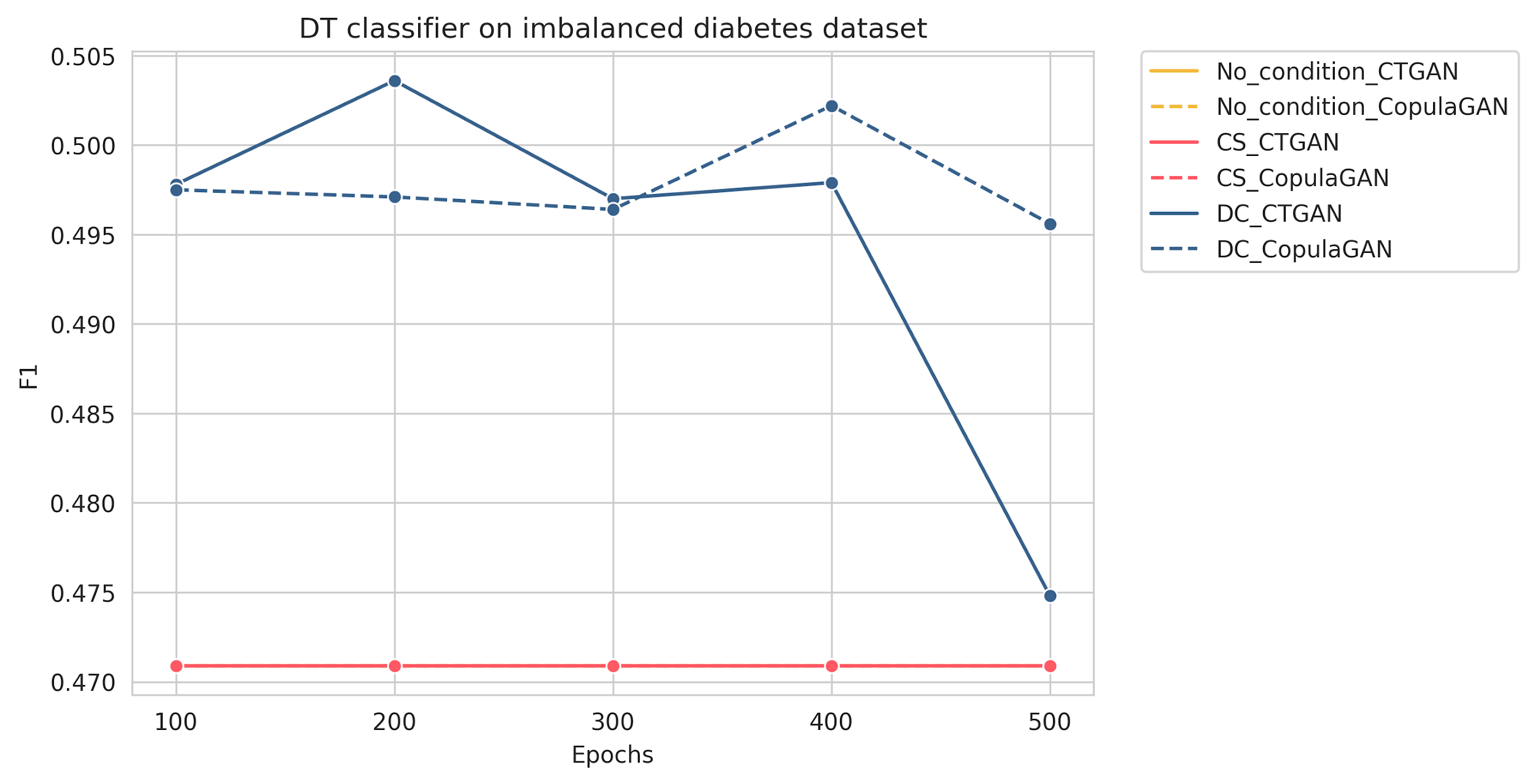


Figure A5-36. Effect of epoch on F1 in the imbalanced diabetes dataset using DT classifier.


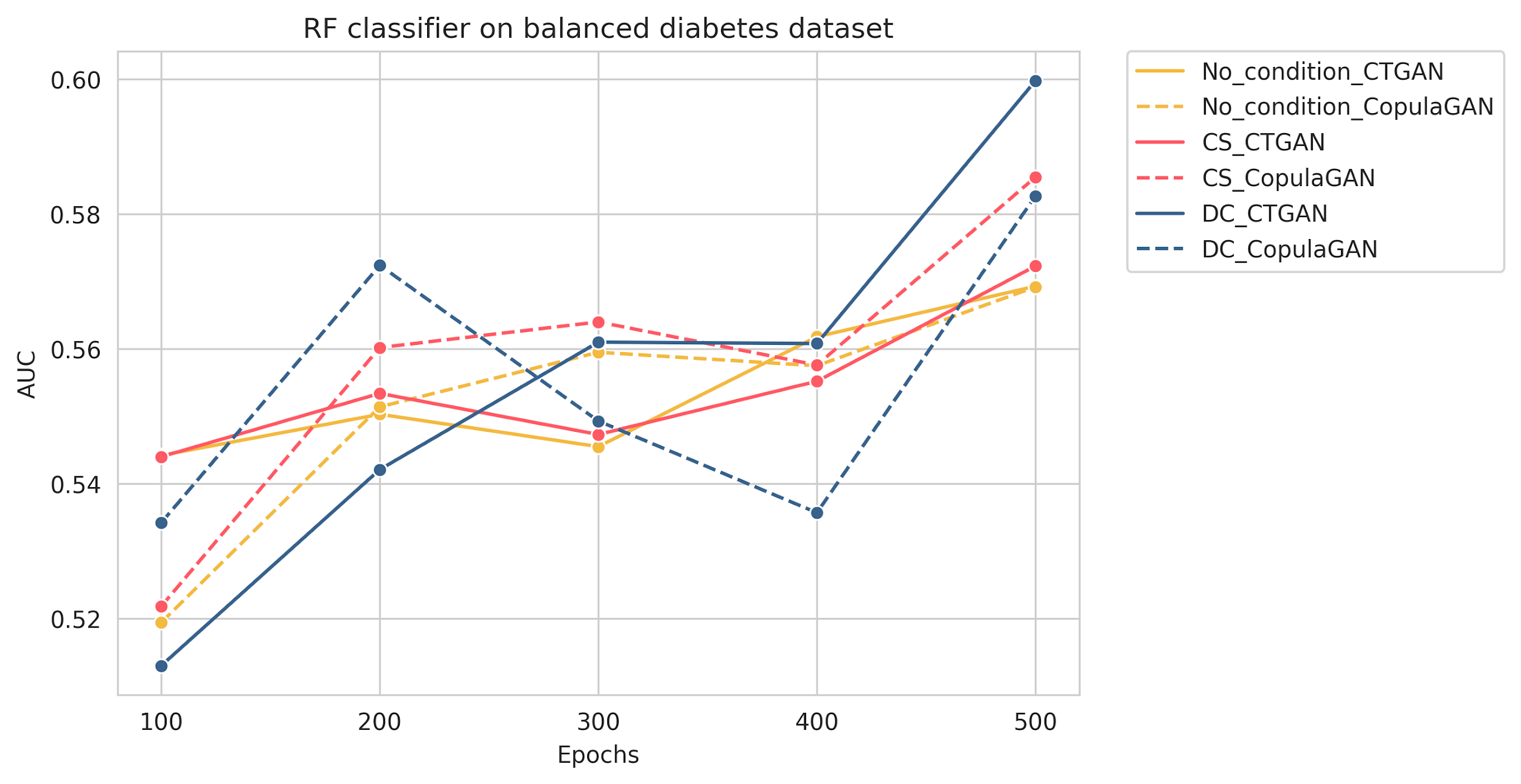


Figure A5-37. Effect of epoch on AUC in the balanced diabetes dataset using RF classifier.


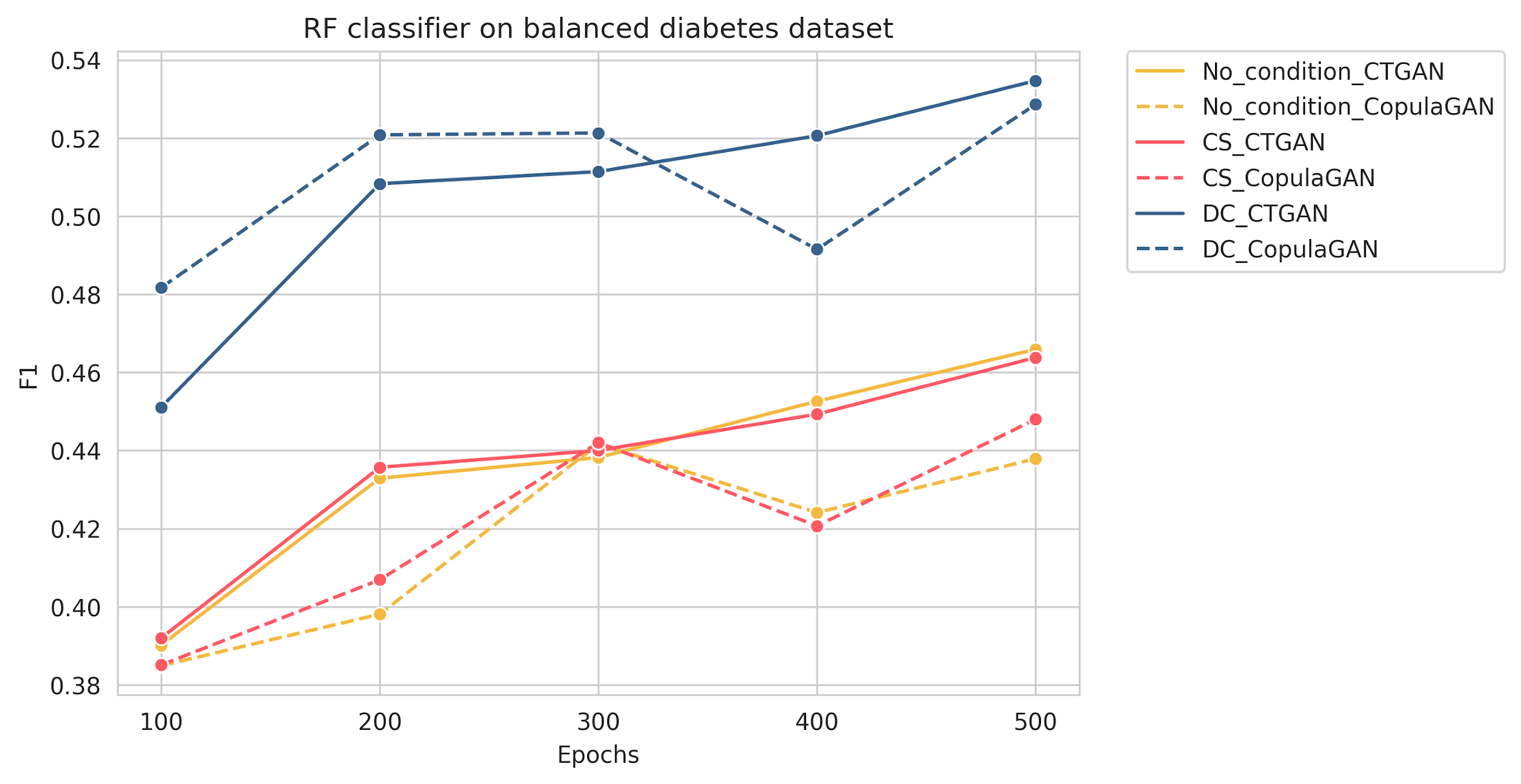


Figure A5-38. Effect of epoch on F1 in the balanced diabetes dataset using RF classifier.


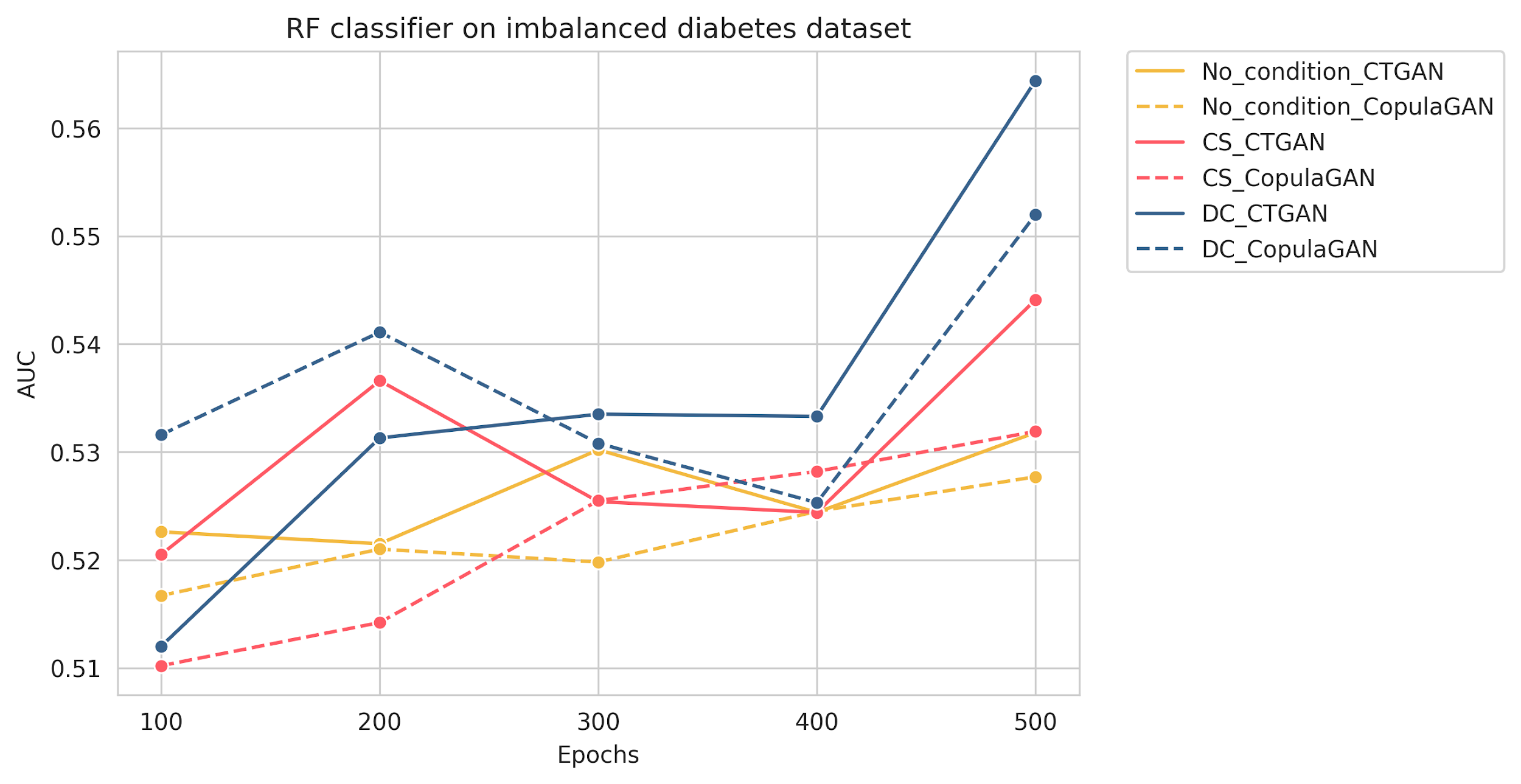


Figure A5-39. Effect of epoch on AUC in the imbalanced diabetes dataset using RF classifier.


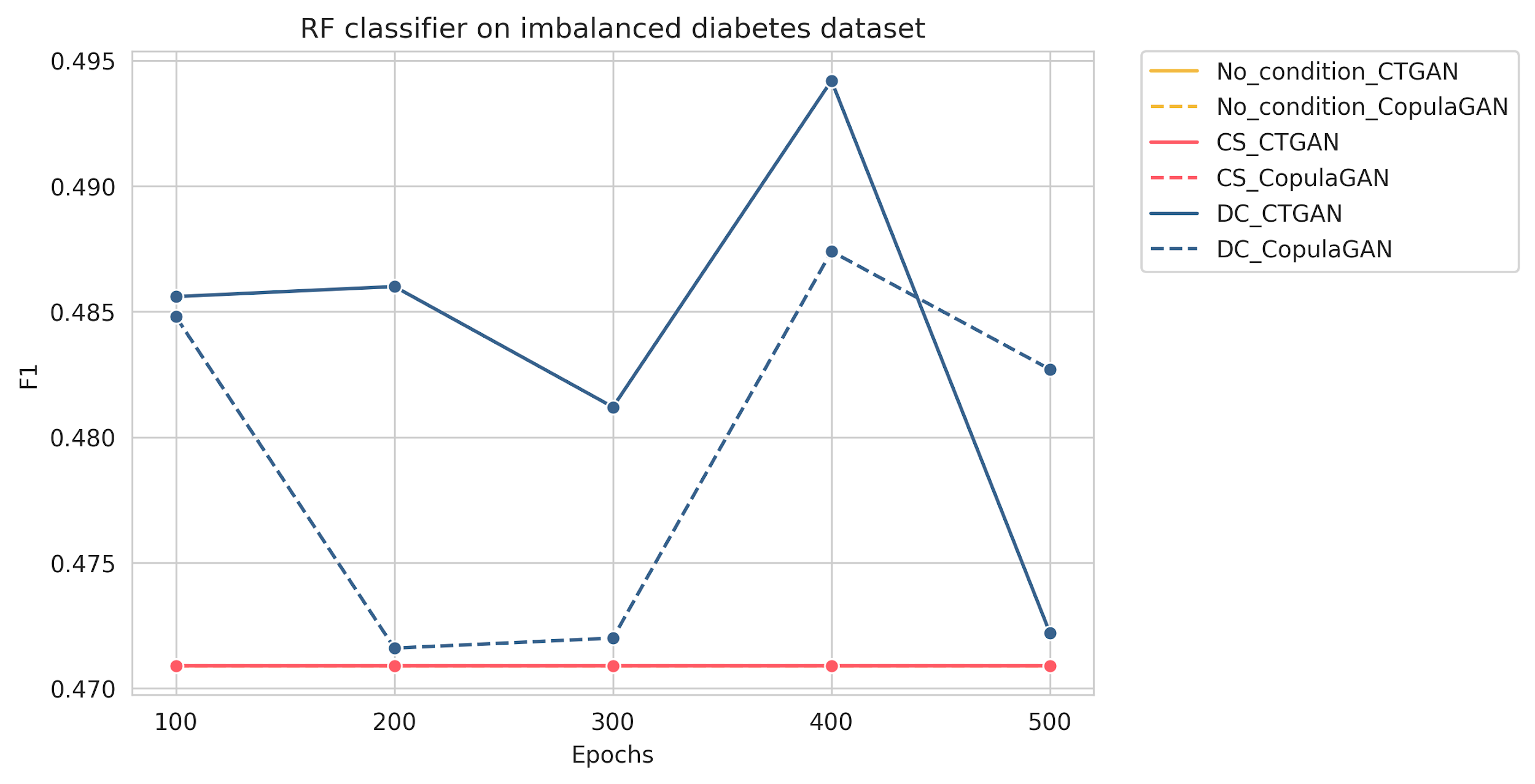


Figure A5-40. Effect of epoch on F1 in the imbalanced diabetes dataset using RF classifier.


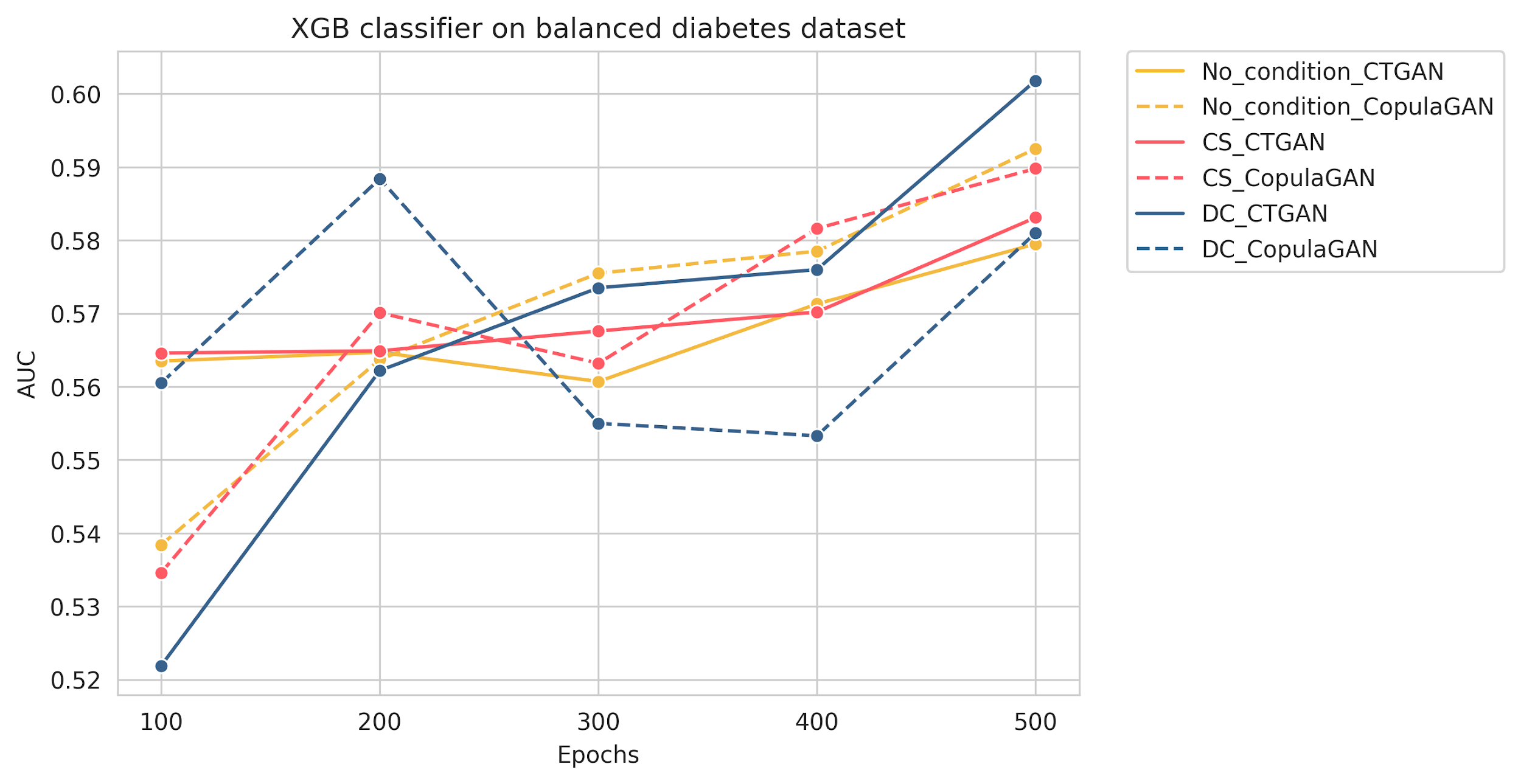


Figure A5-41. Effect of epoch on AUC in the balanced diabetes dataset using XGB classifier.


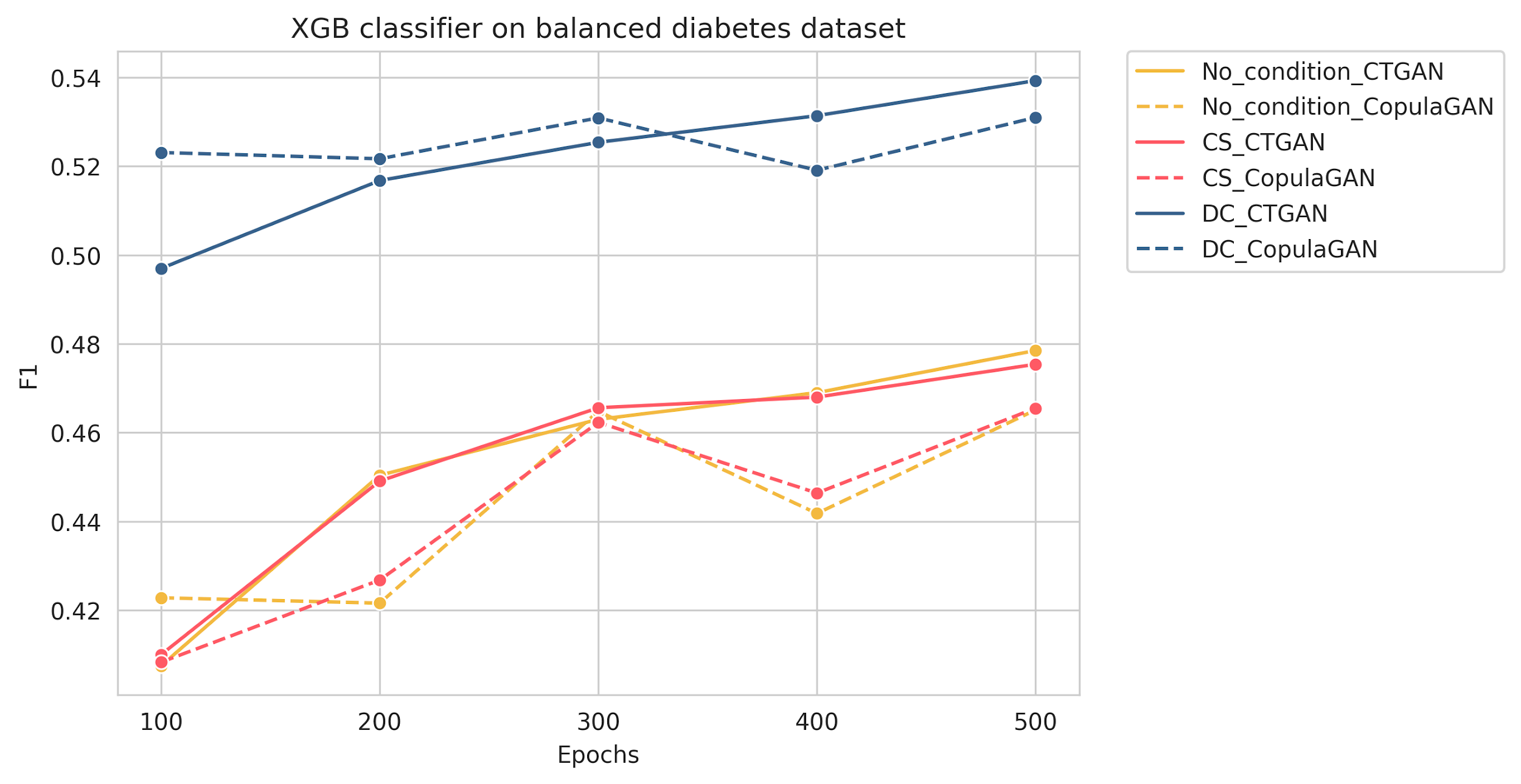


Figure A5-42. Effect of epoch on F1 in the balanced diabetes dataset using XGB classifier.


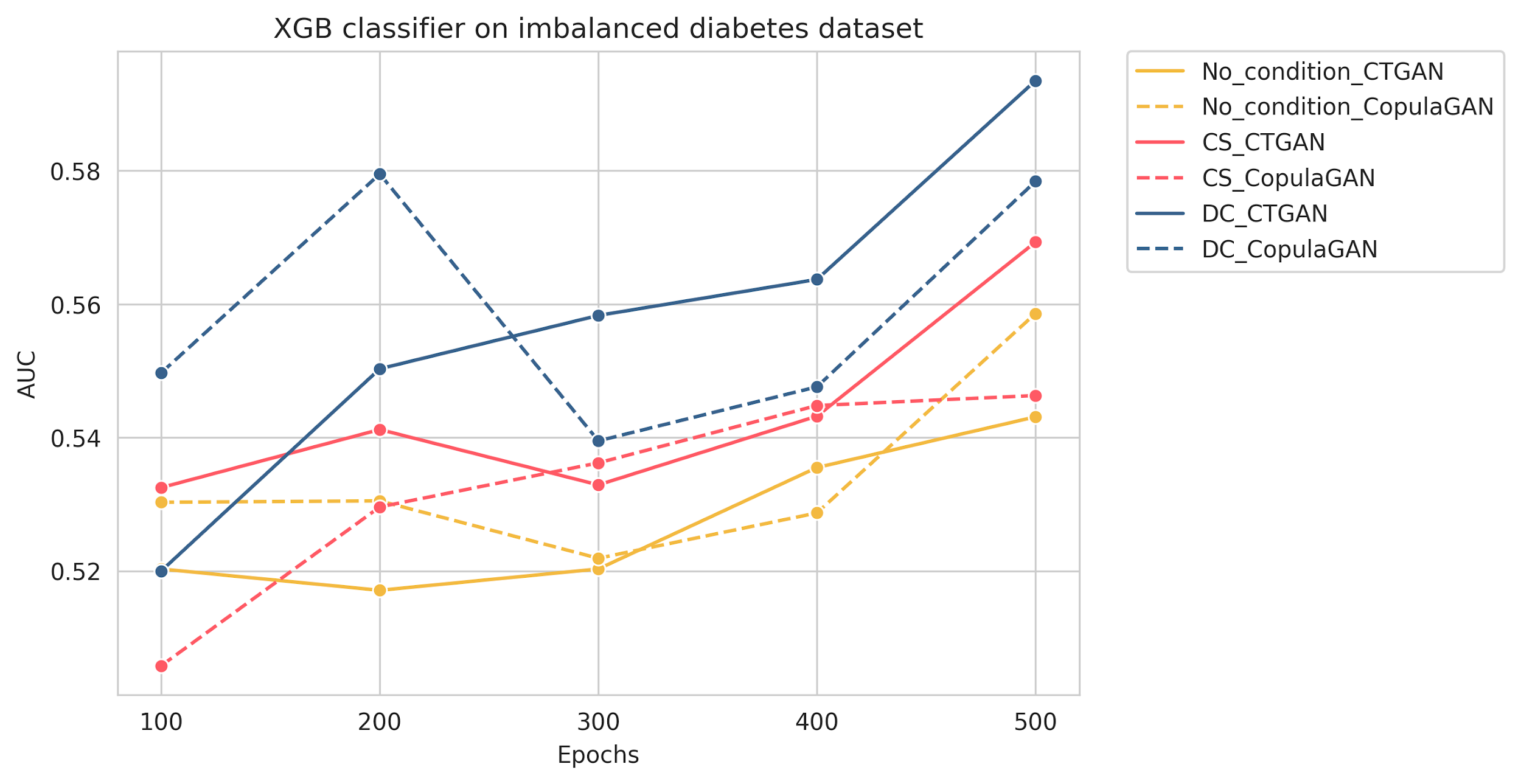


Figure A5-43. Effect of epoch on AUC in the imbalanced diabetes dataset using XGB classifier.


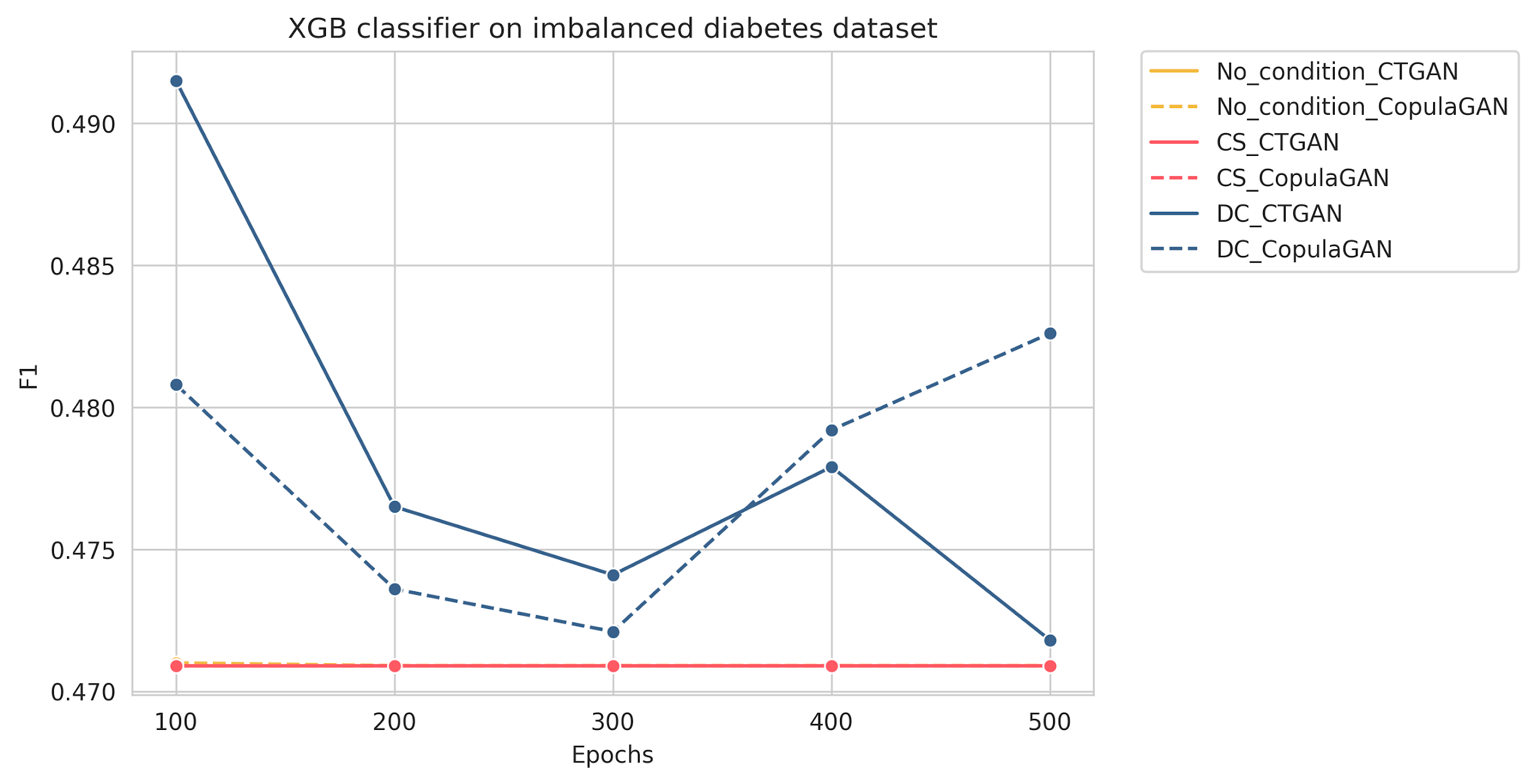


Figure A5-44. Effect of epoch on F1 in the imbalanced diabetes dataset using XGB classifier.


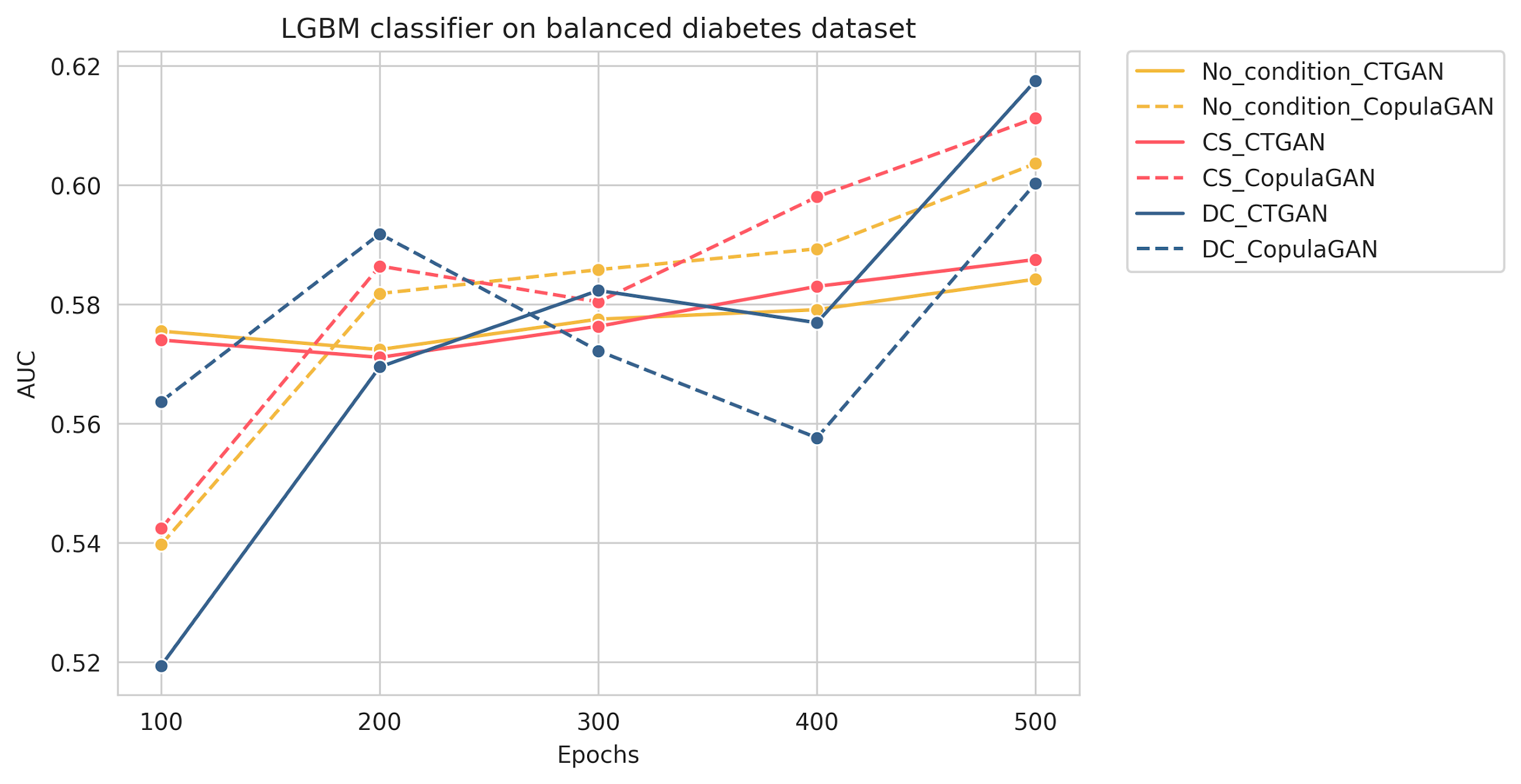


Figure A5-45. Effect of epoch on AUC in the balanced diabetes dataset using LGBM classifier.


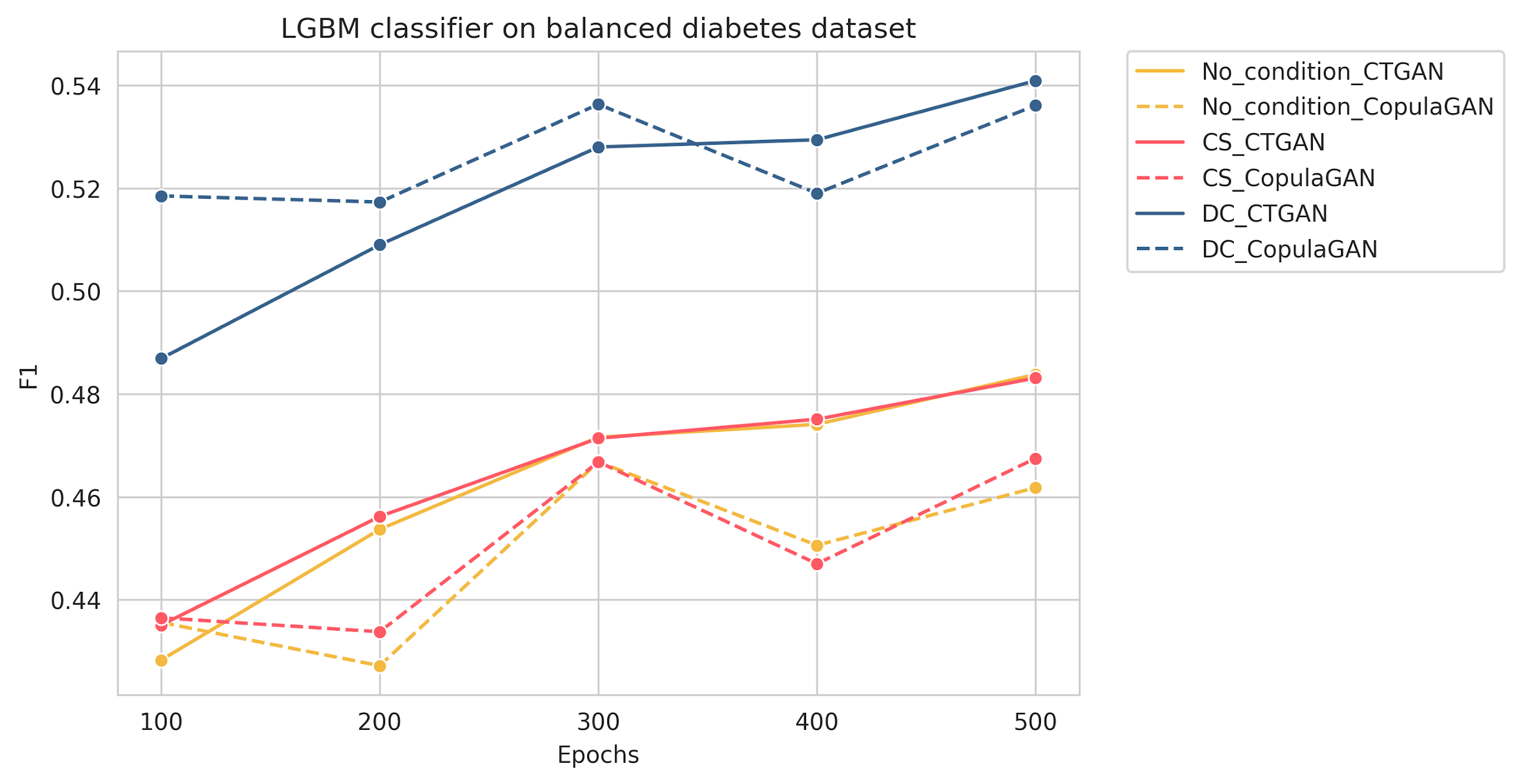


Figure A5-46. Effect of epoch on F1 in the balanced diabetes dataset using LGBM classifier.


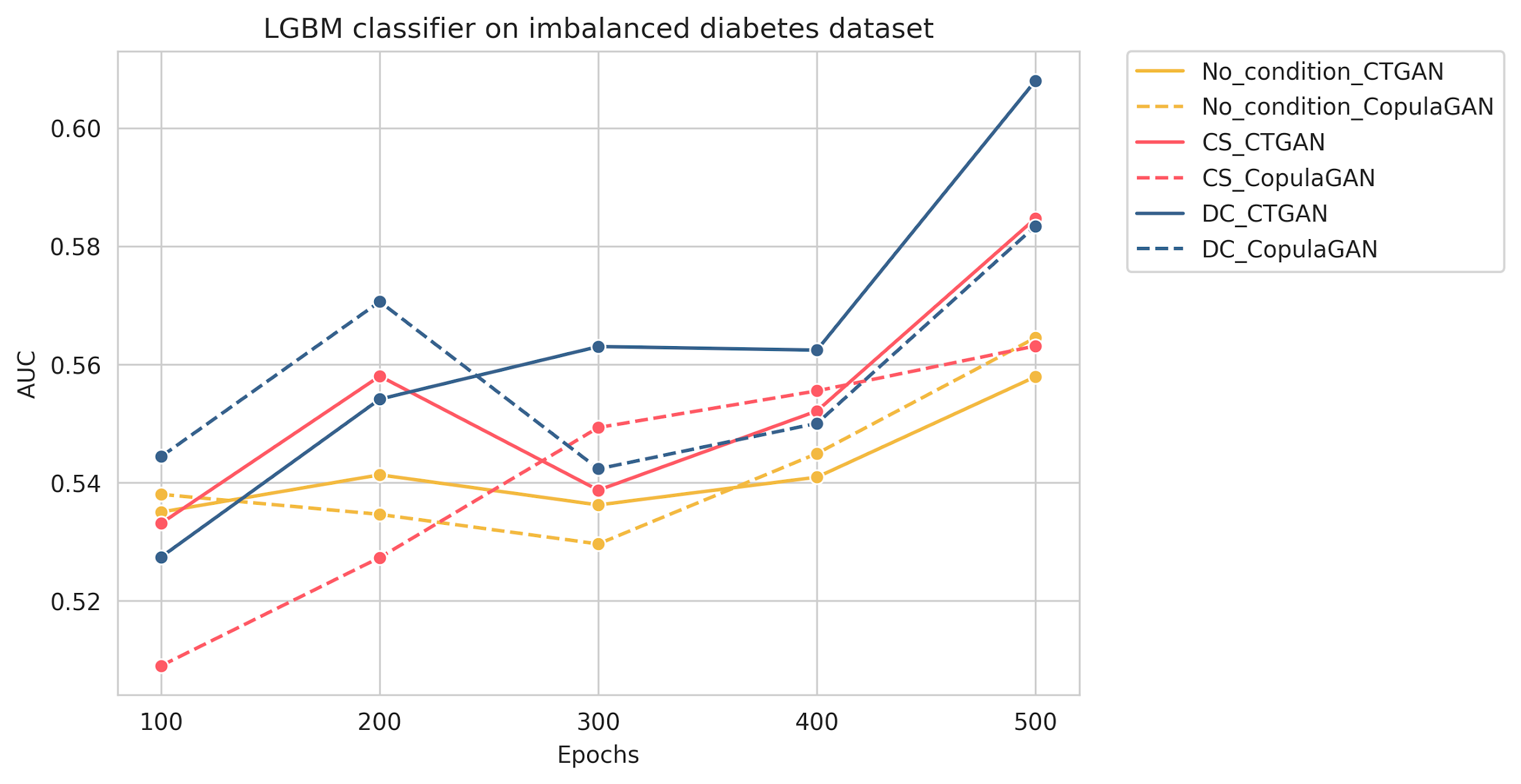


Figure A5-47. Effect of epoch on AUC in the imbalanced diabetes dataset using LGBM classifier.


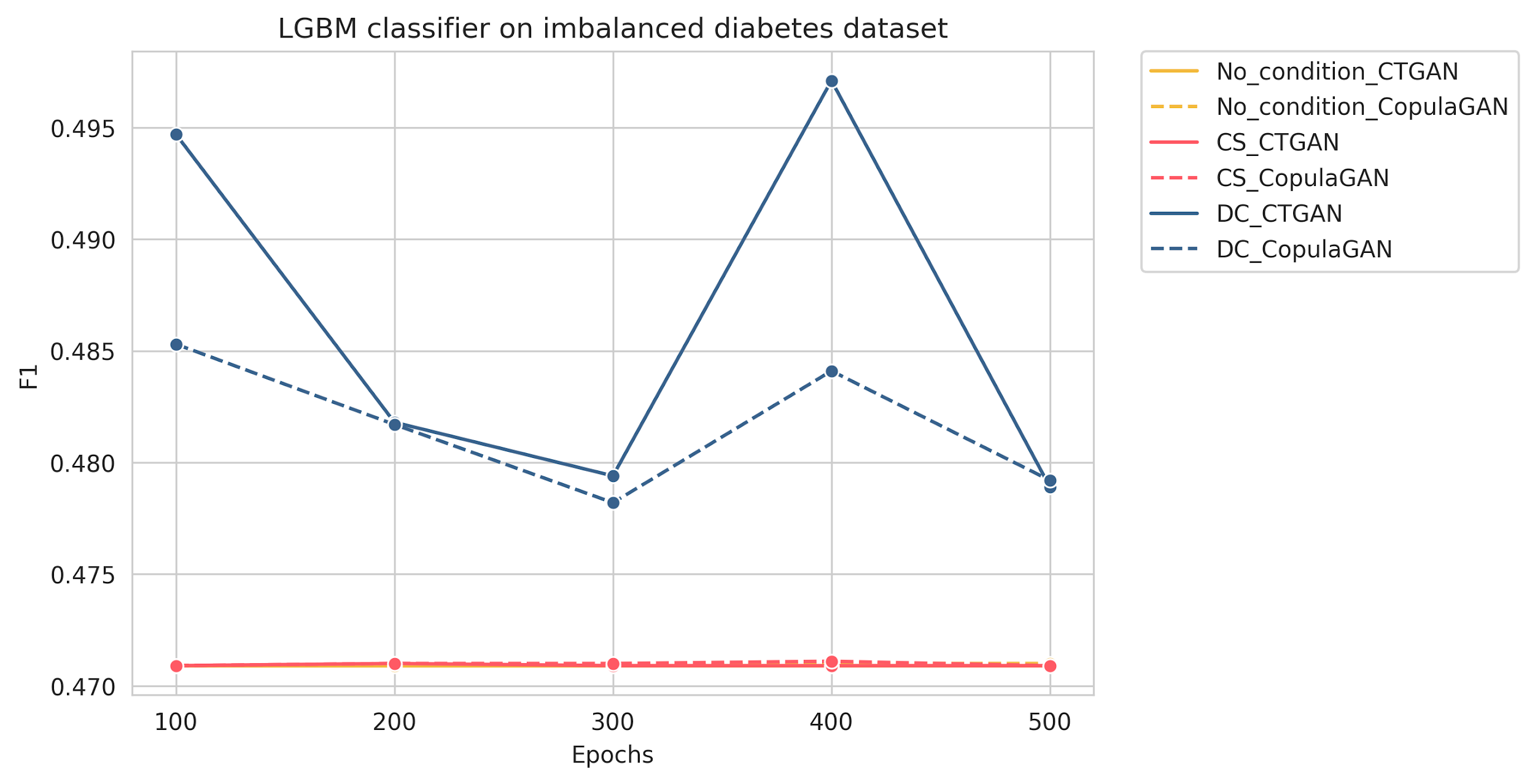


Figure A5-48. Effect of epoch on F1 in the imbalanced diabetes dataset using LGBM classifier.
